# Syntheses, Characterization, and Multifaceted Coordination Chemistry of Hydrazonido Titanium Complexes

**DOI:** 10.1021/acs.inorgchem.3c04301

**Published:** 2024-01-27

**Authors:** Kevin Schwitalla, Fares Sad, Marc Schmidtmann, Rüdiger Beckhaus

**Affiliations:** Institut Für Chemie, Carl von Ossietzky Universität Oldenburg, D-26111 Oldenburg, Federal Republic of Germany

## Abstract

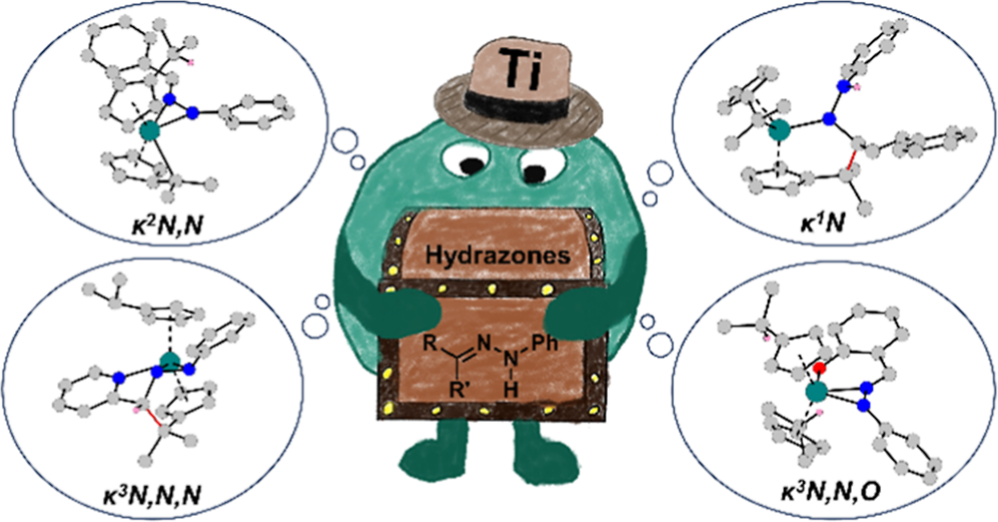

The reaction of hydrazones with bis(π–η^5^:σ–η^1^-pentafulvene)titanium complexes leads to both hydrazonido and hydrazido complexes depending on the interaction of the hydrazone with the fulvene ligand of the metal complex. The molecular structures mostly reveal κ^2^*N*,*N* side-on coordination of the hydrazonido ligand due to the deprotonation of the N–H bond by one of the fulvene moieties. Instead of deprotonation, the reaction of the bis(adamantylidene fulvene)titanium complexes with cinnamon aldehyde phenylhydrazone leads to κ^1^*N* coordination. By using donating groups in the backbone of the hydrazone ligands, there are exceptions to this coordination mode due to the insertion of the C=N double bond into the Ti–C_exo_ bond of the pentafulvene moiety. Using 2-pyridinecarboxaldehyde phenylhydrazone, a formal κ^3^*N*,*N*,*N* ligand system is formed by the coordination of the pyridine nitrogen atom to the metal center via consecutive N–H deprotonation and insertion. Finally, the use of salicylaldehyde phenylhydrazone ultimately produces a complex with the κ^3^*N*,*N*,*O* coordination mode by double deprotonation of the hydrazone N–H and O–H functions. Because of its slow conversion to the final product, the intermediate was isolated as an insertion product with consecutive O–H deprotonation, showing a κ^2^*N*,*O* coordination mode of the hydrazido ligand.

## Abstract 2

Reactions of bis(π−η^5^:σ−η^1^-pentafulvene)titanium complexes with a series of hydrazones are described. The different coordination modes of the resulting hydrazonido and hydrazido complexes were determined via NMR spectroscopy and single-crystal X-ray diffraction.

## Introduction

**Scheme 1:**
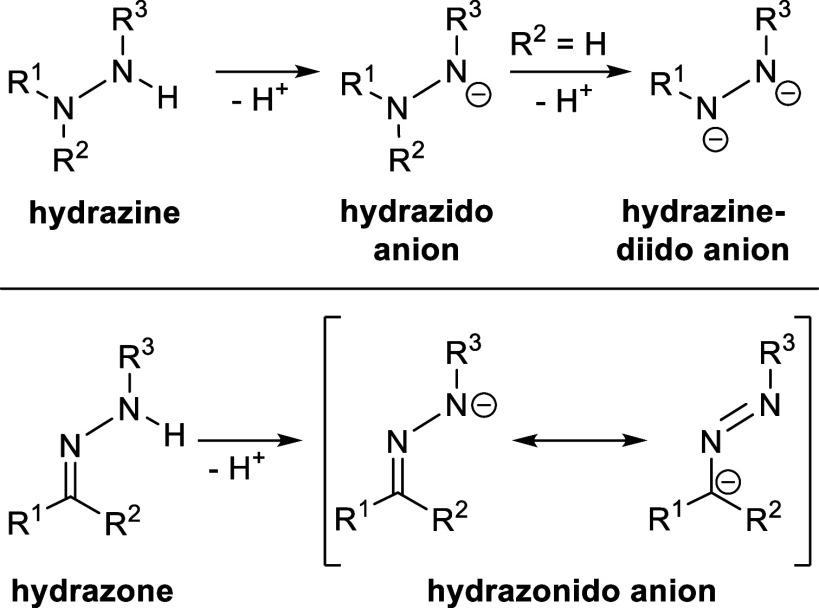
Structural Motifs of Hydrazines and Hydrazones and Their Respective Anions

Hydrazones are condensation products of a ketone or aldehyde with hydrazines. In the past, hydrazones were indirectly used to analyze aldehydes and ketones via the DNP test with 2,4-dinitrophenylhydrazine (DNPH) to form crystalline hydrazones with sharp melting points.^1^ Nowadays, they are used as substrates in homogeneous catalysis for hydrogenation^2−4^ and hydroalkylation^5−7^ reactions or as a precursor for the catalytic synthesis of heteroaromatics.^8,9^ Hydrazone-based metal complexes are of recent interest and have various catalytic,^10,11^ analytical,^12,13^ and biomedical^14,15^ applications. Hydrazido ligands are structurally related to the hydrazonido ligands, and there are many examples of hydrazido complexes.^16^ The main difference between both classes is the C=N bond of the hydrazine, whereas hydrazines simply have an NR_2_ group (Scheme 1). Despite the facile synthesis and tunability of hydrazones, monoanionic hydrazonido ligands appear very rarely in the literature, and hydrazonido metal complexes remain scarce.^17−19^ Using a monosubstituted hydrazine such as phenyl hydrazine, the formed hydrazone is bifunctional due to the N–H bond and the C=N double bond. This is particularly interesting in regard of the bis(π–η^5^:σ–η^1^-pentafulvene)titanium complexes capable of both element–H activation and insertion reactions due to the nucleophilic C_exo_ atom of the pentafulvene moiety.^20^ One prominent example for the activation of N-based substrates is the simultaneous N–H and C–H activation process in the titanium-catalyzed hydroaminoalkylation.^21^ Therefore, a broad range of coordination modes is possible, depending on how the complexes react with the respective hydrazone.

**Figure 1:**
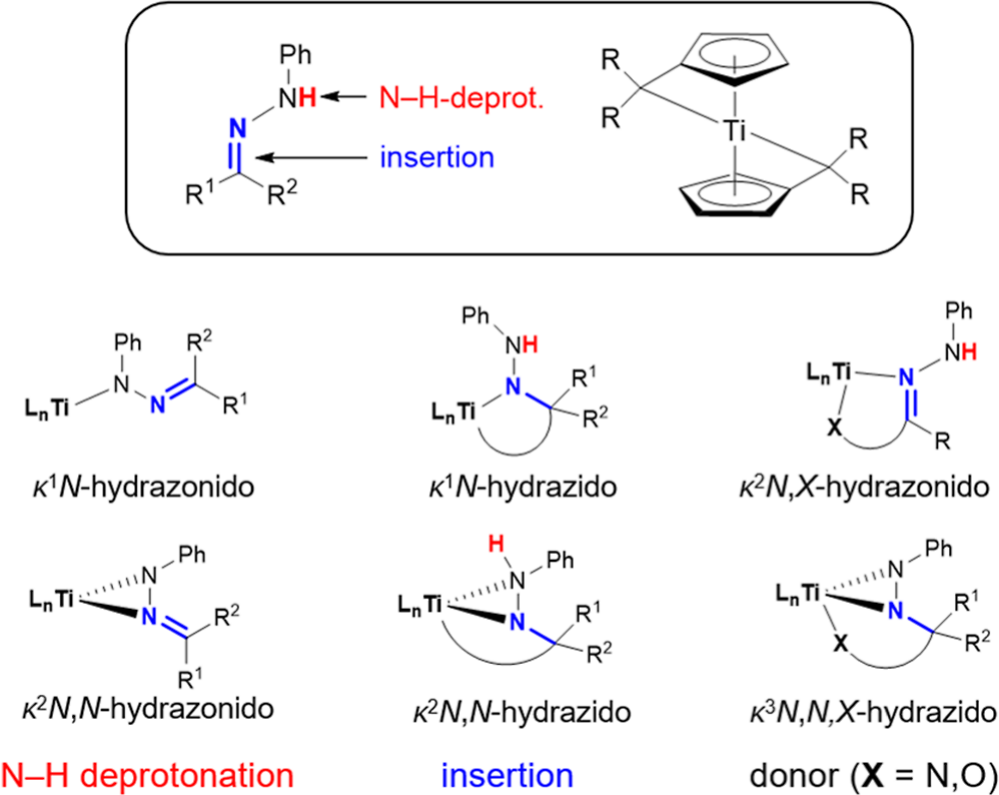
Possible coordination modes following the reaction of bis(π–η^5^:σ–η^1^-pentafulvene)titanium complexes with hydrazones.

Generally, deprotonation of the N–H bond should produce hydrazonido complexes, whereas insertion of the N=C double bond into the Ti–C_exo_ bond leads to hydrazido complexes. Donor functions in the backbone or additional deprotonation by the second fulvene moiety may provide further coordination modes (Figure 1). In this work, we report the reactions of bis(π–η^5^:σ–η^1^-pentafulvene)titanium complexes with a series of hydrazones. All products were comprehensively characterized, and the coordination modes were determined by single-crystal X-ray diffraction.

## Results and Discussion

**Scheme 2:**
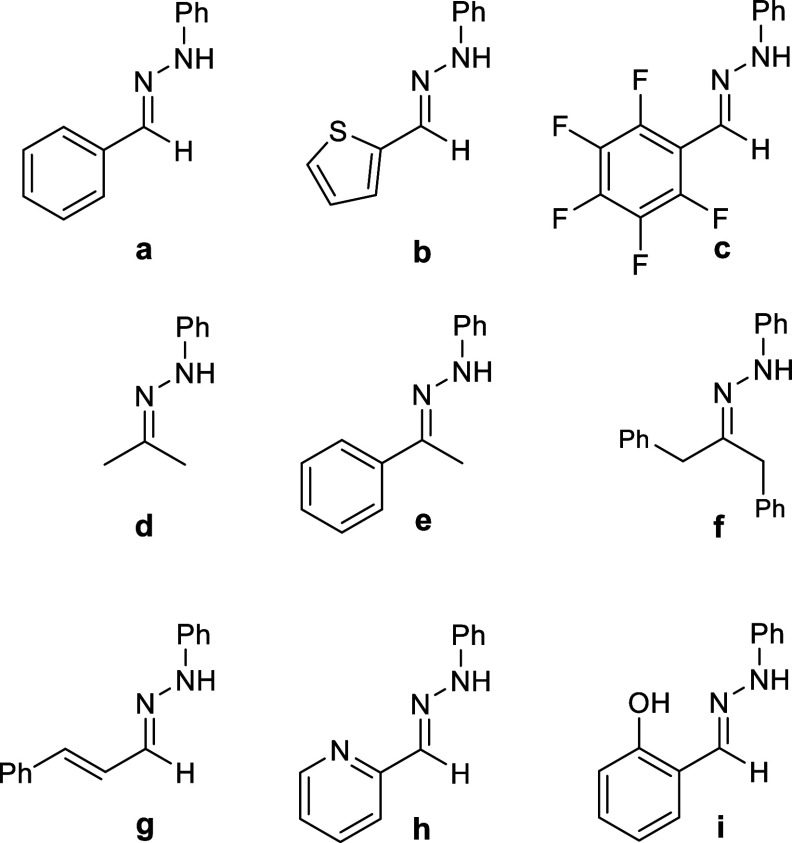
Scope of the Phenylhydrazones **a**–**i** Used in This Work Reactions with the hydrazones **a**–**f** yielded the side-on κ^2^*N*,*N*-hydrazonido complexes **Ti1a**–**Ti1f** and **Ti2a**,**b**,**d**,**e** (Scheme 3). The reaction of **Ti2** with **c** and **f** resulted in a complex mixture of products that were not further characterized.

The hydrazones **a–i** (except **d**)^22^ were synthesized according to a general procedure via condensation of phenyl hydrazine and the corresponding aldehyde or ketone.^23^ This series not only includes mostly hydrazones with alkyl- or aryl groups (**a**, **d**–**f**) but also heteroaromatics (**b**, **h**), a C_6_F_5_ group (**c**), and the phenyl hydrazones of cinnamon aldehyde (**g**) and salicyl aldehyde (**i**) (Scheme 2).

**Figure 2:**
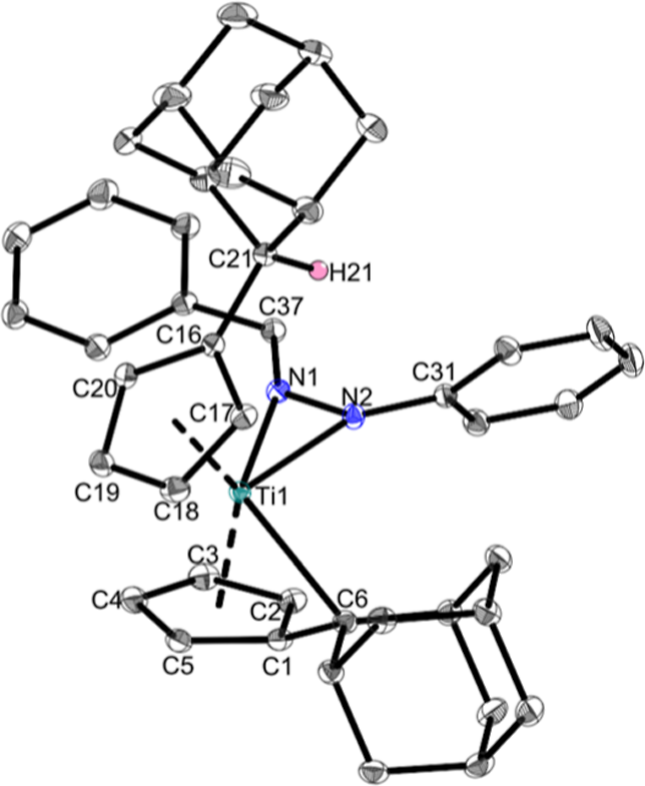
Molecular structure of complex **Ti1a**. Displacement ellipsoids are drawn at the 50% probability level. Redundant H atoms and solvent molecules have been omitted for clarity. Selected bond lengths (Å) and angles (deg): Ti1–N1 2.0999(6), Ti1–N2 2.0714(6), Ti1–C6 2.5077(7), N1–N2 1.3409(8), N1–C37 1.2995(9), N2–C31 1.4115(9), C1–C6 1.4263(10), N2–H21 2.408, Ti1–N1–N2 70.10(4), Ti1–C6–C1 85.41(4), Ti1–N1–C31 161.57(5), and Ct1–Ti1–Ct2 136.5 (Ct1 = centroid of C1–C5 and Ct2 = centroid of C16–C20).

The products were comprehensively characterized via NMR spectroscopy, and the coordination modes were determined by single-crystal X-ray diffraction. The ^1^H NMR spectrum shows eight different signals for the ring protons, indicating an asymmetry between the two ring systems as one of the C_exo_ atoms of the pentafulvene moieties is protonated. Importantly, the aldimine signals (**Ti1a-c** and **Ti2a,b**) are rather deshielded, with values between 7.65 and 8.03 ppm; hence, no additional insertion of the C=N double bond occurred. The ^15^N signals—measured via ^1^H,^15^N-HMBC spectra—show values between 282.4 and 304.3 ppm for the N^α^ atom and 160.5 and 200.5 ppm for the N^β^ atom by coupling with the respective aldimine protons or the methyl/methylene protons of the ketimines. The molecular structure of **Ti1a** (Figure 2) reveals fairly balanced Ti–N bond lengths of 2.0999(6) and 2.0714(6) Å for N1 and N2, respectively, which are shared by the other complexes **Ti1b**, **Ti1c**, **Ti1d**, and **Ti1f** (Table 1) and can be best described as single bonds according to the sum of covalent radii [Σ*r*_cov_(Ti–N) = 2.07 Å].^24^ This is rather uncommon for side-on κ^2^*N*,*N*-coordinating hydrazido complexes, which usually have a higher deviation between the bond lengths.^25−27^ One rare example of a side-on κ^2^*N*,*N* hydrazonido transition metal complex, namely, Cp_2_Er[η^2^–NHN=CPh_2_](THF),^18^ has less balanced bonds with 2.372(4) and 2.227(4) Å than **Ti1a** and its derivatives. The N–N bond lengths with values between 1.3285(15) Å and 1.3661(9) Å show double bond character [Σ*r*_cov_(N–N) = 1.42 Å,^24^ Σ*r*_cov_(N=N) = 1.20 Å].^28^ The Ti–C_exo_ bond of the remaining fulvene moiety [2.5077(7) Å] is significantly larger than that of the bis(pentafulvene)titanium complex **Ti1** [2.341(2) and 2.363(2) Å],^29^ indicating a lowered reactivity. This is backed up by the lack of follow-up chemistry of **Ti1a** and derivatives as the complexes were reisolated after treatment with ketones, nitriles, and methyl isothiocyanate, where we expected an insertion reaction into the remaining pentafulvene moiety or the Ti–N bond. Being a formal 18-electron complex,^30^**Ti1a** is highly restricted in its reactivity. Another interesting feature is the orientation of the C_exo_H in the solid state toward the nitrogen atoms. This was observed in every structure of this type with bond lengths of 2.36–2.54 Å (Table 1), which can be described as weak, noncovalent hydrogen bonds.^31^

**Table 1:** Selected Bond Parameters [Bond Lengths (Å) and Angles (deg)] of Complexes **Ti1a**, **Ti1b**, **Ti1c**, **Ti1d**, and **Ti1f**

complex	Ti1–N1	Ti1–N2	N1–N2	Ti1–C6	N1/N2–H	Ti1–N1–N2	Ti1–C6–C1
**Ti1a**	2.0999(6)	2.0714(6)	1.3409(8)	2.5077(7)	2.408	70.10(4)	60.05(4)
**Ti1b**	2.1321(10)	2.0763(10)	1.3388(14)	2.550	2.407	69.20(6)	59.03(6)
**Ti1c**	2.1205(11)	2.0662(11)	1.3285(15)	2.520	2.358	69.29(7)	59.65(7)
**Ti1d**	2.0795(8)	2.1159(8)	1.3603(11)	2.5445(9)	2.397	72.54(5)	58.91(5)
**Ti1f**	2.1421(6)	2.0880(6)	1.3661(9)	2.593	2.542	69.02(4)	57.54(4)

Contrary to initial thoughts, the thiophene carbaldehyde phenyl hydrazone **b** does not provide additional coordination via the S atom. The pentafluorobenzaldehyde phenylhydrazone **c** was used to raise the electrophilicity of the aldimine carbon atom to initiate an insertion reaction of the C=N double bond into the Ti–C_exo_ bond,^32^ however, this was not the case. None of the **Ti1a**-**f** and **Ti2a**,**b**,**d**,**e** could be converted into the respective insertion products. Upon heating, the complexes showed no signs of reaction and decomposed at around 60 °C in C_6_D_6_.

**Figure 3:**
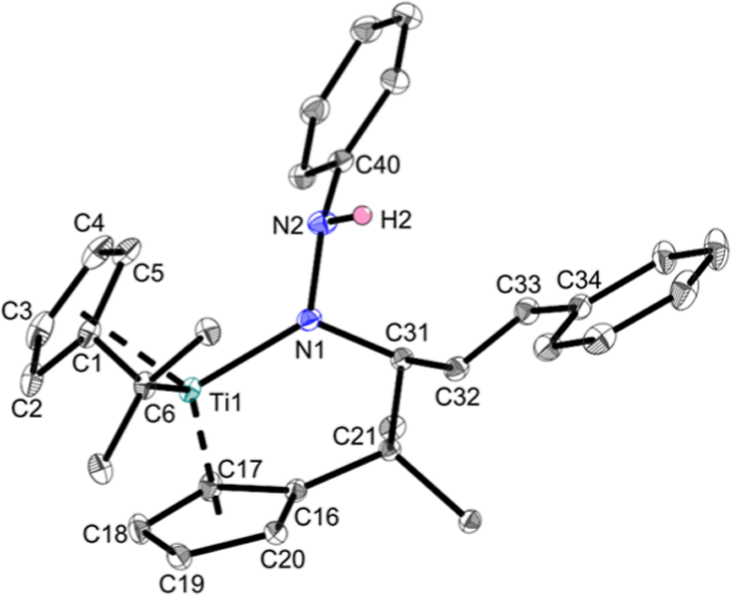
Molecular structures of complex **Ti1g**. Displacement ellipsoids are drawn at the 50% probability level. Redundant H atoms and adamantylidene rests have been omitted for clarity. Selected bond lengths (Å) and angles (deg): Ti1–N1 1.9936(11), N1–N2 1.4297(16), Ti1–C6 2.4238(13), N1–C31 1.4967(16), N2–C40 1.4054(18), C1–C6 1.4383(18), C21–C31 1.5655(17), C16–C21 1.5177(17), C31–C32 1.5083(17), C32–C33 1.3393(18), C33–C34 1.4686(18), N2–N1–Ti1 125.02(8), C40–N2–N1 117.49(10), N2–N1–C31 107.66(10), C16–C21–C31 106.00(9), N1–C31–C32 105.96(10), N1–C3–C21 110.82(9), and Ct1–Ti–Ct2 137.202 (Ct1 = centroid of C1–C5 and Ct2 = centroid of C16–C20).

The remaining hydrazones **g**, **h**, and **i** react differently. Hydrazone **g** reacts with the bis(π–η^5^:σ–η^1^-pentafulvene)titanium complexes to form product mixtures, as is evident from the ^1^H NMR spectrum (Supporting Information, Figure S13). One of the products was crystallized, and the molecular structure was revealed as a κ^1^*N* hydrazido complex via single-crystal X-ray diffraction (Figure 3), which was formed via the insertion of the C=N double bond of the hydrazone into the Ti–C_exo_ bond, thus forming the hydrazido ligand directly into the coordination sphere of the metal (Scheme 4). Insertion reactions of multiple bond substrates are usually encountered within the (pentafulvene)titanium complex chemistry.^20^

**Scheme 3:**
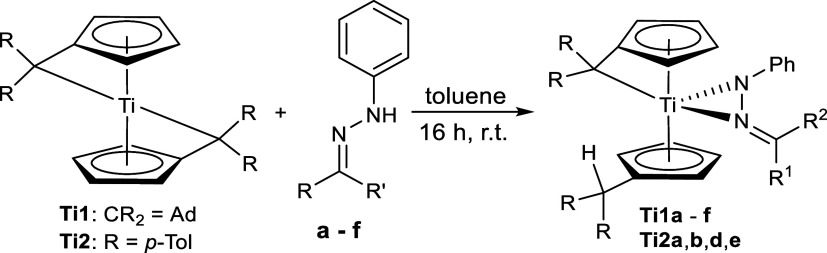
Reaction of Bis(π–η^5^:σ–η^1^-pentafulvene)titanium Complexes **Ti1** and **Ti2** with Phenylhydrazones **a**–**f** to κ^2^*N*,*N*-Hydrazonido Titanium Complexes **Ti1a-f** and **Ti2a,b,d,e**

**Scheme 4:**
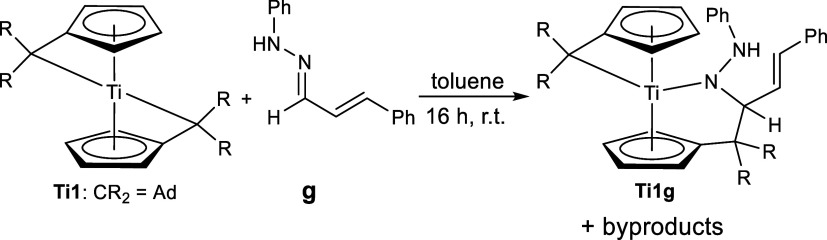
Reaction of Bis(π–η^5^:σ–η^1^-pentafulvene)titanium Complex **Ti1** with Phenylhydrazone **g** to Insertion Product **Ti1g** and Byproducts

The Ti1–N1 bond is best described as a single bond according to the sum of covalent radii [Σ*r*_cov_(Ti–N) = 2.07 Å]^24^ and the bond length of 1.9936(11) Å is similar to other κ^1^*N* hydrazido titanium complexes [1.977(7) Å, 1.952(12) Å].^25^ The Ti–C_exo_ bond of the remaining fulvene moiety [Ti1–C6 2.4238(13) Å] is shorter than that of the side-on κ^2^*N*,*N* hydrazonido complexes but longer than the Ti–C_exo_ bonds of **Ti1** [2.341(2) and 2.363(2) Å],^29^ which alludes to a higher reactivity of the pentafulvene moiety compared with **Ti1a** and derivatives. The newly formed C21–C31 bond [1.5655(17) Å] is comparable to a R_3_C(sp^3^)–C(sp^3^)R_2_H bond (1.556 Å)^39^ and the N–N bond with 1.4297(16) Å is unequivocally a single bond [Σ*r*_cov_(N–N) = 1.42 Å].^24^ This complex was identified in the ^1^H NMR spectrum of the product mixture via the identification of the N–H function in the ^1^H,^15^N-HMBC NMR spectrum, which shows the N–H nitrogen core at 131.0 ppm as a doublet because of coupling with the adjacent proton. The respective signals were then identified in the ^1^H NMR spectrum by integrating the signals. This shows that complex **Ti1g** is the main product of this reaction and is accompanied by at least two additional products. The number of products could be reduced neither through variation of the reaction conditions nor through crystallization, which is why **Ti1g** was not further analyzed.

**Scheme 5:**
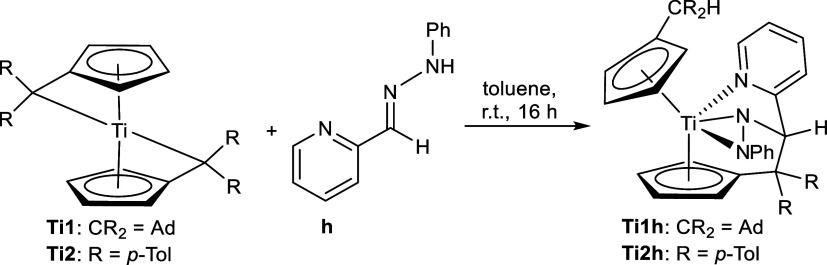
Reaction of Bis(π–η^5^:σ–η^1^-pentafulvene)titanium Complexes **Ti1** and **Ti2** with Phenylhydrazone **h** to Insertion Products **Ti1h,2h**

The reaction of hydrazone **h** with the bis(π–η^5^:σ–η^1^-pentafulvene)titanium complexes leads to κ^3^*N*,*N*,*N*-complexes via consecutive N–H activation and insertion (Scheme 5). The C–C coupling was rather unexpected because of the nucleophilic nature of both the aldimine carbon atom of the phenyl hydrazonido anion^33,34^ and the C_exo_ atom of the fulvene moiety.^35^ C–C couplings with phenyl hydrazones are known, but the reaction occurs with electrophiles rather than nucleophiles.^33,34,36−38^

**Figure 4:**
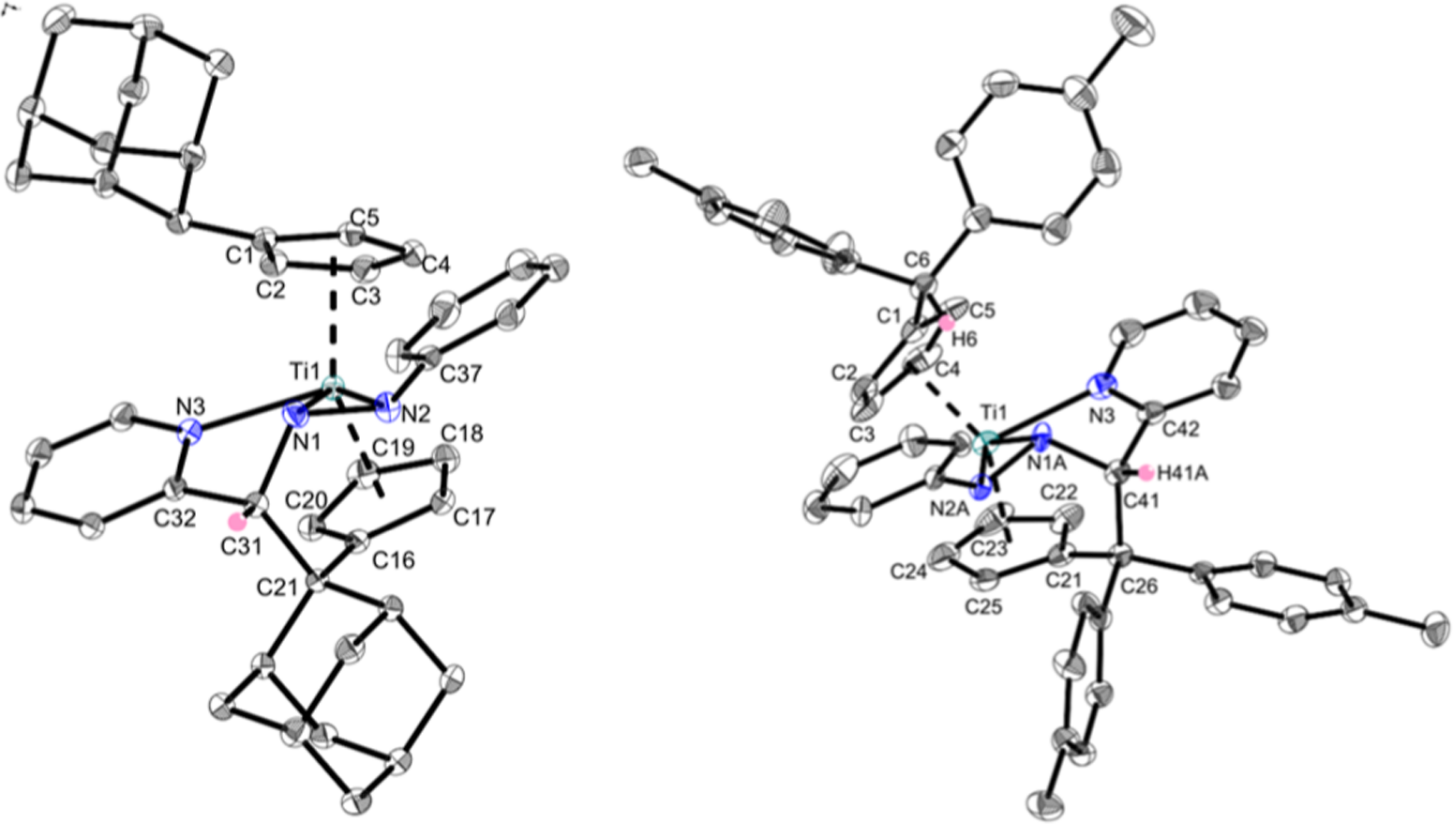
Molecular structures of complexes **Ti1h** and **Ti2h**. Displacement ellipsoids are drawn at the 50% probability level. Redundant H atoms have been omitted for clarity. Selected bond lengths (Å) and angles (deg): **Ti1h**, Ti1–N1 2.0629(16), Ti1–N2 2.0796(16), Ti1–N3 2.2658(15), N1–N2 1.388(2), N1–C31 1.488(2), C31–C21 1.591(2), C21–C16 1.526(2), N(2)–C(37) 1.375(2), N(2)–N(1)–Ti(1) 71.07(9), N2–Ti1–N1 39.15(6), N1–C31–C21 116.22(14), C16–C21–C31 105.41(13), and Ct1–Ti–Ct2 134.19; **Ti2h**, Ti1–N1A 2.067(6), Ti1–N2A 2.089(3), Ti1–N3 2.275(2), C41–N1A 1.481(6), N3–C42 1.348(3), C41–C42 1.506(3), C26–C41 1.594(3), C21–C26 1.515(3), N1A–Ti1–N3 69.32(13), N1A–Ti1–N2A 38.88(14), N2A–Ti1–N3 107.21(10), and Ct1–Ti–Ct2 132.97 (Ct1 = centroid of C1–C5 and Ct2 = centroid of C16–C20/C21–C25).

This was confirmed by single-crystal X-ray diffraction as the molecular structures of **Ti1h** and **Ti2h** reveal complexes with dianionic hydrazido ligands with additional coordination of the pyridine nitrogen (Figure 4).

The two very balanced Ti1–N1/N2 bonds [**Ti1h**: 2.0629(16) Å, 2.0796(16) Å; **Ti2h**: 2.067(6) Å, 2.089(3) Å] are best described as single bonds according to the covalent radii [Σ*r*_cov_(Ti–N) = 2.07 Å], whereas the longer Ti1–N3 bonds [2.2658(15) Å (**Ti1h**) and 2.275(2) Å (**Ti2h**)] resemble a dative bond. The bond lengths are comparable to those of other complexes that contain pyridine derivatives.^40^ The newly formed C21–C31/C26–C41 distances [1.591(2) Å (**Ti1h**), 1.594(3) Å (**Ti2h**)] are comparable to a regular R_3_C(sp^3^)–C(sp^3^)R_3_ bond (1.588 Å).^39^ The former C=N double bond of the hydrazone N1–C31/N1A–C41 [1.488(2) Å (**Ti1h**), 1.481(6) Å (**Ti2h**)] is now a single bond [Σ*r*_cov_(C–N) = 1.46 Å,^24^ Σr_cov_(C=N) = 1.27 Å],^28^ while the N–N bonds have more single bond character than **Ti1a-d,f** [Σ*r*_cov_(N–N) = 1.42 Å].^24^

In the ^1^H NMR spectrum, this insertion can be identified via the former aldimine signal, which can now be found more shielded at 6.10 ppm as a R_3_CH type of proton instead of the chemical shift of an aldimine (**Ti1a-c**, **Ti2a,b**) between 7.65 and 8.03 ppm. This is supported by the ^13^C{^1^H} NMR, with a corresponding value of 86.2 ppm instead of an aldimine signal in the range of 114.5–127.3 ppm. Although the signal for the pyridine nitrogen atom is missing in the ^1^H,^15^N-HMBC NMR spectrum, the other two singlets [222.7 ppm (N–Ph), 289.0 ppm (N–CH)] indicate that deprotonation occurred, as no doublet is found as for **Ti1g**.

**Scheme 6:**
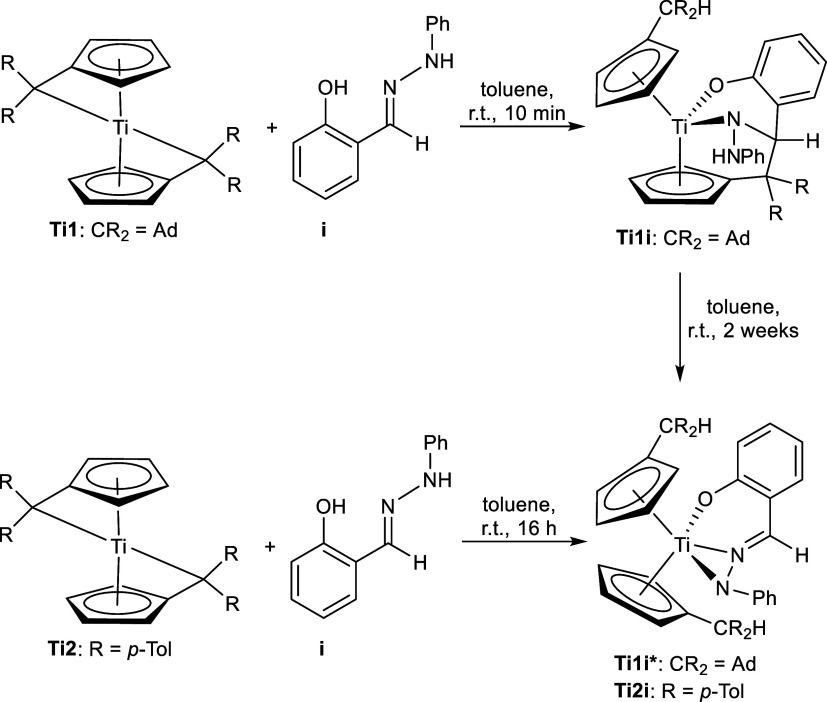
Reaction of Bis(π–η^5^:σ–η^1^-pentafulvene)titanium Complexes **Ti1** and **Ti2** with Phenylhydrazone **i** Fast reaction of **Ti1** to **Ti1i** and slow conversion to Ti1i*. **Ti2** reacts directly to **Ti2i**.

Hydrazone **i** reacts differently as the more acidic proton of the OH group is deprotonated first before the NH function, accompanied by an insertion of the C=N bond into the Ti–C_exo_ bond (Scheme 6, top).

**Figure 5:**
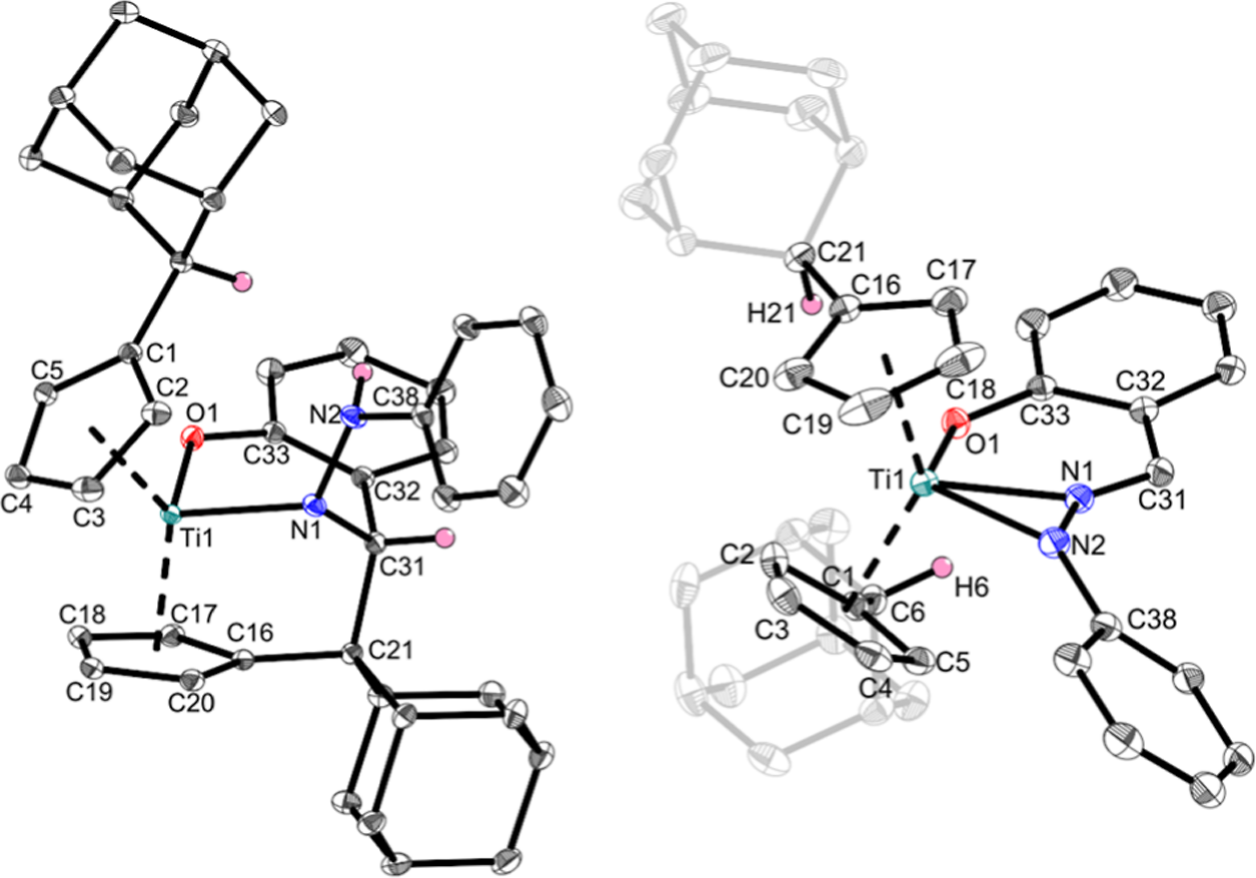
Molecular structures of complexes **Ti1i** (left) and Ti1i* (right). Displacement ellipsoids are drawn at the 50% probability level. Redundant H atoms, solvent molecules, and parts of adamantylidene rests have been omitted for clarity. Selected bond lengths (Å) and angles (deg): **Ti1i**, Ti1–N1 2.0073(6), Ti1–O1 1.9332(6), N1–N2 1.4147(9), N1–C31 1.4894(10), N2–C38 1.3914(10), O1–C33 1.3396(9), C33–C32 1.4204(10), C32–C31 1.5339(10), C31–C21 1.5747(10), O1–H6 2.712, N2–H6 2.588, O1–Ti1–N1 88.33(3), and Ct1–Ti1–Ct2 132.1; Ti1i*, Ti1–N1 2.0016(8), Ti1–N2 2.1485(8), Ti1–O1 2.0221(7), N1–N2 1.3283(11), O1–C33 1.3197(11), N1–C31 1.2904(12), N2–C38 1.3888(12), C31–C32 1.4471(13), C32–C33 1.4285(13), O1–H6 2.418, O1–H21 2.419, Ti1–N1–N2 77.46(5), Ti1–N1–C31 149.50(7), O1–Ti1–N1 79.38(3), and Ct1–Ti1–Ct2 132.0 (Ct1 = centroid of C1–C5 and Ct2 = centroid of C16–C20).

This coordination of complex **Ti1i** was confirmed by NMR spectroscopy (Supporting Information, Figure S18) and single-crystal X-ray diffraction (Figure 5, left). Its molecular structure reveals a shortened Ti–N bond (2.0073(6) Å) compared with previous structures and the sum of covalent radii [Σ*r*_cov_(Ti–N) = 2.07 Å].^24^ The Ti–O bond [1.9332(6) Å] corresponds to a shortened single bond according to the sum of covalent radii [Σ*r*_cov_(Ti–O) = 1.99 Å].^24^ The N–N bond similar to **Ti1g** with 1.4147(9) Å is unequivocally a single bond [Σ*r*_cov_(N–N) = 1.42 Å]^24^ and the newly formed C21–C31 bond [1.5747(10) Å] is comparable to a R_3_C(sp^3^)–C(sp^3^)R_2_H bond (1.556 Å).^39^ The ^1^H NMR spectrum reveals eight different Cp–H signals, corresponding to asymmetrical pentafulvene units. The former aldimine signal is found at 5.01 ppm, which is considerably more shielded compared with the observed aldimine shifts between 7.65 and 8.03 ppm. The ^1^H,^15^N-HMBC NMR spectrum shows a doublet at 142.2 ppm, corresponding to the N–H function. The additional signals found in the ^1^H NMR spectrum indicate the formation of an additional product. By heating the sample over a span of 2 weeks at 60 °C, the slow conversion of **Ti1i** to Ti1i* was observed (Scheme 6, right). The slow conversion could not be detected for **Ti2i**, which led directly to the double-deprotonated product (Scheme 6, bottom). The formation of Ti1i* indicates that the OH group was deprotonated by the second pentafulvene unit, which is possible only by C–C splitting of the newly formed C–C bond of the insertion product. This is further reinforced by the ^1^H NMR spectrum of Ti1i*, which shows four different Cp–H signals, indicating that both Cp groups are equivalent. The C–C splitting is rather atypical in the context of insertion products of (pentafulvene)titanium complexes. We presume that this reaction occurred because of the superior thermodynamic stability of the 18-electron complex Ti1i* as opposed to that of the 16-electron complex **Ti1i**. The molecular structure of Ti1i* was confirmed by single-crystal X-ray diffraction (Figure 5, right).

**Scheme 7:**
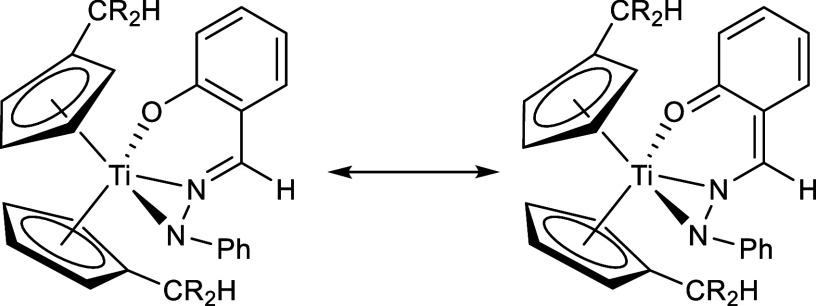
Proposed Main Resonance Structures of Ti1i*

The molecular structure of Ti1i* displays a planar, six-membered titanacycle with additional coordination via the N2 atom. The Ti1–N1 bond [2.0016(8) Å] is again a shortened single bond, whereas the formal covalent Ti1–N2 bond [2.1485(8) Å] is counterintuitively enlarged compared to the sum of covalent radii [Σ*r*_cov_(Ti–N) = 2.07 Å] and corresponds more to an elongated bond. The Ti–O bond [2.0221(7) Å] is longer than that of **Ti1i** and corresponds to an enlarged bond. This bonding situation can be explained by the proposed resonance structure of the ligand (Scheme 7).

This is underpinned by the smaller O1–C31 bond [1.3197(11) Å] of Ti1i* compared with **Ti1i** [1.3396(9) Å], showing a higher double bond character [Σ*r*_cov_(C–O) = 1.38 Å,^24^ Σ*r*_cov_(C=O) = 1.24 Å].^28^ Additionally, the C37–C36 bond [1.4471(13) Å] of Ti1i* shows considerable double bond character, whereas the same bond in **Ti1i** [1.5339(10) Å] is clearly a single bond.

## Summary and Conclusions

In this work, we demonstrate the multifaceted coordination chemistry of hydrazones in the coordination sphere of titanium. Without additional functional groups, κ^2^*N*,*N* side-on coordination of the hydrazonido ligand occurred by the deprotonation of the N–H bond. In the case of cinnamon aldehyde phenylhydrazone, we observed an insertion reaction leading to a κ^1^*N* hydrazido titanium complex. While the thiophene rest was not sufficient for additional coordination, the introduction of a pyridine rest was responsible for a consecutive insertion and deprotonation reaction, which revealed a κ^3^*N*,*N*,*N* coordination mode. The use of salicylaldehyde phenylhydrazone ultimately resulted in κ^3^*N*,*N*,*O* coordination via double deprotonation of the ligand. The κ^2^*N*,*O*-coordinating intermediate was isolated and comprehensively analyzed by NMR spectroscopy and single-crystal X-ray diffraction.

## Experimental Section

All reactions were carried out under a dry nitrogen or argon atmosphere using standard Schlenk and glovebox techniques. Caution! Extreme care should be taken both in the handling of cryogen liquid nitrogen and its use in the Schlenk line trap to avoid the condensation of oxygen from air. Solvents were dried according to standard procedures over a Na/K alloy with benzophenone as an indicator, subsequently distilled, and stored under a nitrogen atmosphere. NMR spectra were recorded on a Bruker AVANCE III 500 spectrometer (^1^H 500 MHz). IR spectra were recorded on a Bruker Tensor 27 spectrometer using an attenuated total reflection (ATR) method. Elemental analyses were carried out on a Euro EA 3000 Elemental Analyzer. Melting points were determined using a “Mel-Temp” from Laboratory Devices, Cambridge, or a Mettler Toledo MP30. Further exact details of syntheses, crystallographic data, and NMR spectra are given in the Supporting Information.

## Supporting Information Available

NMR spectra and crystallographic details (PDF)

## References

[ref1] Brady O. L.; Elsmie G. V. The use of 2:4-dinitrophenylhydrazine as a reagent for aldehydes and ketones. Analyst 1926, 51 (599), 77 10.1039/an9265100077.

[ref2] Xu H.; Yang P.; Chuanprasit P.; Hirao H.; Zhou J. S. Nickel-catalyzed asymmetric transfer hydrogenation of hydrazones and other ketimines. Angew. Chem., Int. Ed. 2015, 54 (17), 5112–5116. 10.1002/anie.201501018. 25737093

[ref3] Li B.; Liu D.; Hu Y.; Chen J.; Zhang Z.; Zhang W. Nickel-Catalyzed Asymmetric Hydrogenation of Hydrazones. Eur. J. Org Chem. 2021, 2021 (23), 3421–3425. 10.1002/ejoc.202100642.

[ref4] Schuster C. H.; Dropinski J. F.; Shevlin M.; Li H.; Chen S. Ruthenium-Catalyzed Enantioselective Hydrogenation of Hydrazones. Org. Lett. 2020, 22 (19), 7562–7566. 10.1021/acs.orglett.0c02756. 32946691

[ref5] Cheng L.; Li M.-M.; Wang B.; Xiao L.-J.; Xie J.-H.; Zhou Q.-L. Nickel-catalyzed hydroalkylation and hydroalkenylation of 1,3-dienes with hydrazones. Chem. Sci. 2019, 10 (44), 10417–10421. 10.1039/C9SC04177J. 32110333 PMC6988744

[ref6] Lv L.; Zhu D.; Qiu Z.; Li J.; Li C.-J. Nickel-Catalyzed Regioselective Hydrobenzylation of 1,3-Dienes with Hydrazones. ACS Catal. 2019, 9 (10), 9199–9205. 10.1021/acscatal.9b02483.

[ref7] Yu L.; Lv L.; Qiu Z.; Chen Z.; Tan Z.; Liang Y.-F.; Li C.-J. Palladium-Catalyzed Formal Hydroalkylation of Aryl-Substituted Alkynes with Hydrazones. Angew. Chem. 2020, 132 (33), 14113–14117. 10.1002/ange.202005132. 32365254

[ref8] Li X.; He L.; Chen H.; Wu W.; Jiang H. Copper-catalyzed aerobic C(sp2)-H functionalization for C-N bond formation: synthesis of pyrazoles and indazoles. J. Org. Chem. 2013, 78 (8), 3636–3646. 10.1021/jo400162d. 23547954

[ref9] Chuang S.-C.; Gandeepan P.; Cheng C.-H. Synthesis of isoquinolines via Rh(III)-catalyzed C-H activation using hydrazone as a new oxidizing directing group. Org. Lett. 2013, 15 (22), 5750–5753. 10.1021/ol402796m. 24156564

[ref10] Pouralimardan O.; Chamayou A.-C.; Janiak C.; Hosseini-Monfared H. Hydrazone Schiff base-manganese(II) complexes: Synthesis, crystal structure and catalytic reactivity. Inorg. Chim. Acta 2007, 360 (5), 1599–1608. 10.1016/j.ica.2006.08.056.

[ref11] Adejumo T. T.; Tzouras N. V.; Zorba L. P.; Radanović D.; Pevec A.; Grubišić S.; Mitić D.; And̵elković K. K.; Vougioukalakis G. C.; Čobeljić B.; Turel I. Synthesis, Characterization, Catalytic Activity, and DFT Calculations of Zn(II) Hydrazone Complexes. Molecules 2020, 25 (18), 4043 10.3390/molecules25184043. 32899683 PMC7570652

[ref12] Salem A. A. Spectrophotometric and Potentiometric Characterization of Some Arylhydrazone Derivatives and their Applications in Lanthanide Determination. Microchem. J. 1998, 60 (1), 51–66. 10.1006/mchj.1998.1621.

[ref13] Sivaramaiah S.; Raveendra Reddy P. Direct and Derivative Spectrophotometric Determination of Zinc with 2,4-Dihydroxybenzaldehyde Isonicotinoyl Hydrazone in Potable Water and Pharmaceutical Samples. J. Anal. Chem. 2005, 60 (9), 828–832. 10.1007/s10809-005-0190-y.

[ref14] Badiger D. S.; Hunoor R. S.; Patil B. R.; Vadavi R. S.; Mangannavar C. V.; Muchchandi I. S.; Patil Y. P.; Nethaji M.; Gudasi K. B. Synthesis, spectroscopic properties and biological evaluation of transition metal complexes of salicylhydrazone of anthranilhydrazide: X-ray crystal structure of copper complex. Inorg. Chim. Acta 2012, 384, 197–203. 10.1016/j.ica.2011.11.063.

[ref15] Hong M.; Yin H.; Zhang X.; Li C.; Yue C.; Cheng S. Di- and tri-organotin(IV) complexes with 2-hydroxy-1-naphthaldehyde 5-chloro-2-hydroxybenzoylhydrazone: Synthesis, characterization and in vitro antitumor activities. J. Org. Chem. 2013, 724, 23–31. 10.1016/j.jorganchem.2012.10.031.

[ref16] Dilworth J. R. Diazene, diazenido, isodiazene and hydrazido complexes. Coord. Chem. Rev. 2017, 330, 53–94. 10.1016/j.ccr.2016.09.006.

[ref17] Uhl W.; Molter J.; Neumüller B. The hydrogallation of C N double bonds—reactions of GaH_3_·NMe_2_Et with tetramethyl-2,3-diazabutadiene. J. Organomet. Chem. 2001, 634 (2), 193–197. 10.1016/S0022-328X(01)01158-5.

[ref18] Han Y.; Zhang J.; Han F.; Zhang Z.; Weng L.; Zhou X. Investigations on Organolanthanide Derivatives with the Hydrazonido (-NHN=CPh_2_) Ligand: Synthesis, Crystal Structure, and Reactivity. Organometallics 2009, 28 (13), 3916–3921. 10.1021/om900246b.

[ref19] Fitschen K.; Schmidtmann M.; Beckhaus R. CCDC 2299395: Experimental Crystal Structure Determination, 2023, 10.5517/ccdc.csd.cc2h5q1g.

[ref20] Beckhaus R. Pentafulvene complexes of group four metals: Versatile organometallic building blocks. Coord. Chem. Rev. 2018, 376, 467–477. 10.1016/j.ccr.2018.08.020.

[ref21] Manßen M.; Lauterbach N.; Dörfler J.; Schmidtmann M.; Saak W.; Doye S.; Beckhaus R. Efficient access to titanaaziridines by C-H activation of N-methylanilines at ambient temperature. Angew. Chem., Int. Ed. 2015, 54 (14), 4383–4387. 10.1002/anie.201500796. 25783181

[ref22] Harej M.; Dolenc D. Autoxidation of hydrazones. Some new insights. J. Org. Chem. 2007, 72 (19), 7214–7221. 10.1021/jo071091m. 17696476

[ref23] Petunin P. V.; Martynko E. A.; Trusova M. E.; Kazantsev M. S.; Rybalova T. V.; Valiev R. R.; Uvarov M. N.; Mostovich E. A.; Postnikov P. S. Verdazyl Radical Building Blocks: Synthesis, Structure, and Sonogashira Cross-Coupling Reactions. Eur. J. Org Chem. 2018, 2018 (34), 4802–4811. 10.1002/ejoc.201701783.

[ref24] Pyykkö P.; Atsumi M. Molecular single-bond covalent radii for elements 1–118. Chem 2009, 15 (1), 186–197. 10.1002/chem.200800987. 19058281

[ref25] Park J. T.; Yoon S. C.; Bae B.-J.; Seo W. S.; Suh I.-H.; Han T. K.; Park J. R. Cyclopentadienyl-Hydrazido Titanium Complexes: Synthesis, Structure, Reactivity, and Catalytic Properties. Organometallics 2000, 19 (7), 1269–1276. 10.1021/om990841i.

[ref26] Evans W. J.; Kociok-Koehn G.; Leong V. S.; Ziller J. W. Reactivity of hydrazines with organometallic samarium complexes and the x-ray crystal structures of (C_5_Me_5_)_2_Sm(η^2^-PhNHNPh)(THF), (C_5_Me_5_)_2_Sm(NHPh)(THF), and [(C5Me5)2Sm]2(η^2^: η^2^-HNNH). Inorg. Chem. 1992, 31 (17), 3592–3600. 10.1021/ic00043a020.

[ref27] Baunemann A.; Kim Y.; Winter M.; Fischer R. A. Mixed hydrazido amido/imido complexes of tantalum, hafnium and zirconium: potential precursors for metal nitride MOCVD. Dalton Trans. 2006, (1), 121–128. 10.1039/B512074H. 16357967

[ref28] Pyykkö P.; Atsumi M. Molecular double-bond covalent radii for elements Li-E112. Chem 2009, 15 (46), 12770–12779. 10.1002/chem.200901472. 19856342

[ref29] Diekmann M.; Bockstiegel G.; Lützen A.; Friedemann M.; Saak W.; Haase D.; Beckhaus R. Chiral Bis(η^5^:η^1^ -pentafulvene)titanium Complexes. Organometallics 2006, 25 (2), 339–348. 10.1021/om050815m.

[ref30] Tolman C. A. The 16 and 18 electron rule in organometallic chemistry and homogeneous catalysis. Chem. Soc. Rev. 1972, 1 (3), 337 10.1039/cs9720100337.

[ref31] Szatyłowicz H. Structural aspects of the intermolecular hydrogen bond strength: H-bonded complexes of aniline, phenol and pyridine derivatives. J. Phys. Org. Chem. 2008, 21 (10), 897–914. 10.1002/poc.1394.

[ref32] Pažitný A.; Solčán T.; Végh D. Pentafluorobenzaldehyde and its utilizing in organic synthesis. J. Fluor. Chem. 2009, 130 (3), 267–294. 10.1016/j.jfluchem.2008.12.013.

[ref33] Baldwin J. E.; Adlington R. M.; Bottaro J. C.; Kolhe J. N.; Perry M. W.; Jain A. U. Azo anions in systhesis. pt 1. t-butylhydrazones as acyl-anion equivalents. Tetrahedron 1986, 42 (15), 4223–4234. 10.1016/S0040-4020(01)87647-X.

[ref34] Baldwin J. E.; Adlington R. M.; Jain A. U.; Kolhe J. N.; Perry M. W. Applications of the thermal ene reaction of aldehyde -butyl- and phenyl- hydrazones. Tetrahedron 1986, 42 (15), 4247–4252. 10.1016/S0040-4020(01)87649-3.

[ref35] Janssen T.; Severin R.; Diekmann M.; Friedemann M.; Haase D.; Saak W.; Doye S.; Beckhaus R. Bis(η^5^:η^1^ -pentafulvene)titanium Complexes: Catalysts for Intramolecular Alkene Hydroamination and Reagents for Selective Reactions with N-H Acidic Substrates. Organometallics 2010, 29 (7), 1806–1817. 10.1021/om100056q.

[ref36] El Kaïm L.; Gautier L.; Grimaud L.; Michaut V. New Insight into the Azaenamine Behaviour of N -Arylhydrazones: First Aldol and Improved Mannich Reactions with Unactivated Aldehydes. Synlett 2003, (12), 1844–1846. 10.1055/s-2003-41441.

[ref37] Baldwin J. E.; Adlington R. M.; Bottaro J. C.; Kolhe J. N.; Newington I. M.; Perry M. W. Azo anions in synthesis. Tetrahedron 1986, 42 (15), 4235–4246. 10.1016/S0040-4020(01)87648-1.

[ref38] Brehme R.; Enders D.; Fernandez R.; Lassaletta J. M. Aldehyde N, N -Dialkylhydrazones as Neutral Acyl Anion Equivalents: Umpolung of the Imine Reactivity. Eur. J. Org Chem. 2007, 2007 (34), 5629–5660. 10.1002/ejoc.200700746.

[ref39] Allen F. H.; Kennard O.; Watson D. G.; Brammer L.; Orpen A. G.; Taylor R. Tables of bond lengths determined by X-ray and neutron diffraction. Part 1. Bond lengths in organic compounds. J. Chem. Soc., Perkin Trans. 2 1987, (12), S1 10.1039/p298700000s1.

[ref40] Annunziata L.; Pragliola S.; Pappalardo D.; Tedesco C.; Pellecchia C. New (Anilidomethyl)pyridine Titanium(IV) and Zirconium(IV) Catalyst Precursors for the Highly Chemo- and Stereoselective cis −1,4-Polymerization of 1,3-Butadiene. Macromolecules 2011, 44 (7), 1934–1941. 10.1021/ma1028455.

