# Supplementary material for: Syntheses, Characterization, and Multifaceted Coordination Chemistry of Hydrazonido Titanium Complexes
Source: Inorg Chem. 2024 Jan 27;63(6):3165–72. doi: 10.1021/acs.inorgchem.3c04301 (PMC10865377; doi:10.1021/acs.inorgchem.3c04301)
Supplement: Supplementary file 1 — ic3c04301_si_001.pdf [file ic3c04301_si_001.pdf]

# Supporting Information

## Syntheses, Characterization and Multifaceted Coordination Chemistry of Hydrazonido Titanium complexes

Kevin Schwitalla, Fares Sad, Marc Schmidtmann and Rüdiger Beckhaus*

Chemistry Department, Carl von Ossietzky University of Oldenburg, 26111 Oldenburg, Germany

E-mail: ruediger.beckhaus@uol.de

## TABLE OF CONTENTS

**Synthesis and characterization of compounds**....... 2

**NMR spectra of complexes**....... 17

**Crystallographic data**....... 32

**Molecular structures**....... 35

**References**....... 40

## Synthesis and characterization of compounds

## Synthesis of Ti1a:

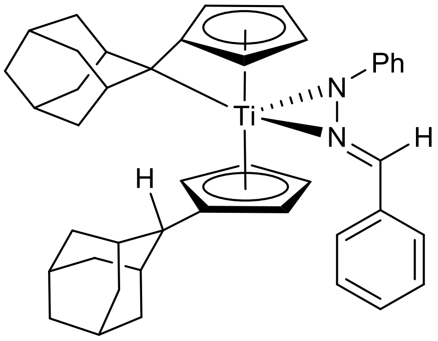


Bis(adamantylidenefulvene)titanium complex **Ti1** (200 mg, 0.450 mmol) and benzaldehyde phenylhydrazone **a** (88.1 mg, 0.450 mmol) were dissolved in 10 ml of dry toluene. The reaction mixture was stirred for 16 h at room temperature to give a dark brown solution. The solvent was removed under reduced pressure and the residue was washed with 10 mL of *n*-hexane. All volatile components were removed under reduced pressure and the residue was dried under vacuum to yield the product as a black brown solid. Red orange crystals suitable for single crystal X-ray diffraction analysis precipitated from a slowly evaporating solution of **Ti1a** in $C_6D_6$ after several days.

**Yield**: 0.232 g, 0.362 mmol, 80%.

**$^1$H NMR** ($C_6D_6$, 500 MHz, 305 K): δ = 1.29-2.38 (m, 29 H, Ad-H), 4.25-4.30 (m, 1 H, Cp-H), 4.31-4.35 (m, 1 H, Cp-H), 4.79-4.84 (m, 1 H, Cp-H), 5.22-5.26 (m, 1 H, Cp-H), 5.33-5.38 (m, 1 H, Cp-H), 5.77-5.80 (m, 1 H, Cp-H), 5.95-6.00 (m, 1 H, Cp-H), 6.52-6.57 (m, 1 H, Cp-H), 6.83-6.89 (m, 3 H, Ph-H), 7.00-7.04 (m, 1 H, Ph-H), 7.17-7.21 (m, 4 H, Ph-H), 7.33-7.37 (m, 2 H, Ph-H), 7.80 (s, 1 H, aldimine-H) ppm.

**$^{13}$C{$^1$H} NMR** ($C_6D_6$, 125 MHz, 305 K): δ = 28.4 (2 x Ad-CH), 28.9 (Ad-CH), 29.8 (Ad-CH), 31.9 (Ad-CH), 32.9 (Ad-$CH_2$), 33.0 (Ad-CH), 33.1 (Ad-$CH_2$), 34.5 (Ad-CH), 36.8 (Ad-CH), 37.2 (Ad-$CH_2$), 38.3 (Ad-$CH_2$), 38.3 (Ad-$CH_2$), 38.8 (Ad-$CH_2$), 39.2 (Ad-$CH_2$), 39.4 (Ad-$CH_2$), 44.2 (Ad-CH), 44.4 (Ad-$CH_2$), 45.7 (Ad-$CH_2$), 102.0 (Cp-CH), 102.4 (Cp-CH), 103.0 (Cp-CH), 103.2 (Cp-CH), 103.5 (Cp-CH), 108.1 (Cp-CH), 113.2 (Ph-CH), 119.5 (Cp-CH), 120.8 ($C_{exo}$-$C_q$), 120.8 ($C_{ipso}$-$C_q$), 123.0 (Ph-CH), 123.3(Ph-CH), 124.8 (Ph-CH), 126.6 (Ph-CH), 127.2 (aldimine-C), 128.4 (Ph-CH), 128.7 (Ph-CH), 129.3 (Ph-CH), 131.8 ($C_{ipso}$-$C_q$), 136.2 (Ph-$C_q$), 148.4 (Ph-$C_q$) ppm.

**$^{15}$N NMR** ($C_6D_6$, 51 MHz, 305 K): δ = 179.1 (N-Ph), 287.1 (N=CHPh) ppm.

**IR** (ATR): ṽ = 2965, 2913, 2858, 1601, 1576, 1509, 1446, 1376, 1261, 1109, 1083, 1053, 1021, 840, 790, 762, 718 $cm^{-1}$.

**Melting point**: 166 – 168 °C.

**EA**: calcd. for $C_{43}H_{48}N_2Ti$: C 80.61, H 7.55, N 4.37. Found: C 81.03, H 8.02, N 4.39.

## Synthesis of Ti1b:

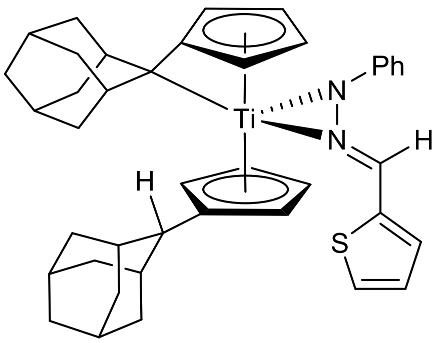


Bis(adamantylidenefulvene)titanium complex **Ti1** (200 mg, 0.450 mmol) and 2-thiophenecarboxaldehyde phenylhydrazone **b** (91.0 mg, 0.450 mmol) were dissolved in 10 ml of dry toluene. The reaction mixture was stirred for 16 h at room temperature to give a dark brown solution. The solvent was removed under reduced pressure and the residue was washed with 10 mL of *n*-hexane. All volatile components were removed under reduced pressure and the residue was dried under vacuum to yield the product as a brown solid. Orange crystals suitable for single crystal X-ray diffraction analysis precipitated from a slowly evaporating solution of **Ti1b** in $C_6D_6$ after several days.

**Yield**: 0.266 g, 0.411 mmol, 91%.

**$^1$H NMR** ($C_6D_6$, 500 MHz, 305 K): δ = 1.33-2.39 (m, 29 H, Ad-H, $C_{exo}$H), 4.19-4.22 (m, 1 H, Cp-H), 4.34-4.37 (m, 1 H, Cp-H), 4.78-4.81 (m, 1 H, Cp-H), 5.27-5.30 (m, 1 H, Cp-H), 5.42-5.46 (m, 1 H, Cp-H), 5.96-5.99 (m, 1 H, Cp-H), 6.15-6.18 (m, 1 H, Cp-H), 6.53-6.57 (m, 2 H, Cp-H, $C_4H_3S$-H), 6.70-6.73 (m, 1 H, $C_4H_3S$-H), 6.75-6.78 (m, 1 H, $C_4H_3S$-H), 6.80-6.86 (m, 3 H, Ph-H), 7.12-7.15 (m, 2 H, Ph-H), 8.03 (s, 1 H, aldimine-H) ppm.

**$^{13}$C{$^1$H} NMR** ($C_6D_6$, 125 MHz, 305 K): δ = 28.4 (2 x Ad-CH), 28.9 (Ad-CH), 29.8 (Ad-CH), 31.9 (Ad-CH), 32.9 (Ad-$CH_2$), 33.0 (Ad-CH), 33.1 (Ad-$CH_2$), 34.5 (Ad-CH), 36.8 (Ad-CH), 37.2 (Ad-$CH_2$), 38.3 (Ad-$CH_2$), 38.4 (Ad-$CH_2$), 39.0 (Ad-$CH_2$), 39.2 (Ad-$CH_2$), 39.5 (Ad-$CH_2$), 44.2 (Ad-CH), 44.4 (Ad-$CH_2$), 45.7 (Ad-$CH_2$), 101.8 (Cp-CH), 102.6 (Cp-CH), 103.0 (Cp-CH), 103.3 (Cp-CH), 103.9 (Cp-CH), 104.2 (Cp-CH), 107.4 (Cp-CH), 119.6 (Cp-CH), 120.8 ($C_{exo}$-$C_q$), 120.9 ($C_{ipso}$-$C_q$), 122.7 ($C_4H_3S$-CH), 123.2($C_4H_3S$-CH), 123.2(Ph-CH), 123.9 ($C_4H_3S$-CH), 124.0 (Ph-CH), 127.3 (aldimine-C), 128.4 (Ph-CH), 128.7 (Ph-CH), 129.4 (Ph-CH), 131.6 ($C_{ipso}$-$C_q$), 140.8 ($C_4H_3S$-$C_q$), 148.4 (Ph-$C_q$) ppm.

**$^{15}$N NMR** ($C_6D_6$, 51 MHz, 305 K): δ = 179.3 (N-Ph), 284.5 (N=CH($C_4H_3S$)) ppm.

**IR** (ATR): ṽ = 2900, 2847, 1587, 1540, 1505, 1479, 1444, 1363, 1258, 1238, 1096, 1061, 1043, 850, 807, 798, 777, 751, 721, 695, 683, 577 $cm^{-1}$.

**Melting point**: 145 – 150 °C.

**EA**: calcd. for $C_{41}H_{46}N_2STi$: C 76.14, H 7.17, N 4.33. Found: C 76.00, H 7.69, N 4.33.

## Synthesis of Ti1c:

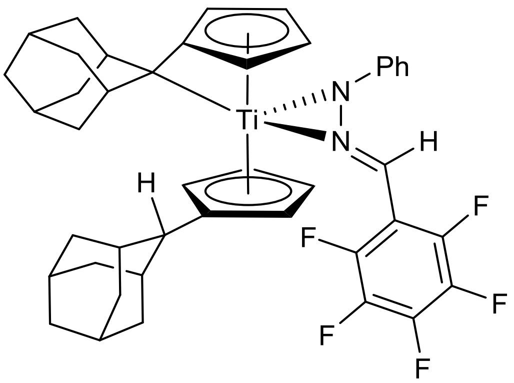


Bis(adamantylidenefulvene)titanium complex **Ti1** (300 mg, 0.675 mmol) and 2,3,4,5,6-pentafluorobenzaldehyde phenylhydrazone **c** (193 mg, 0.675 mmol) were dissolved in 10 ml of dry toluene. The reaction mixture was stirred for 16 h at room temperature to give a dark brown solution. The solvent was removed under reduced pressure and the residue was washed with 10 mL of *n*-hexane. All volatile components were removed under reduced pressure and the residue was dried under vacuum to yield the product as a black brown solid. Orange crystals suitable for single crystal X-ray diffraction analysis precipitated from a slowly evaporating solution of **Ti1c** in $C_6D_6$ after several days.

**Yield**: 0.382 g, 0.523 mmol, 78%.

**$^1$H NMR** ($C_6D_6$, 500 MHz, 305 K): δ = 1.35-2.34 (m, 29 H, Ad-H, $C_{exo}$H), 4.22-4.26 (m, 1 H, Cp-H), 4.39-4.43 (m, 1 H, Cp-H), 4.72-4.76 (m, 1 H, Cp-H), 5.24-5.29 (m, 1 H, Cp-H), 5.34-5.39 (m, 1 H, Cp-H), 5.73-5.77 (m, 1 H, Cp-H), 6.04-6.08 (m, 1 H, Cp-H), 6.52-6.56 (m, 1 H, Cp-H), 6.63-6.65 (m, 1 H, Ph-H), 6.79-6.90 (m, 4 H, Ph-H), 7.65 (s, 1 H, aldimine-H) ppm.

**$^{13}$C{$^1$H} NMR** ($C_6D_6$, 125 MHz, 305 K): δ = 28.3 (Ad-CH), 28.3 (Ad-CH), 28.7 (Ad-CH), 29.8 (Ad-CH), 32.2 (Ad-CH), 32.9 (2 x Ad-$CH_2$), 33.1 (Ad-CH), 34.7 (Ad-CH), 36.7 (Ad-CH), 37.0 (Ad-$CH_2$), 38.2 (Ad-$CH_2$), 38.2 (Ad-$CH_2$), 38.8 (Ad-$CH_2$), 39.0 (Ad-$CH_2$), 39.1 (Ad-$CH_2$), 44.1 (Ad-CH), 44.5 (Ad-$CH_2$), 45.8 (Ad-$CH_2$), 102.0 (Cp-CH), 103.5 (Cp-CH), 103.5 (Cp-CH), 104.3 (Cp-CH), 105.2 (Cp-CH), 108.8 (Cp-CH), 113.4 (Cp-CH), 114.5 (aldimine-C), 115.1 ($C_6F_5$-CF), 117.0 ($C_6F_5$-CF), 119.3 (Cp-CH), 119.9 ($C_6F_5$-CF), 120.4 ($C_{ipso}$-$C_q$), 122.6 ($C_6F_5$-CF), 123.5 (Ph-CH), 125.1 (Ph-CH), 128.4 (Ph-CH), 129.5 (2 x Ph-CH), 129.7 ($C_6F_5$-CF), 131.7 ($C_{ipso}$-$C_q$), 133.5 ($C_6F_5$-$C_q$), 146.2 (Ph-$C_q$) ppm.

**$^{15}$N NMR** ($C_6D_6$, 51 MHz, 305 K): δ = 200.5 (N-Ph), 304.3 (N=CH($C_6F_5$)) ppm.

**IR** (ATR): ṽ = 2900, 2848, 1641, 1601, 1576, 1515, 1486, 1449, 1420, 1363, 1312, 1261, 1170, 1098, 1062, 1030, 1015, 963, 884, 861, 796, 773, 755, 725, 692, 625, 607, 578 $cm^{-1}$.

**Melting point**: 106-110 °C.

**EA**: calcd. for $C_{43}H_{43}F_5N_2Ti$: C 70.68, H 5.93, N 3.83. Found: 69.58, H 6.23, N 3.42.

## Synthesis of Ti1d:

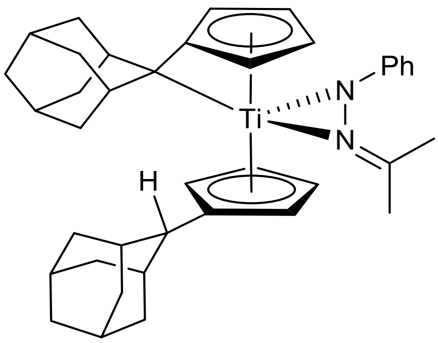


Bis(adamantylidenefulvene)titanium complex **Ti1** (200 mg, 0.450 mmol) and acetone phenylhydrazone **d** (66.7 mg, 0.450 mmol) were dissolved in 10 ml of dry toluene. The reaction mixture was stirred for 16 h at room temperature to give a dark brown solution. The solvent was removed under reduced pressure and the residue was washed with 10 mL of *n*-hexane. All volatile components were removed under reduced pressure and the residue was dried under vacuum to yield the product as a brown solid. Yellow crystals suitable for single crystal X-ray diffraction analysis precipitated from a slowly evaporating solution of **Ti1d** in $C_6D_6$ after several days.

**Yield**: 0.215 g, 0.363 mmol, 81%.

**$^1$H NMR** ($C_6D_6$, 500 MHz, 305 K): δ = 1.29-1.33 (m, 1 H, Ad-H), 1.42 (s, 3 H, $CH_3$), 1.53-1.56 (m, 1 H, Ad-H), 1.57 (s, 3 H, $CH_3$), 1.61-2.56 (m, 27 H, Ad-H), 3.75-3.79 (m, 1 H, Cp-H), 4.27-4.30 (m, 1 H, Cp-H), 4.73-4.76 (m, 1 H, Cp-H), 5.04-5.07 (m, 1 H, Cp-H), 5.08-5.11 (m, 1 H, Cp-H), 5.35-5.38 (m, 1 H, Cp-H), 5.75-5.78 (m, 1 H, Cp-H), 6.37-6.40 (m, 1 H, Cp-H), 6.68-6.73 (m, 1 H, Ph-H), 7.06-7.12 (m, 2 H, Ph-H) ppm. Two Ph-H protons are missing.

**$^{13}$C{$^1$H} NMR** ($C_6D_6$, 125 MHz, 305 K): δ = 22.9 ($CH_3$), 23.8 ($CH_3$), 28.6 (2 x Ad-CH), 29.1 (Ad-CH), 30.0 (Ad-CH), 32.8 (Ad-CH), 32.9 (Ad-$CH_2$), 33.1 (Ad-CH), 33.2 (Ad-$CH_2$), 33.8 (Ad-CH), 36.8 (Ad-CH), 37.5 (Ad-$CH_2$), 38.4 (Ad-$CH_2$), 38.5 (Ad-$CH_2$), 39.3 (Ad-$CH_2$), 39.3 (Ad-$CH_2$), 39.9 (Ad-$CH_2$), 44.2 (Ad-CH), 44.5 (Ad-$CH_2$), 45.5 (Ad-$CH_2$), 98.9 (Cp-CH), 99.9 (Cp-CH), 101.1 (Cp-CH), 101.1 (Cp-CH), 101.6 (Cp-CH), 105.1 (Cp-CH), 106.5 (Cp-CH), 118.4 (Cp-CH), 119.9 ($C_{exo}$-$C_q$), 120.2 ($C_{ipso}$-$C_q$), 128.0 (Ph-CH), 128.4 (Ph-CH), 129.6 ($C_{ipso}$-$C_q$), 144.6 (ketimine-C), 152.9 (Ph-$C_q$) ppm.

**$^{15}$N NMR** ($C_6D_6$, 51 MHz, 305 K): δ = 160.5 (N-Ph), 282.5 (N=$CMe_2$) ppm.

**IR** (ATR): ṽ = 2898, 2845, 1588, 1477, 1445, 1293, 1249, 1211, 1166, 1114, 1098, 1060, 1043, 950,844, 801, 776, 749, 710, 693, 575 $cm^{-1}$.

**Melting point**: 155 – 158 °C.

**EA**: calcd. for $C_{39}H_{48}N_2Ti$: C 79.03, H 8.16, N 4.73. Found: C 78.95, H 8.51, N 4.36.

## Synthesis of Ti1e:

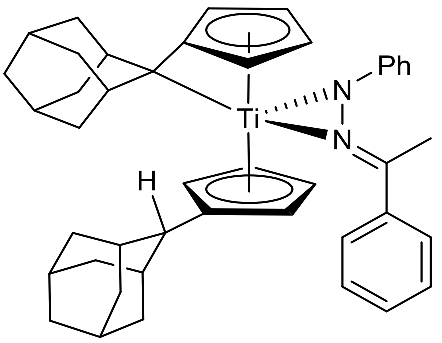


Bis(adamantylidenefulvene)titanium complex **Ti1** (200 mg, 0.450 mmol) and acetophenone phenylhydrazone **e** (94.6 mg, 0.450 mmol) were dissolved in 10 ml of dry toluene. The reaction mixture was stirred for 16 h at room temperature to give a dark brown solution. The solvent was removed under reduced pressure and the residue was washed with 10 mL of *n*-hexane. All volatile components were removed under reduced pressure and the residue was dried under vacuum to yield the product as a brown solid.

**Yield**: 0.208 g, 0.318 mmol, 71%.

**$^1$H NMR** ($C_6D_6$, 500 MHz, 305 K): δ = 1.46 (s, 2 H, Ad-H), 1.60-2.30 (m, 25 H, Ad-H), 1.91 (s, 3 H, $CH_3$), 2.54 (s, 1 H, Ad-H), 2.76 (s, 1 H, Ad-H), 4.06-4.09 (m, 1 H, Cp-H), 4.35-4.37 (m, 1 H, Cp-H), 4.79-4.82 (m, 1 H, Cp-H), 5.27-5.30 (m, 1 H, Cp-H), 5.31-5.34 (m, 1 H, Cp-H), 5.56-5.58 (m, 1 H, Cp-H), 5.70-5.73 (m, 1 H, Cp-H), 6.49-6.51 (m, 1 H, Cp-H), 6.71-6.75 (m, 1 H, Ph-H), 7.17-7.26 (m, 6 H, Ph-H), 7.68-7.72 (m, 2H, Ph-H), 7.75-7.78 (m, 1H, Ph-H) ppm.

**$^{13}$C{$^1$H} NMR** ($C_6D_6$, 125 MHz, 305 K): δ = 20.6 ($CH_3$), 28.5 (Ad-CH), 28.6 (Ad-CH), 29.0 (Ad-CH), 32.7 (Ad-CH), 33.0 (Ad-CH), 33.2 (Ad-$CH_2$), 33.9 (Ad-CH), 36.6 (Ad-CH), 37.3 (Ad-CH), 37.6 (Ad-$CH_2$), 38.3 (Ad-$CH_2$), 38.4 (Ad-$CH_2$), 39.2 (Ad-$CH_2$), 39.5 (Ad-$CH_2$), 39.8 (Ad-$CH_2$), 40.2 (Ad-$CH_2$), 44.6 (Ad-CH), 44.9 (Ad-$CH_2$), 45.6 (Ad-$CH_2$), 97.9 (Cp-CH), 100.4 (Cp-CH), 100.8 (Cp-CH), 100.8 (Cp-CH), 101.7 (Cp-CH), 108.1 (Cp-CH), 109.3 (Cp-CH), 113.8 (2 x Ph-CH), 119.9 (Ph-CH), 120.0 (Cp-CH), 120.4 (Ph-CH), 120.6 (Ph-CH), 122.1 ($C_{exo}$-$C_q$), 125.6 (2 x Ph-CH), 125.9 (2 x Ph-CH), 129.6 (Ph-CH), 130.3 ($C_{ipso}$-$C_q$), 137.5 ($C_{ipso}$-$C_q$), 144.2 (ketimine-$C_q$), 145.9 (Ph-$C_q$), 153.2 (Ph-$C_q$) ppm.

**$^{15}$N NMR** ($C_6D_6$, 51 MHz, 305 K): δ = 176.0 (N-Ph), 288.8 (N=$CMe_2$) ppm.

**IR** (ATR): ṽ = 2899, 2847, 1447, 1249, 1098, 1026, 787, 773, 756, 689, 644, 626, 599 $cm^{-1}$.

**Melting point**: 103 – 108 °C.

EA: calcd. for $C_{44}H_{50}N_2Ti$: C 80.71, H 7.70, N 4.28. Found: 81.03, H 8.02, N 4.39.

## Synthesis of Ti1f:

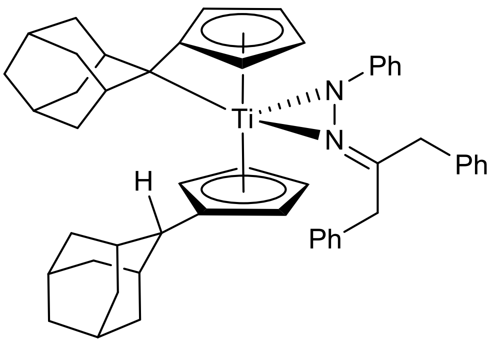


Bis(adamantylidenefulvene)titanium complex **Ti1** (200 mg, 0.450 mmol) and 1,3-diphenylacetone phenylhydrazone **f** (135 mg, 0.449 mmol) were dissolved in 10 ml of dry toluene. The reaction mixture was stirred for 16 h at room temperature to give a dark brown solution. The solvent was removed under reduced pressure and the residue was washed with 10 mL of *n*-hexane. All volatile components were removed under reduced pressure and the residue was dried under vacuum to yield the product as a red brown solid. Red orange crystals suitable for single crystal X-ray diffraction analysis precipitated from a slowly evaporating solution of **Ti2f** in $C_6D_6$ after several days.

**Yield**: 0.277 g, 0.372 mmol, 83%.

**$^1$H NMR** ($C_6D_6$, 500 MHz, 305 K): δ = 1.46-2.30 (m, 27 H, Ad-H), 2.55 (s, 1 H, Ad-H), 3.17 (d, 1 H, $CH_2$-H), 3.34 (s, 1 H, $C_{exo}$H), 3.56 (d, 1 H, $CH_2$-H), 3.61 (d, 1 H, $CH_2$-H), 3.68 (d, 1 H, $CH_2$-H), 4.00-4.04 (m, 1 H, Cp-H), 4.22-4.26 (m, 1 H, Cp-H), 4.92-4.96 (m, 1 H, Cp-H), 4.97-5.00 (m, 1 H, Cp-H), 5.42-5.45 (m, 1 H, Cp-H), 5.45-5.48 (m, 1 H, Cp-H), 5.76-5.79 (m, 1 H, Cp-H), 6.03-6.07 (m, 1 H, Cp-H), 6.68-6.74 (m, 3 H, Ph-H), 6.98-7.11 (m, 7 H, Ph-H), 7.12-7.15 (m, 2 H, Ph-H), 7.17-7.19 (m, 3 H, Ph-H) ppm.

**$^{13}$C{$^1$H} NMR** ($C_6D_6$, 125 MHz, 305 K): δ = 28.6 (2 x Ad-CH), 29.1 (Ad-CH), 30.0 (Ad-CH), 32.6 (Ad-CH), 32.9 (Ad-$CH_2$), 33.0 (Ad-$CH_2$), 33.7 (Ad-CH), 34.3 (Ad-CH), 36.7 (Ad-CH), 37.7 (Ad-$CH_2$), 38.4 (Ad-$CH_2$), 38.5 (Ad-$CH_2$), 39.2 (Ad-$CH_2$), 39.4 (Ad-$CH_2$), 39.5 (Ad-$CH_2$), 40.6 ($CH_2$), 42.4 ($CH_2$), 44.7 (Ad-CH), 45.0 (Ad-$CH_2$), 45.5 (Ad-$CH_2$), 93.0 (Cp-CH), 99.5 (Cp-CH), 100.8 (Cp-CH), 102.5 (Cp-CH), 104.8 (Cp-CH), 106.0 (Cp-CH), 112.7 (Cp-CH), 117.9 (Cp-CH), 119.5 (Ph-CH), 120.0 ($C_{ispo}$-$C_q$), 121.7 ($C_{exo}$-$C_q$), 126.7 (Ph-CH), 127.0 (Ph-CH), 128.4 (2 x Ph-CH), 128.6 (2 x Ph-CH), 129.0 (2 x Ph-CH), 129.2 (2 x Ph-CH), 129.8 (2 x Ph-CH), 137.4 (Ph-$C_q$), 138.3 (Ph-$C_q$), 148.5 (Cp-$C_q$), 153.2 (Ph-$C_q$) ppm. Two Ph-CH, one $C_{ispo}$-$C_q$ and ketimine carbon atom signals are missing.

**$^{15}$N NMR** ($C_6D_6$, 51 MHz, 305 K): δ = 289.0 (N=C) ppm.

**IR** (ATR): ṽ = 2899, 2847, 1493, 1482, 1450, 1239, 1096, 1073, 1054, 1030, 848, 792, 745, 718, 696, 666, 573 $cm^{-1}$.

**Melting point**: 155 – 159 °C.

**EA**: calcd. for $C_{51}H_{56}N_2Ti$: C 82.24, H 7.58, N 3.76. Found: C 82.12, H 7.34, N 3.58.

## Synthesis of Ti1h:

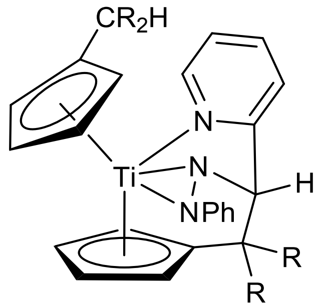


$CR_2$ = Ad

Bis(adamantylidenefulvene)titanium complex **Ti1** (200 mg, 0.450 mmol) and 2-pyridinecarboxaldehyde phenylhydrazone **h** (97.3 mg, 0.449 mmol) were dissolved in 10 ml of dry toluene. The reaction mixture was stirred for 16 h at room temperature to give a dark red solution. The solvent was removed under reduced pressure and the residue was washed with 10 mL of *n*-hexane. All volatile components were removed under reduced pressure and the residue was dried under vacuum to yield the product as a dark red solid. Red crystals suitable for single crystal X-ray diffraction analysis precipitated from a slowly evaporating solution of **Ti1h** in $C_6D_6$ after several days.

**Yield**: 0.241 g, 0.377 mmol, 84%.

**$^1$H NMR** ($C_6D_6$, 500 MHz, 305 K): δ = 0.91-2.28 (m, 27 H, Ad-H), 2.64-2.68 (m, 1 H, Ad-H), 3.49 (s, 1 H, Ad-H), 4.75-4.77 (m, 1 H, Cp-H), 5.04-5.06 (m, 1 H, Cp-H), 5.30-5.37 (m, 4 H, Cp-H), 5.53-5.56 (m, 1 H, Cp-H), 6.10 (s, 1 H, N-$CR_2$H), 6.51-6.54 (m, 1 H, Py-CH), 6.76-6.80 (m, 1 H, Ph-H), 6.88-6.97 (m, 2 H, Ph-H), 7.19-7.21 (m, 1 H, Py-H), 7.29-7.37 (m, 4 H, Cp-H, Ph-H, Py-H), 7.95-7.98 (m, 1 H, Py-H) ppm.

**$^{13}$C{$^1$H} NMR** ($C_6D_6$, 125 MHz, 305 K): δ = 28.0 (Ad-CH), 28.2 (Ad-CH), 28.4 (2 x Ad-CH), 31.4 (Ad-CH), 32.1 (Ad-$CH_2$), 32.4 (Ad-$CH_2$), 32.5 (Ad-$CH_2$), 33.5 (Ad-$CH_2$), 33.7 (Ad-$CH_2$), 34.6 (Ad-$CH_2$), 35.0 (Ad-$CH_2$), 35.7 (Ad-CH), 38.4 (Ad-$CH_2$), 38.9 (Ad-$CH_2$), 39.0 (Ad-CH), 39.1 (Ad-$CH_2$), 43.4 (Ad-CH), 51.3 (Ad-CH), 86.2 (N-$CR_2$H), 96.2 (Cp-CH), 99.3 (Cp-CH), 102.0 (Cp-CH), 106.9 (Cp-CH), 109.1 (Cp-CH), 113.4 (Ph-CH), 115.0 (Cp-CH), 116.7 (Cp-CH), 121.4 (Cp-CH), 121.8 (Py-CH), 122.4 ($C_{ipso}$-$C_q$), 122.4 ($C_{exo}$-$C_q$), 123.4 (Py-CH), 123.7 (Cp-CH), 128.6 (Ph-CH), 129.3 (Ph-CH), 129.5 (Ph-CH), 130.4 (Ph-$C_q$), 135.5 (Ph-CH), 135. 7 (Py-CH), 150.4 (Py-CH), 163.5 (Cp-$C_q$), 168.5 (Py-$C_q$) ppm.

**$^{15}$N NMR** ($C_6D_6$, 51 MHz, 305 K): δ = 222.7 (N-Ph), 289.0 (N-$CHR_2$) ppm.

**IR** (ATR): ṽ = 2900, 2849, 1635, 1583, 1542, 1468, 1435, 1394, 1300, 1282, 1220, 1198, 988, 931, 906, 870, 836, 794, 773, 725, 697, 662, 630 $cm^{-1}$.

**Melting point**: 145 – 150 °C.

**EA**: calcd. for $C_{42}H_{47}N_3Ti$: C 78.61, H 7.38, N 6.55. Found: C 77.95, H 7.73, N 6.28.

## Synthesis of Ti1i:

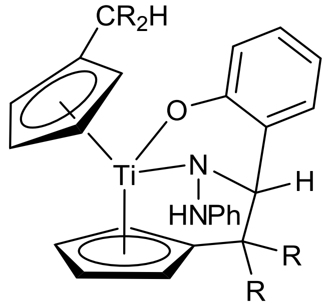


CR$_2$ = Ad

Bis(adamantylidenefulvene)titanium complex **Ti1** (200 mg, 0.450 mmol) and salicylaldehyde phenylhydrazone **i** (95.5 mg, 0.450 mmol) were dissolved in 10 ml of dry toluene at 0°C. The reaction mixture was stirred for 15 min at room temperature to give a dark red solution. The solvent was immediately removed under reduced pressure and the residue was washed with 10 mL of cold *n*-hexane. All volatile components were removed under reduced pressure and the residue was dried under vacuum to yield the product as a red solid. Red orange crystals suitable for single crystal X-ray diffraction analysis precipitated from a solution of **Ti1i** in toluene at -30 °C after several days.

**Yield**: 0.244 g, 0.372 mmol, 83%.

**$^1$H NMR** ($C_6D_6$, 500 MHz, 305 K): δ = 1.43-2.09 (m, 24 H, Ad-H), 2.29-2.38 (m, 3 H, Ad-H), 2.68-2.72 (m, 1 H, Ad-H), 3.19-3.22 (m, 1 H, Ad-H), 5.01 (s, 1 H, N-CR$_2$H), 5.17-5.20 (m, 1 H, Cp-H), 5.27-5.30 (m, 1 H, Cp-H), 5.54-5.57 (m, 1 H, Cp-H), 5.74-5.77 (m, 1 H, Cp-H), 5.82 (s, 1 H, NH), 5.83-5.85 (m, 1 H, Cp-H), 5.97-6.00 (m, 1 H, Cp-H), 6.05-6.07 (m, 1 H, Cp-H), 6.07-6.09 (m, 1 H, Cp-H), 6.44-6.50 (m, 2 H, Ar-H), 6.77-6.81 (m, 2 H, Ar-H), 6.97-7.00 (m, 1 H, Ar-H), 7.18-7.22 (m, 2 H, Ar-H), 7.23-7.27 (m, 2 H, Ar-H) ppm.

**$^{13}$C{$^1$H} NMR** ($C_6D_6$, 125 MHz, 305 K): δ = 27.9 (Ad-CH), 28.2 (Ad-CH), 28.5 (Ad-CH), 32.0 (Ad-CH), 32.4 (Ad-CH), 32.8 (Ad-CH$_2$), 33.0 (Ad-CH), 33.1 (Ad-CH$_2$), 33.7 (Ad-CH), 33.8 (Ad-CH$_2$), 34.1 (Ad-CH$_2$), 35.3 (Ad-CH$_2$), 35.5 (Ad-CH$_2$), 38.3 (Ad-CH$_2$), 38.8 (Ad-CH$_2$), 39.2 (Ad-CH$_2$), 39.4 (Ad-CH$_2$), 44.4 (Ad-CH), 47.2 (Ad-CH), 95.3 (N-CR$_2$H), 103.4 (Cp-CH), 106.0 (Cp-CH), 107.9 (Cp-CH), 108.5 (Cp-CH), 112.6 (2 x Ar-CH), 113.2 (Ar-CH), 117.5 (Cp-CH), 117.6 (Ar-CH), 118.3 (Cp-CH), 119.5 (Cp-CH), 120.3 (Ar-CH), 124.9 (Cp-CH), 125.7 ($C_{exo}$-$C_q$), 126.1 ($C_{ipso}$-$C_q$), 128.6 (Ar-CH), 129.3 (Ar-CH), 129.4 (Ar-CH), 133.4 (Ar-CH), 140.3 (Ar-$C_q$), 142.6 (Ar-$C_q$) ,149.7 (Ar-$C_q$), 165.3 (Cp-$C_q$) ppm.

**$^{15}$N NMR** ($C_6D_6$, 51 MHz, 305 K): δ = 142.2 (d, N-H), 292.1 (N-CR$_2$H) ppm.

**IR** (ATR): ṽ = 2900, 2848, 2671, 2359, 1595, 1557, 1512, 1491, 1468, 1448, 1439, 1353, 1329, 1304, 1271, 1258, 1217, 1168, 1147, 1098, 1061, 1035, 1014, 990, 972, 935, 886, 875, 851, 830, 812, 780, 750, 740, 727, 692, 656, 639, 626, 559 cm$^{-1}$.

**Melting point**: 164 – 166 °C. (dec.)

**EA**: calcd. for $C_{43}H_{48}N_2OTi$: C 78.64, H 7.37, N 4.27. Found: C 78.19, H 7.49, N 4.18.

## Synthesis of Ti1i*:

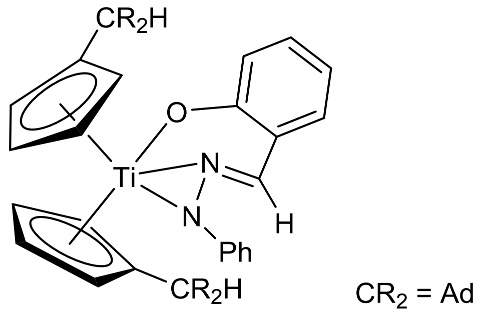


Bis(adamantylidenefulvene)titanium complex **Ti1** (200 mg, 0.450 mmol) and salicylaldehyde phenylhydrazone **i** (95.5 mg, 0.450 mmol) were dissolved in 10 ml of dry toluene. The reaction mixture was stirred for two weeks at 60°C to give a dark red solution. The solvent was removed under reduced pressure and the residue was washed with 10 mL of *n*-hexane. All volatile components were removed under reduced pressure and the residue was dried under vacuum to yield the product as a red solid. Red crystals suitable for single crystal X-ray diffraction analysis precipitated from a slowly evaporating solution of **Ti1i*** in $C_6D_6$ after several days.

**Yield**: 0.254 g, 0.387 mmol, 86%.

**$^1$H NMR** ($C_6D_6$, 500 MHz, 305 K): δ = 1.28-1.34 (m, 2 H, Ad-H), 1.44-1.48 (m, 2 H, Ad-H), 1.61-1.89 (m, 24 H, Ad-H), 2.82 (s, 2 H, $C_{exo}$-H), 5.33-5.36 (m, 2 H, Cp-H), 5.65-5.67 (m, 2 H, Cp-H), 5.82-5.85 (m, 2 H, Cp-H), 5.86-5.88 (m, 2 H, Cp-H), 6.68-6.72 (m, 1 H, Ar-H), 6.90-6.93 (m, 1 H, Ar-H), 6.96-6.99 (m, 2 H, Ar-H), 6.99-7.01 (m, 1 H, Ar-H), 7.19-7.22 (m, 1 H, Ar-H), 7.26-7.30 (m, 2 H, Ar-H), 7.78 (s, 1 H, aldimine-H) ppm.

**$^{13}$C{$^1$H} NMR** ($C_6D_6$, 125 MHz, 305 K): δ = 28.3 (2 x Ad-CH), 28.4 (2 x Ad-CH), 32.0 (2 x Ad-CH), 32.5 (2 x Ad-CH), 32.6 (2 x Ad-$CH_2$), 32.9 (2 x Ad-$CH_2$), 38.2 (2 x Ad-$CH_2$), 38.9 (2 x Ad-$CH_2$), 39.2 (2 x Ad-$CH_2$), 43.6 (2 x $C_{exo}$-CH), 105.3 (2 x Cp-CH), 112.4 (2 x Cp-CH), 112.6 (2 x Cp-CH), 114.3 (2 x Cp-CH), 115.6 (Ar-CH), 116.7 (2 x Ph-CH), 120.5 (Ar-CH), 121.9 (Ar-CH), 123.3 (Ar-$C_q$), 125.6 (aldimine-CH), 128.4 (Ar-CH), 129.8 (2 x Ar-CH), 130.2 (Ar-CH), 139.1 (2 x $C_{ipso}$-$C_q$), 150.6 (Ar-$C_q$), 161.9 (Ar-$C_q$) ppm.

**$^{15}$N NMR** ($C_6D_6$, 51 MHz, 305 K): δ = 162.6 (N-Ph), 266.7 (N=CHR) ppm.

**IR** (ATR): ṽ = 2899, 2847, 1583, 1485, 1467, 1449, 1361, 1312, 1259, 1217, 1199, 1169, 1148, 1098, 1061, 1031, 904, 875, 801, 779, 746, 687, 596 $cm^{-1}$.

**Melting point**: 92-96 °C. (dec.)

**EA**: calcd. for $C_{43}H_{48}N_2OTi$: C 78.64, H 7.37, N 4.27. Found: C 80.02, H 7.84, N 4.32.

## Synthesis of Ti2a:

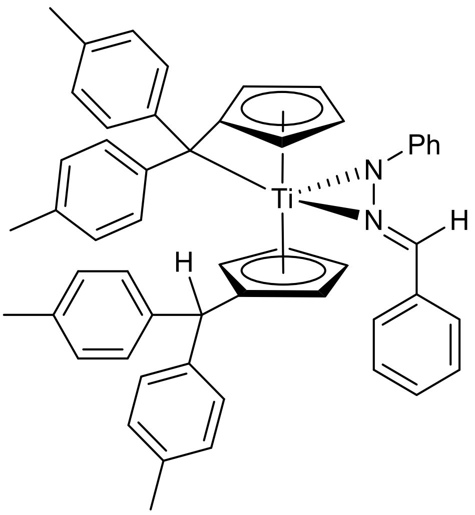


Bis(di-*para*-tolylfulvene)titanium complex **Ti2** (300 mg, 0.531 mmol) and benzaldehyde phenylhydrazone **a** (104 mg, 0.531 mmol) were dissolved in 10 ml of dry toluene. The reaction mixture was stirred for 16 h at room temperature to give a dark brown solution. The solvent was removed under reduced pressure and the residue was washed with 10 mL of *n*-hexane. All volatile components were removed under reduced pressure and the residue was dried under vacuum to yield the product as a red brown solid.

**Yield**: 0.332 g, 0.436 mmol, 82%.

**$^{1}$H NMR** ($C_6D_6$, 500 MHz, 305 K): δ = 2.02-2.17 (m,12 H, *p*-Tol-$CH_3$), 4.56-4.90 (m, 2 H, Cp-H), 4.93 (s, 1 H, $C_{exo}$H), 4.93-5.09 (m, 1 H, Cp-H), 5.14-5.57 (m, 3 H, Cp-H), 5.69-5.90 (m, 1 H, Cp-H), 6.17-6.40 (m, 1 H, Cp-H), 6.55-6.60 (m, 3 H, Ar-H), 6.85-7.00 (m, 7 H, Ar-H), 7.03-7.09 (m, 8 H, Ar-H), 7.18-7.27 (m, 3 H, Ar-H), 7.28-7.56 (m, 5 H, Ar-H), 7.69 (s, 1 H, aldimine-H) ppm.

**$^{13}$C{$^{1}$H} NMR** ($C_6D_6$, 125 MHz, 305 K): δ = 20.9 (2 x *p*-Tol-$CH_3$), 21.0 (2 x *p*-Tol-$CH_3$), 52.6 ($C_{exo}$H), 113.2 (Ar-CH), 120.4 (Ar-CH), 122.6 (Ar-$C_q$), 123.0 (Ar-CH), 123.9 (Ar-CH), 124.9 (Ar-CH), 126.4 (Ar-CH), 126.5 (Ar-CH), 127.4 (Ar-CH), 128.3 (Ar-CH), 128.5 (aldimine-CH), 129.2 (Ar-CH), 129.4 (Ar-CH), 132.7 ($C_{ipso}$-$C_q$), 135.8 (Ar-$C_q$), 137.3 (CH), 147.5 (Ar-$C_q$) ppm. Due to the broadening of signals, several signals cannot be identified and assigned.

**$^{15}$N NMR** ($C_6D_6$, 51 MHz, 305 K): δ = 181.3 (N-Ph), 285.1 (N=CHPh) ppm.

**IR** (ATR): ṽ = 3020, 2919, 2861, 2355, 1594, 1509, 1481, 1445, 1259, 1109, 1054, 1038, 1021, 806, 761, 753, 691 $cm^{-1}$.

**Melting point**: 112 – 117 °C.

**EA**: calcd. for $C_{53}H_{48}N_2Ti$: C 83.67, H 6.36, N 3.68. Found: C 78.65, H 6.36, N 3.95.

## Synthesis of Ti2b:

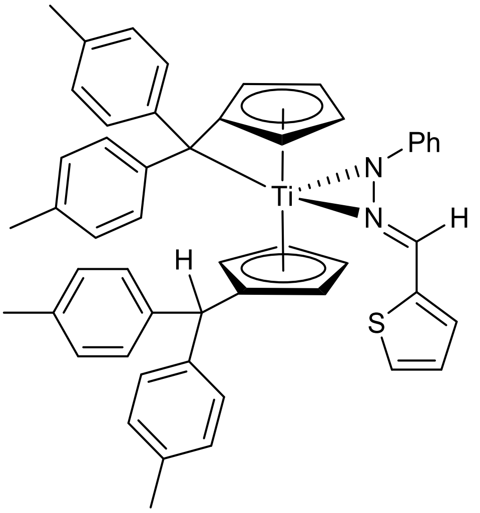


Bis(di-*para*-tolylfulvene)titanium complex **Ti2** (200 mg, 0.354 mmol) and 2-thiophenecarboxaldehyde phenylhydrazone **b** (71.7 mg, 0.354 mmol) were dissolved in 10 ml of dry toluene. The reaction mixture was stirred for 16 h at room temperature to give a dark brown solution. The solvent was removed under reduced pressure and the residue was washed with 10 mL of *n*-hexane. All volatile components were removed under reduced pressure and the residue was dried under vacuum to yield the product as a brown solid.

**Yield**: 0.208 g, 0.271 mmol, 76%.

**$^1$H NMR** ($C_6D_6$, 500 MHz, 305 K): δ = 1.90-2.11 (m,12 H, *p*-Tol-$CH_3$), 4.58-4.82 (m, 2 H, Cp-H), 4.94 (s, 1 H, $C_{exo}$H), 4.94-5.08 (m, 1 H, Cp-H), 5.29-5.47 (m, 2 H, Cp-H), 5.50-5.62 (m, 1 H, Cp-H), 5.92-6.06 (m, 1 H, Cp-H), 6.25-6.36 (m, 1 H, Cp-H), 6.41-6.43 (m, 1 H, Ar-H), 6.55-6.60 (m, 3 H, Ar-H), 6.62-6.65 (m, 2 H, Ar-H), 6.66-6.75 (m, 2 H, Ar-H), 6.84-6.91 (m, 3 H, Ar-H), 6.95-7.07 (m, 7 H, Ar-H), 7.19-7.27 (m, 2 H, Ar-H), 7.32-7.50 (m, 4 H, Ar-H), 7.94 (s, 1 H, aldimine-H) ppm.

**$^{13}$C{$^1$H} NMR** ($C_6D_6$, 125 MHz, 305 K): δ = 21.0 (4 x *p*-Tol-$CH_3$), 52.6 ($C_{exo}$H), 113.2 (Ar-CH), 122.6 (Ar-$C_q$), 122.8 (Ar-CH), 123.3 (Ar-CH), 123.7 (aldimine-CH), 124.0 (Ar-CH), 124.2 (Ar-CH), 126.9 (Ar-CH), 128.3 (Ar-CH), 129.2 (Ar-CH), 129.4 (Ar-CH), 132.4 ($C_{ipso}$-$C_q$), 135.8 (Ar-$C_q$), 140.6 (Ar-$C_q$), 147.5 (Ar-$C_q$) ppm. Due to the broadening of signals, several signals cannot be identified and assigned.

**$^{15}$N NMR** ($C_6D_6$, 51 MHz, 305 K): δ = 180.6 (N-Ph), 282.4 (N=CHAr) ppm.

**IR** (ATR): ṽ = 3018, 2917, 2859, 1589, 1538, 1507, 1481, 1449, 1362, 1295, 1259, 1186, 1169, 1109, 1074, 1039, 1021, 866, 846, 806, 763, 750, 736, 723, 692, 575 $cm^{-1}$.

**Melting point**: 122 – 126 °C.

**EA**: calcd. for $C_{51}H_{46}N_2STi$: C 79.77, H 6.17, N 3.65. Found: C 78.50, H 6.43, N 3.56.

## Synthesis of Ti2d:

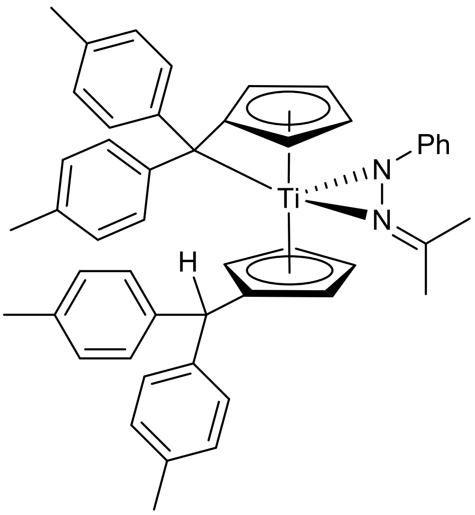


Bis(di-*para*-tolylfulvene)titanium complex **Ti2** (300 mg, 0.531 mmol) and acetone phenylhydrazone **g** (78.8 mg, 0.531 mmol) were dissolved in 10 ml of dry toluene. The reaction mixture was stirred for 16 h at room temperature to give a dark brown solution. The solvent was removed under reduced pressure and the residue was washed with 10 mL of *n*-hexane. All volatile components were removed under reduced pressure and the residue was dried under vacuum to yield the product as a black brown solid.

**Yield**: 0.318 g, 0.447 mmol, 84%.

**$^{1}$H NMR** ($C_6D_6$, 500 MHz, 305 K): δ = 1.41 (s, 3 H, N=C-$CH_3$), 1.56 (s, 3 H, N=C-$CH_3$), 2.08-2.17 (m, 12 H, p-Tol-$CH_3$), 4.55-5.60 (m, 1 H, Cp-H), 4.71-7.76 (m, 1 H, Cp-H), 4.96 (s, 1H, $C_{exo}$H), 5.00-5.13 (m, 3 H, Cp-H), 5.30-5.45 (m, 2 H, Cp-H), 6.30-6.45 (m, 2 H, Cp-H, Ar-H), 6.68-6.72 (m, 1 H, Cp-H), 6.85-6.96 (m, 7 H, Ph-H), 7.03-7.06 (m, 2 H, Ph-H), 7.07-7.12, 2 H, Ph-H), 7.18-7.22 (m, 2 H, Ph-H), 7.34-7.40 (m, 4 H, Ph-H), 7.45-7.49 (m, 2 H, Ph-H) ppm.

**$^{13}$C{$^{1}$H} NMR** ($C_6D_6$, 125 MHz, 305 K): δ = 21.0 (4 x *p*-Tol-$CH_3$), 23.5 ($CH_3$), 24.1 ($CH_3$), 52.0 ($C_{exo}$-CH), 101.8 (Cp-CH), 113.6 (Cp-CH), 116.7 (Cp-CH), 117.7 (Cp-H), 119.9 (Cp-H), 121.9 (ketimine-$C_q$), 128.4 (Ar-CH), 128.5 (Ar-CH), 128.6 (Ar-CH), 128.7 (Ar-CH), 129.3 (Ar-CH), 129.4 (Ar-CH), 129.7 (Ar-CH), 134.4 ($C_{q,ipso}$), 135.7 (Ar-$C_q$), 135.9 (Ar-$C_q$), 140.8 (Ar-$C_q$), 142.9 (Ar-$C_q$), 143.4 (Ar-$C_q$) ppm. Due to the broadening of signals, several signals cannot be identified and assigned.

**$^{15}$N NMR** ($C_6D_6$, 51 MHz, 305 K): δ = 165.8 (N-Ph), 286.0 (N=$CMe_2$) ppm.

**IR** (ATR): ṽ = 3019, 2919, 2860, 2364, 1600, 1589, 1508, 1476, 1453, 1364, 1244, 1186, 1169, 1110, 1037, 1021, 805, 761, 692 $cm^{-1}$.

**Melting point**: 110 - 115 °C.

**EA**: calcd. for $C_{49}H_{48}N_2Ti$: C 82.57, H 6.79, N 3.93. Found: C 82.26, H 6.99, N 3.82.

## Synthesis of Ti2e:

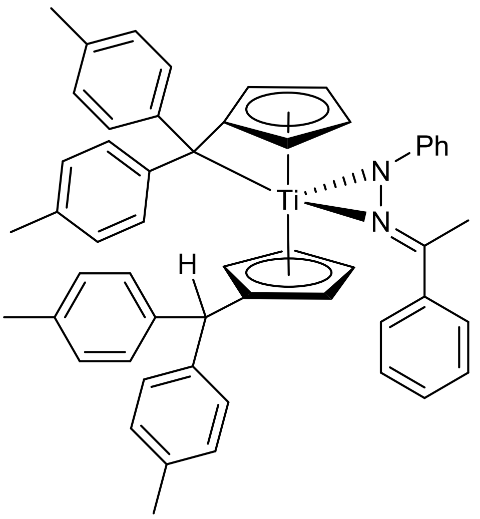


Bis(di-*para*-tolylfulvene)titanium complex **Ti2** (200 mg, 0.354 mmol) and ferrocenecarboxaldehyde phenylhydrazone **e** (74.5 mg, 0.354 mmol) were dissolved in 10 ml of dry toluene. The reaction mixture was stirred for 16 h at room temperature to give a dark brown solution. The solvent was removed under reduced pressure and the residue was washed with 10 mL of *n*-hexane. All volatile components were removed under reduced pressure and the residue was dried under vacuum to yield the product as a brown solid.

**Yield**: 0.236 g, 0.305 mmol, 86%.

**$^{1}$H NMR** ($C_6D_6$, 500 MHz, 305 K): δ = 2.00 (s, 3 H, N=C-$CH_3$), 2.05 (s, 3 H, *p*-Tol-$CH_3$), 2,12-2.14 (m, 9 H, *p*-Tol-$CH_3$), 4.84-4.91 (m, 2 H, Cp-H), 4.94 (s, 1 H, $C_{exo}$H), 4.97-5.02 (m, 1 H, Cp-H), 5.08-5.12 (m, 1 H, Cp-H), 5.13-5.18 (m, 1 H, Cp-H), 5.20-5.27 (m, 1 H, Cp-H), 5.63-5.67 (m, 1 H, Cp-H), 5.99-6.02 (m, 1 H, Cp-H), 6.56-6.59 (m, 2 H, Ar-H), 6.90-6.94 (m, 6 H, Ar-H), 6.65-6.69 (m, 3 H, Ar-H), 6.78-7.27 (m, 12 H, Ar-H), 7.39-7.43 (m, 6 H, Ar-H), 7.45-7.48 (m, 2 H, Ar-H), 7.76-7.79 (m, 1 H, Ar-H) ppm.

**$^{13}$C{$^{1}$H} NMR** ($C_6D_6$, 125 MHz, 305 K): δ = 21.0 (3 x *p*-Tol-$CH_3$), 21.2 (p-Tol-$CH_3$), 21.2 (N=C-$CH_3$), 52.3 ($C_{exo}$-CH), 102.0 (Cp-CH), 102.7 (Cp-CH), 105.1 (Cp-CH), 108.8 (Cp-CH), 111.6 (Cp-CH), 112.6 (Cp-CH), 113.8 (Cp-CH), 117.6 (Cp-CH), 118.6 (Ar-CH), 120.6 (Ar-CH), 124.8 (2 x Ar-CH), 125.9 (2 x Ar-CH), 126.2 (2 x Ar-CH), 128.2 (2 x Ar-CH), 128.6 (2 x Ar-CH), 128.6 (Ar-$C_q$), 128.8 (2 x Ar-CH), 129.2 (2 x Ar-CH), 129.4 (2 x Ar-CH), 129.4 (Ar-$C_q$), 129.6 (2 x Ar-CH), 129.7 (2 x Ar-CH), 132.4 (2 x Ar-CH), 132.7 (2 x Ar-CH), 135.5 ($C_{ipso}$-$C_q$), 136.0 (Ar-$C_q$), 137.2 (Ar-$C_q$), 138.8 (Ar-$C_q$), 139.3 (Ar-$C_q$), 139.8 (Ar-$C_q$), 140.9 (Ar-$C_q$), 143.2 (Ar-$C_q$), 144.3 (Ar-$C_q$), 145.9 (Ar-$C_q$), 152.2 (ketimine-$C_q$), 153.4 (Ar-$C_q$) ppm.

**$^{15}$N NMR** ($C_6D_6$, 51 MHz, 305 K): δ = 176.6 (N-Ph), 291.3 (N=$CMe_2$) ppm.

**IR** (ATR): ṽ = 3019, 2918, 1601, 1588, 1509, 1492, 1445, 1249, 1109, 1039, 1021, 804, 758, 724, 690, 637, 591, 575, 558 $cm^{-1}$.

**Melting point**: 85-90 °C.

**EA**: calcd. for $C_{54}H_{50}N_2Ti$: C 83.70, H 6.50, N 3.62. Found: C 83.31, H 6.74, N 3.52

## Synthesis of Ti2h:

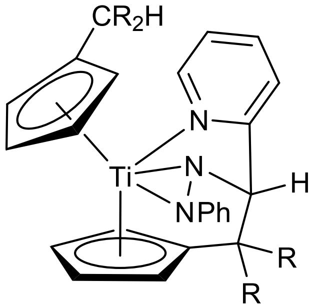


R = *p*-Tol

Bis(di-para-tolylfulvene)titanium complex **Ti2** (200 mg, 0.354 mmol) and 2-pyridinecarboxaldehyde phenylhydrazone **h** (69.9 mg, 0.354 mmol) were dissolved in 10 ml of dry toluene. The reaction mixture was stirred for 16 h at room temperature to give a dark red solution. The solvent was removed under reduced pressure and the residue was washed with 10 mL of *n*-hexane. All volatile components were removed under reduced pressure and the residue was dried under vacuum to yield the product as a red solid. Red crystals suitable for single crystal X-ray diffraction analysis precipitated from a slowly evaporating solution of **Ti2h** in $C_6D_6$ after several days.

**Yield**: 0.228 g, 0.299 mmol, 85%.

**$^1$H NMR** ($C_6D_6$, 500 MHz, 305 K): δ = 1.92 (s, 3 H, *p*-Tol-$CH_3$), 1.98 (s, 3 H, *p*-Tol-$CH_3$), 2.07 (s, 3 H, *p*-Tol-$CH_3$), 2.09 (s, 3 H, *p*-Tol-$CH_3$), 4.89 (s, 1 H, $C_{exo}$H), 5.00-5.03 (m, 1 H, Cp-H), 5.05-5.08 (m, 1 H, Cp-H), 5.19-5.21 (m, 1 H, Cp-H), 5.21-5.24 (m, 1 H, Cp-H), 5.24-5.27 (m, 1 H, Cp-H), 5.27-5.30 (m, 1 H, Cp-H), 5.47-5.50 (m, 1 H, Cp-H), 6.22-6.27 (m, 1 H, Ar-CH), 6.46-6.51 (m, 1 H, Ar-H), 6.65-6.70 (m, 2 H, Ar-H), 6.73-6.78 (m, 4 H, Ar-H), 6.83-6.88 (m, 4 H, Ar-H, N-$CR_2$H), 6.91-6.93 (m, 2 H, Ar-H), 6.95-7.02 (m, 5 H, Ar-H), 7.04-7.13 (m, 3 H, Ar-H), 7.18-7.20 (m, 1 H, Py-H), 7.28-7.32 (m, 2 H, Ar-H), 7.56-7.58 (m, 1 H, Ar-H) ppm.

**$^{13}$C{$^1$H} NMR** ($C_6D_6$, 125 MHz, 305 K): Due to impurities from the byproduct, no sufficiently pure $^{13}$C{$^1$H} NMR spectrum could be measured.

**$^{15}$N NMR** ($C_6D_6$, 51 MHz, 305 K): δ = 221.9 (N-Ph), 285.0 (N-CH) ppm.

**IR** (ATR): ṽ = 3018, 2919, 2856, 1603, 1585, 1509, 1471, 1446, 1293, 1268, 1156, 1100, 1066, 1053, 1039, 1020, 989, 805, 757, 731, 719, 695, 574 $cm^{-1}$.

**Melting point**: 110 – 116 °C.

**EA**: calcd. for $C_{52}H_{47}N_3Ti$: C 81.98, H 6.22, N 5.52. Found: C 78.42, H 6.62, N 4.54.

## Synthesis of Ti2i:

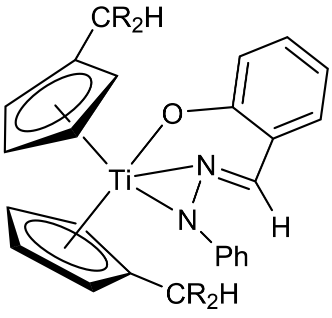


R = *p*-Tol

Bis(di-*p*-tolylfulvene)titanium complex **Ti2** (200 mg, 0.354 mmol) and salicylaldehyde phenylhydrazone **i** (75.2 mg, 0.354 mmol) were dissolved in 10 ml of dry toluene. The reaction mixture was stirred for 16 h at room temperature to give a dark red solution. The solvent was removed under reduced pressure and the residue was washed with 10 mL of *n*-hexane. All volatile components were removed under reduced pressure and the residue was dried under vacuum to yield the product as a red solid.

**Yield**: 0.229 g, 0.295 mmol, 83%.

**$^1$H NMR** ($C_6D_6$, 500 MHz, 305 K): δ = 2.01 (s, 6 H, *p*-Tol-$CH_3$), 2.12 (s, 6 H, *p*-Tol-$CH_3$), 5.11-5.15 (m, 2 H, Cp-H), 5.43 (s, 2 H, $C_{exo}$-H), 5.57-5.60 (m, 2 H, Cp-H), 5.63-5.66 (m, 4 H, Cp-H), 6.71-6.73 (m, 2 H, Ar-H), 6.82-6.85 (m, 5 H, Ar-H), 6.89-6.92 (m, 5 H, Ar-H), 6.98-7.01 (m, 5 H, Ar-H), 7.11-7.15 (m, 3 H, Ar-H), 7.33-7.36 (m, 4 H, Ar-H), 7.45-7.48 (m, 1 H, Ar-H), 7.61 (s, 1 H, aldimine-H) ppm.

**$^{13}$C{$^1$H} NMR** ($C_6D_6$, 125 MHz, 305 K): δ = 20.9 (2 x *p*-Tol-$CH_3$), 21.0 (2 x *p*-Tol-$CH_3$), 51.3 (2 x $C_{exo}$-CH), 105.4 (2 x Cp-CH), 111.8 (2 x Cp-CH), 112.8 (2 x Cp-CH), 116.1 (2 x Cp-CH), 116.3 (Ar-CH), 117.0 (2 x Ar-CH), 120.8 (2 x Ar-CH), 122.3 (2 x Ar-CH), 124.8 (2 x Ar-CH), 125.7 (2 x Ar-CH), 126.3 (aldimine-CH), 128.3 (Ar-$C_q$), 128.4 (2 x Ar-CH), 128.6 (2 x Ar-CH), 129.2 (2 x Ar-CH), 129.3 (Ar-CH), 129.4 (2 x Ar-CH), 129.5 (2 x Ar-CH), 129.6 (Ar-CH), 129.9 (2 x Ar-CH), 130.5 (Ar-CH), 132.7 (Ar-CH), 135.5 (2 x Ar-$C_q$), 135.8 (2 x Ar-$C_q$), 142.1 (2 x $C_{ipso}$-$C_q$), 142.4 (2 x Ar-$C_q$), 142.6 (2 x Ar-$C_q$), 150.5 (Ar-$C_q$), 161.7 (Ar-$C_q$) ppm.

**$^{15}$N NMR** ($C_6D_6$, 51 MHz, 305 K): δ = 160.9 (N-Ph), 265.9 (N=CHPh) ppm.

**IR** (ATR): ṽ = 3019, 2918, 1588, 1509, 1485, 1447, 1362, 1250, 1186, 1170, 1149, 1110, 1090, 1056, 1034, 1021, 904, 868, 806, 761, 748, 690, 641, 615, 596, 574 $cm^{-1}$.

**Melting point**: 116-120 °C. (dec.)

**EA**: calcd. for $C_{53}H_{48}N_2OTi$: C 81.94, H 6.23, N 3.61. Found: C 81.37, H 6.38, N 3.54.

## NMR Spectra of complexes

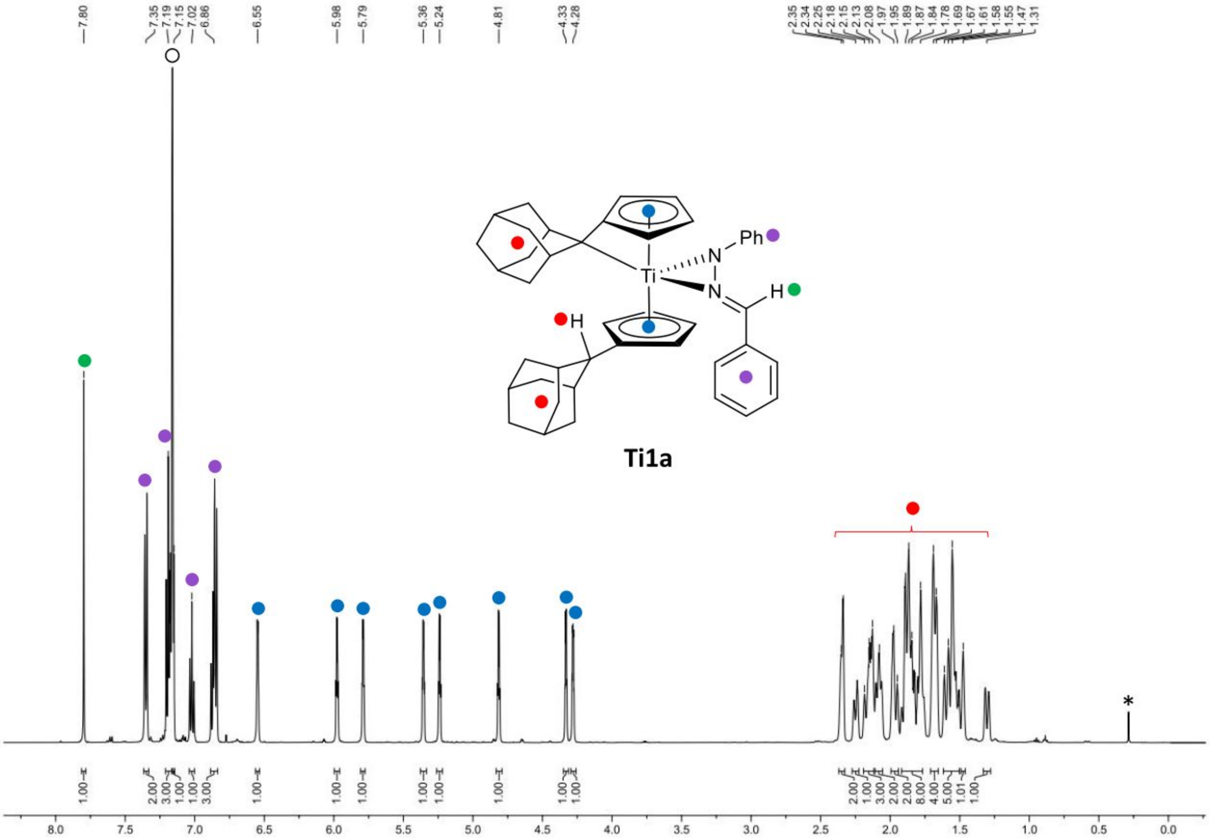


**Figure S1**: $^{1}H$ NMR spectrum (500 MHz, $C_6D_6$, 305 K) of **Ti1a**. Product signals given in colours (° = $C_6H_5D$, * = grease).

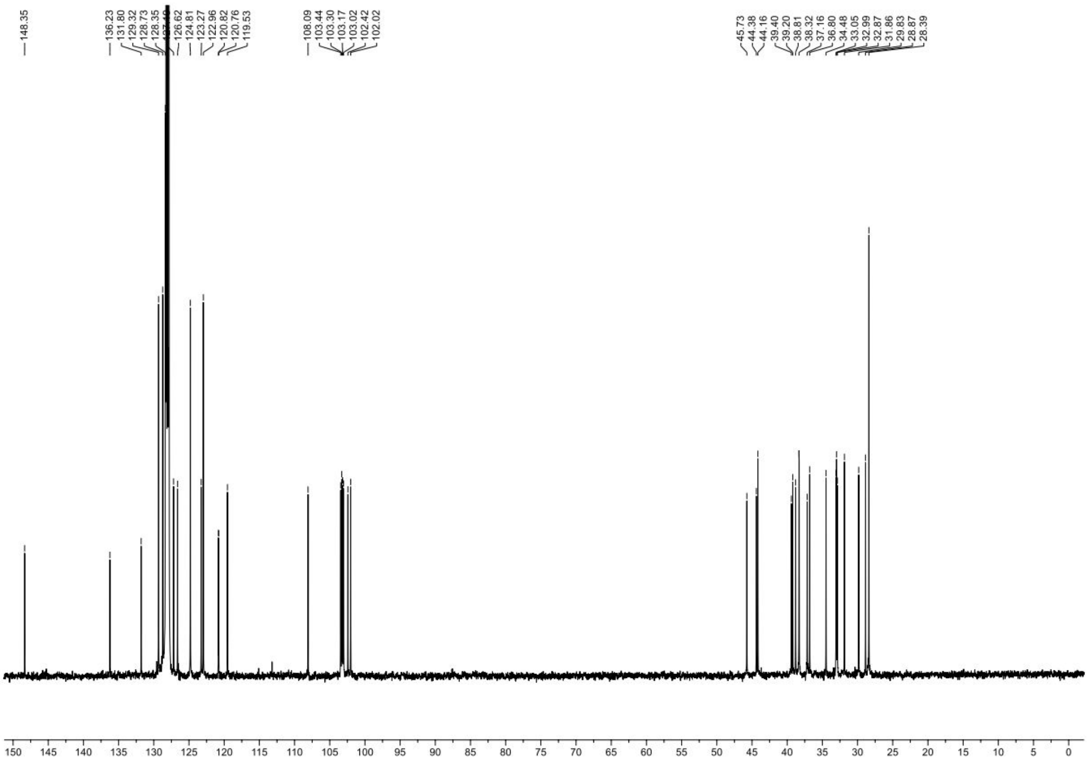

**Figure S2**: $^{13}C$ NMR spectrum (125 MHz, $C_6D_6$, 305 K) of **Ti1a**.

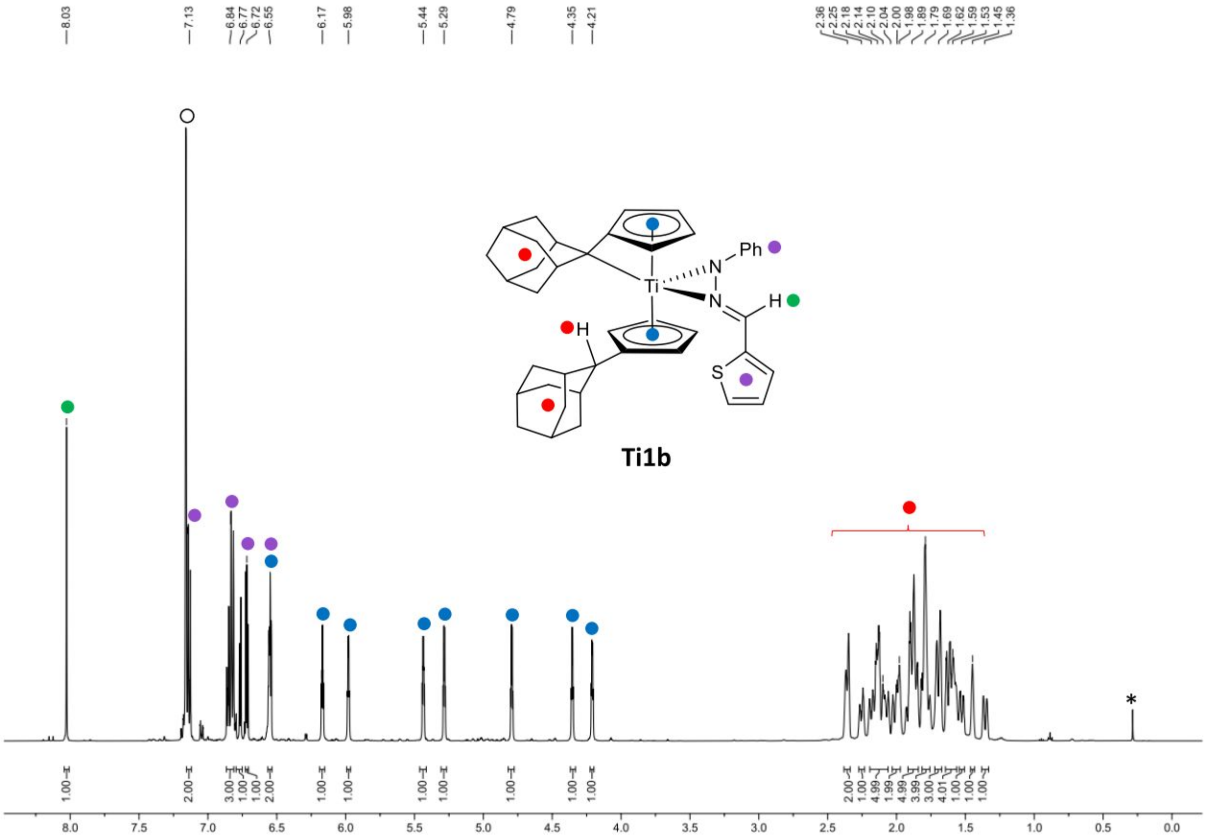


**Figure S3**: $^{1}$H NMR spectrum (500 MHz, $C_6D_6$, 305 K) of **Ti1b**. Product signals given in colours (° = $C_6H_5D$, * = grease).

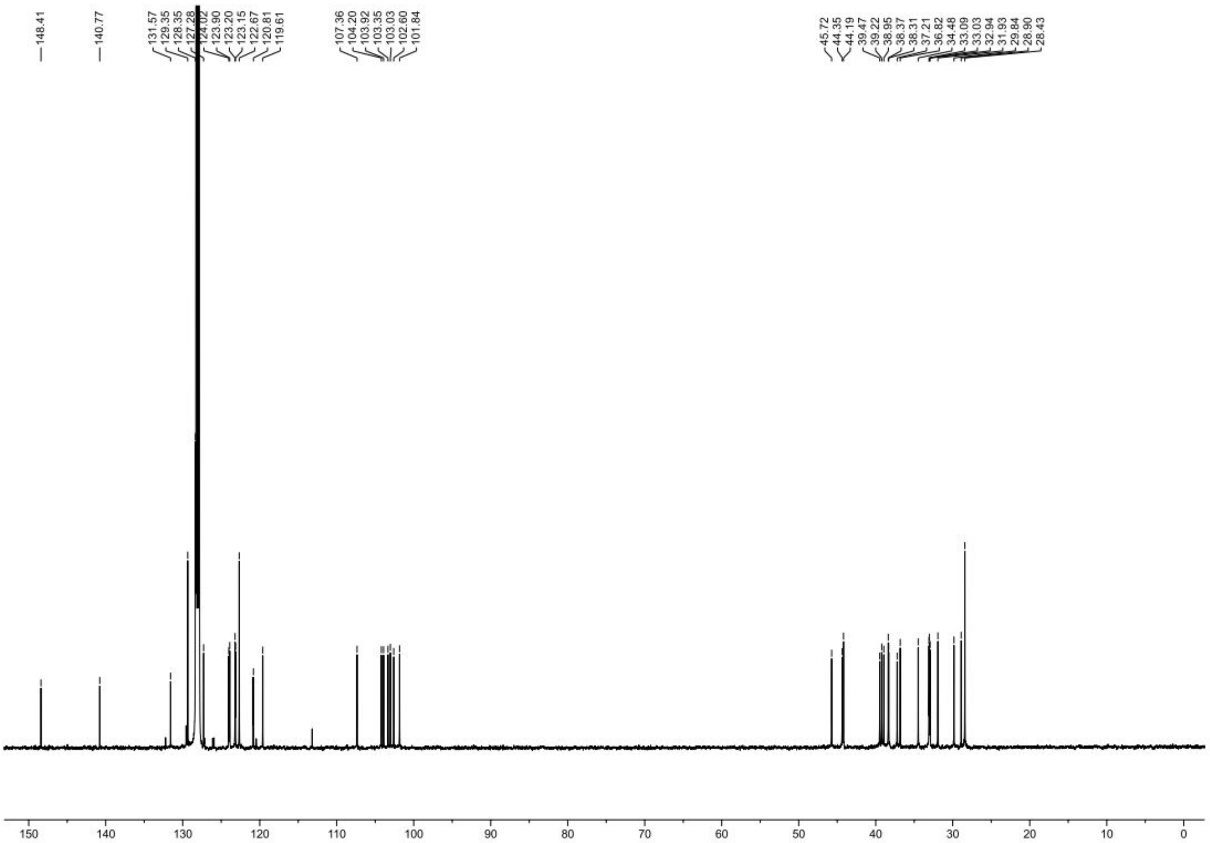

**Figure S4**: $^{13}$C NMR spectrum (125 MHz, $C_6D_6$, 305 K) of **Ti1b**.

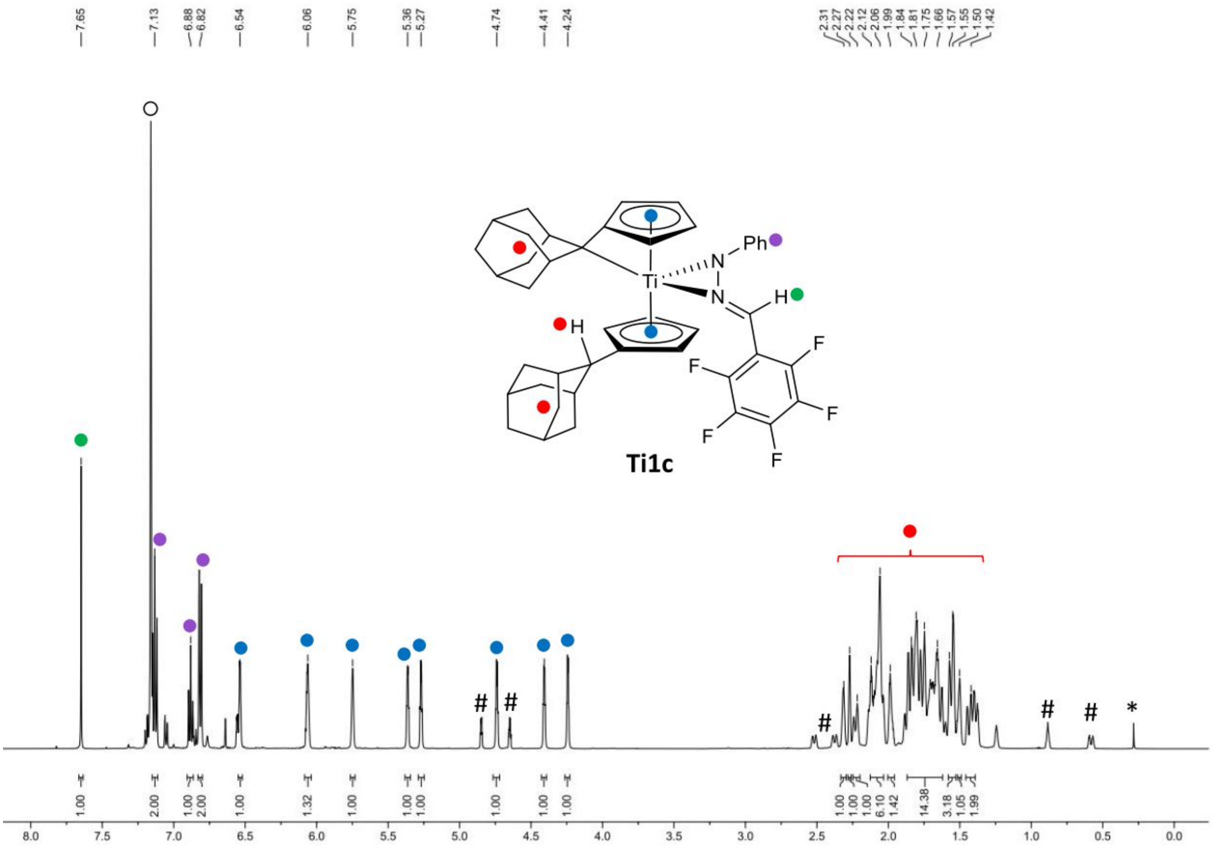

**Figure S5**: $^{1}H$ NMR spectrum (500 MHz, $C_6D_6$, 305 K) of **Ti1c**. Product signals given in colours (o = $C_6H_5D$, * = grease, # = *n*-hexane and traces of **Ti1**).

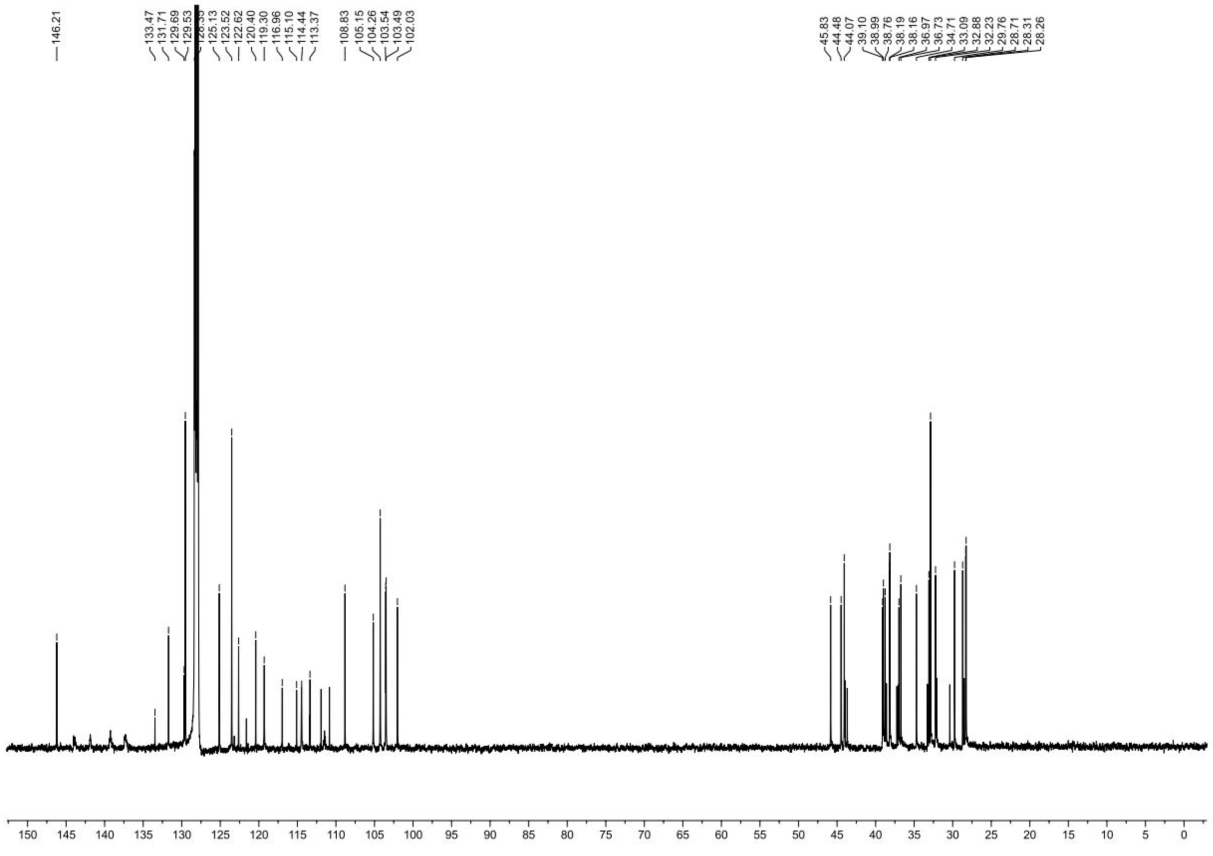

**Figure S6**: $^{13}C$ NMR spectrum (125 MHz, $C_6D_6$, 305 K) of **Ti1c**.

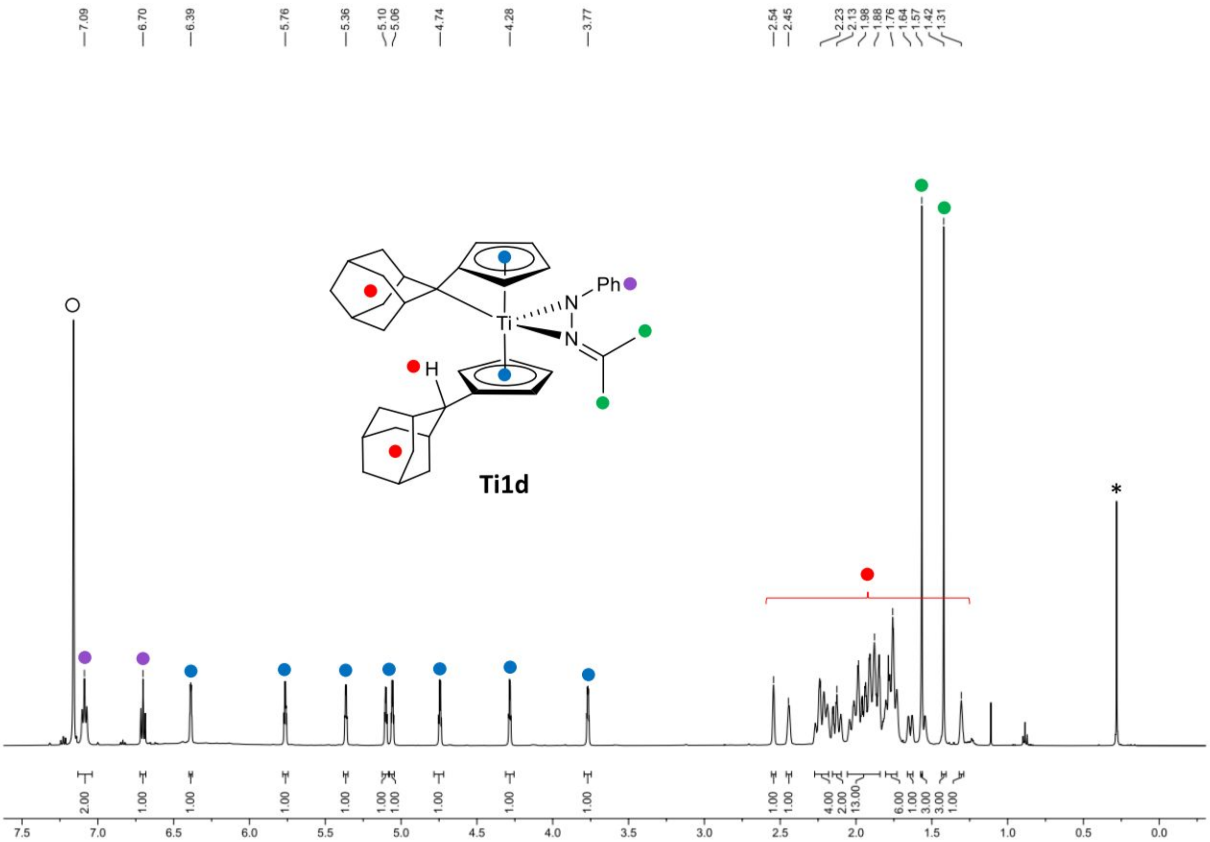


**Figure S7**: $^{1}H$ NMR spectrum (500 MHz, $C_6D_6$, 305 K) of **Ti1d**. Product signals given in colours (° = $C_6H_5D$, * = grease).

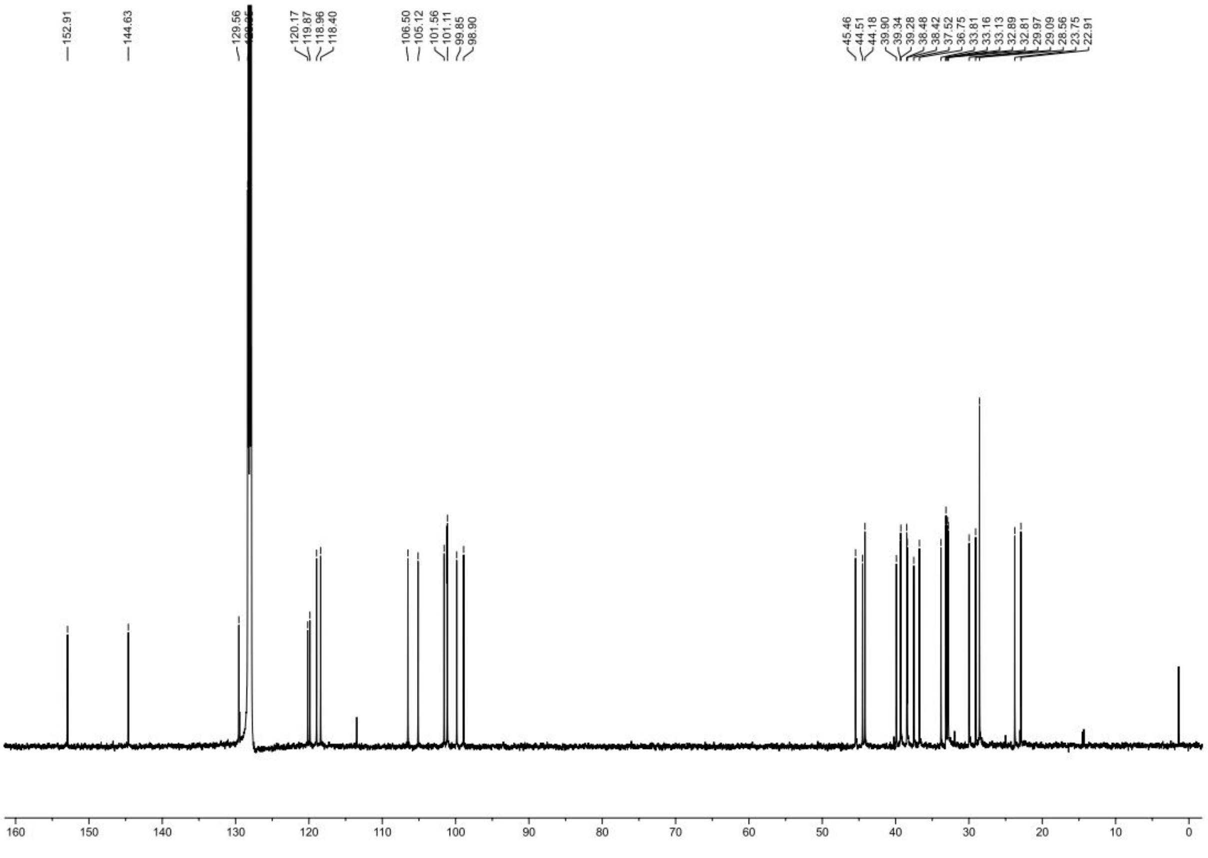


**Figure S8**: $^{13}C$ NMR spectrum (125 MHz, $C_6D_6$, 305 K) of **Ti1d**.

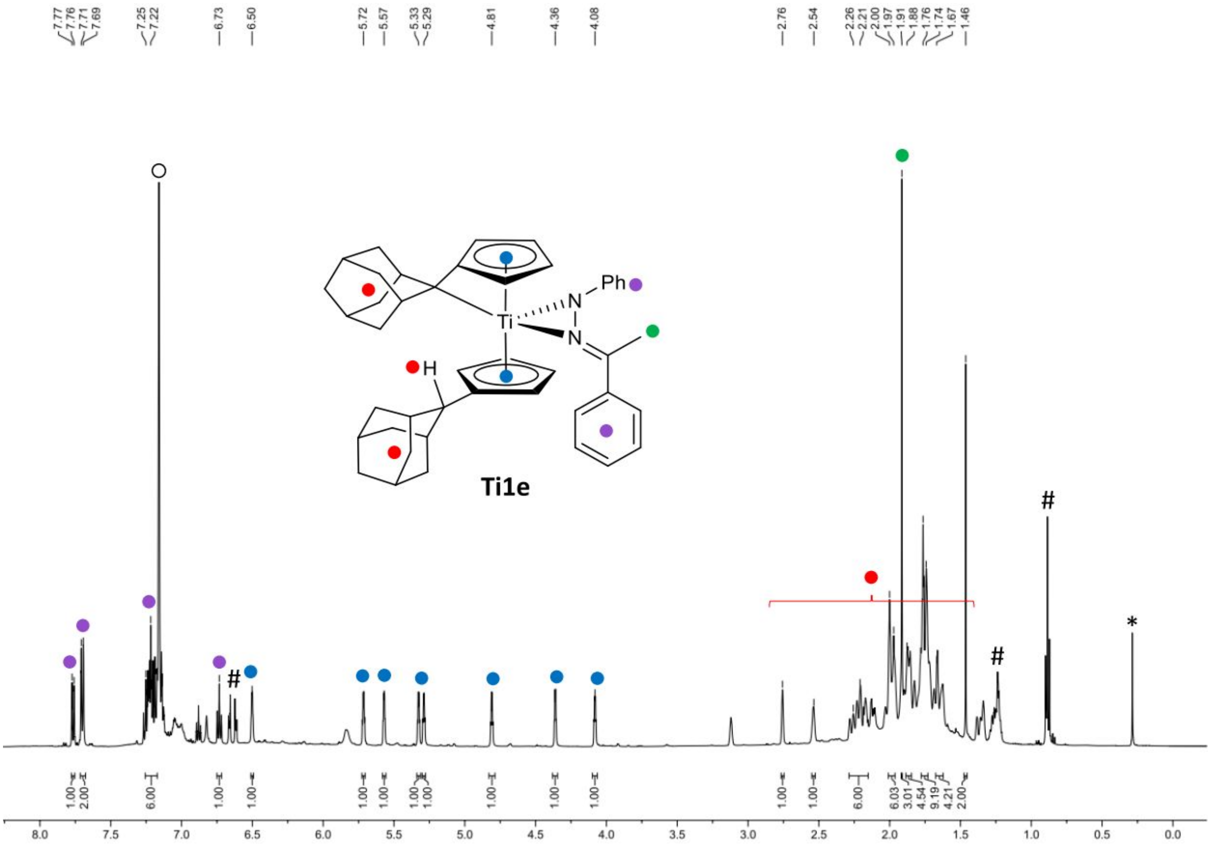


**Figure S9**: $^{1}H$ NMR spectrum (500 MHz, $C_6D_6$, 305 K) of **Ti1e**. Product signals given in colours ($^{o}$ = $C_6H_5D$, * = grease, # = *n*-hexane and free pentafulvene).

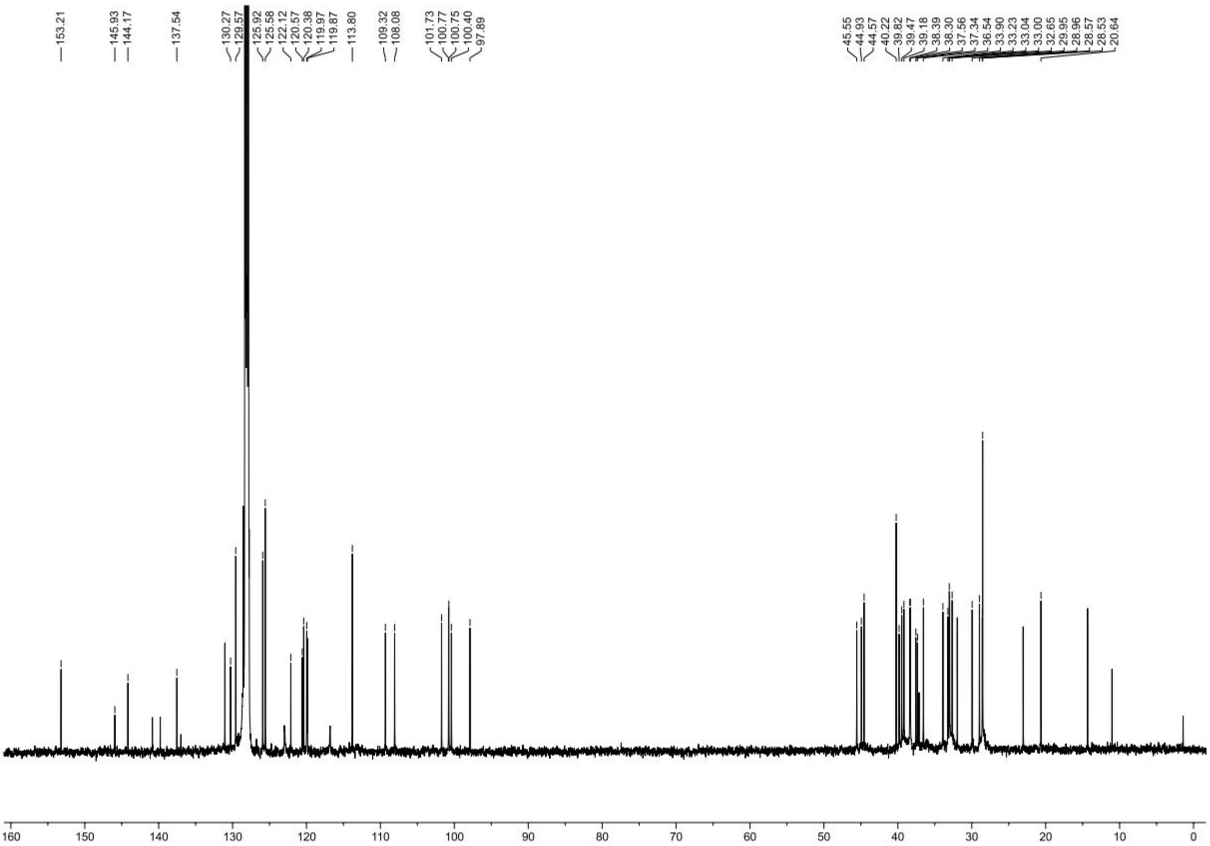


**Figure S10**: $^{13}C$ NMR spectrum (125 MHz, $C_6D_6$, 305 K) of **Ti1e**.

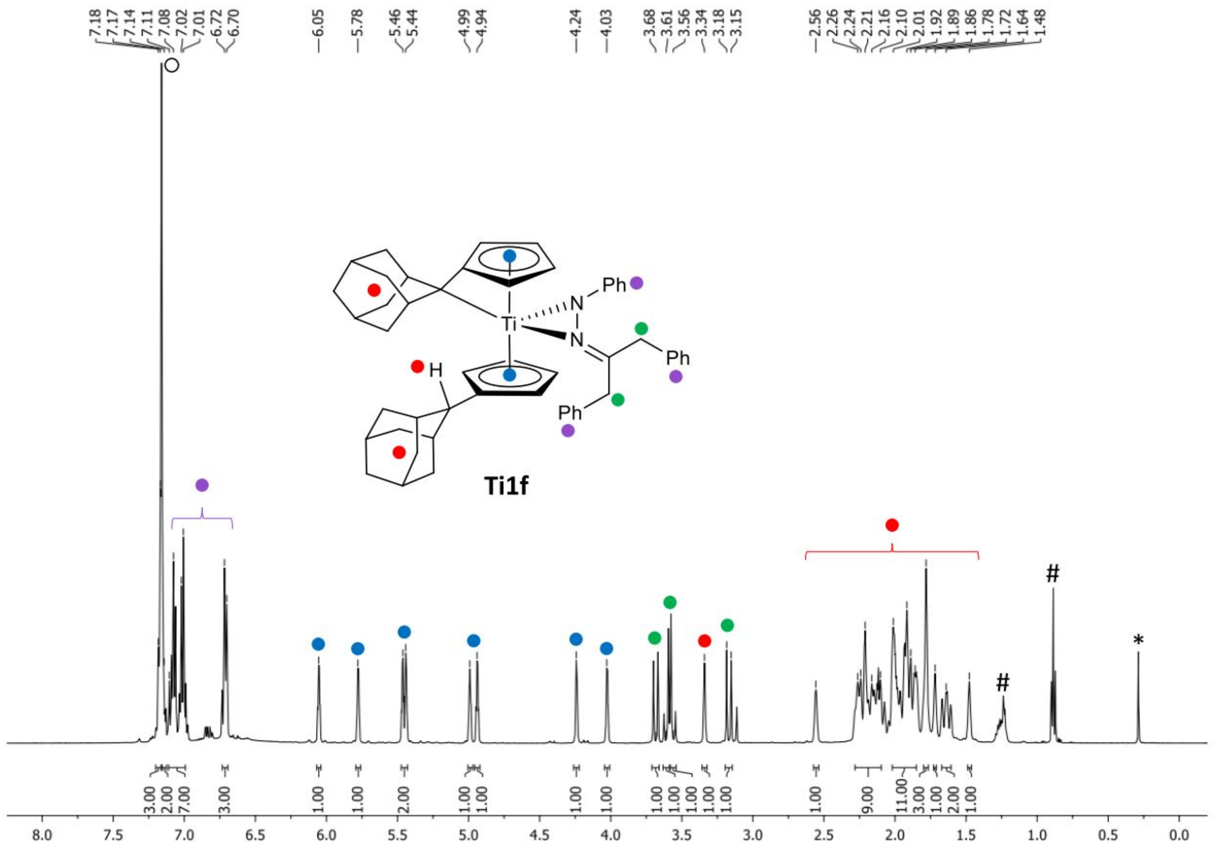

**Figure S11**: $^{1}$H NMR spectrum (500 MHz, $C_6D_6$, 305 K) of **Ti1f**. Product signals given in colours (° = $C_6H_5D$, # = *n*-hexane, * = grease).

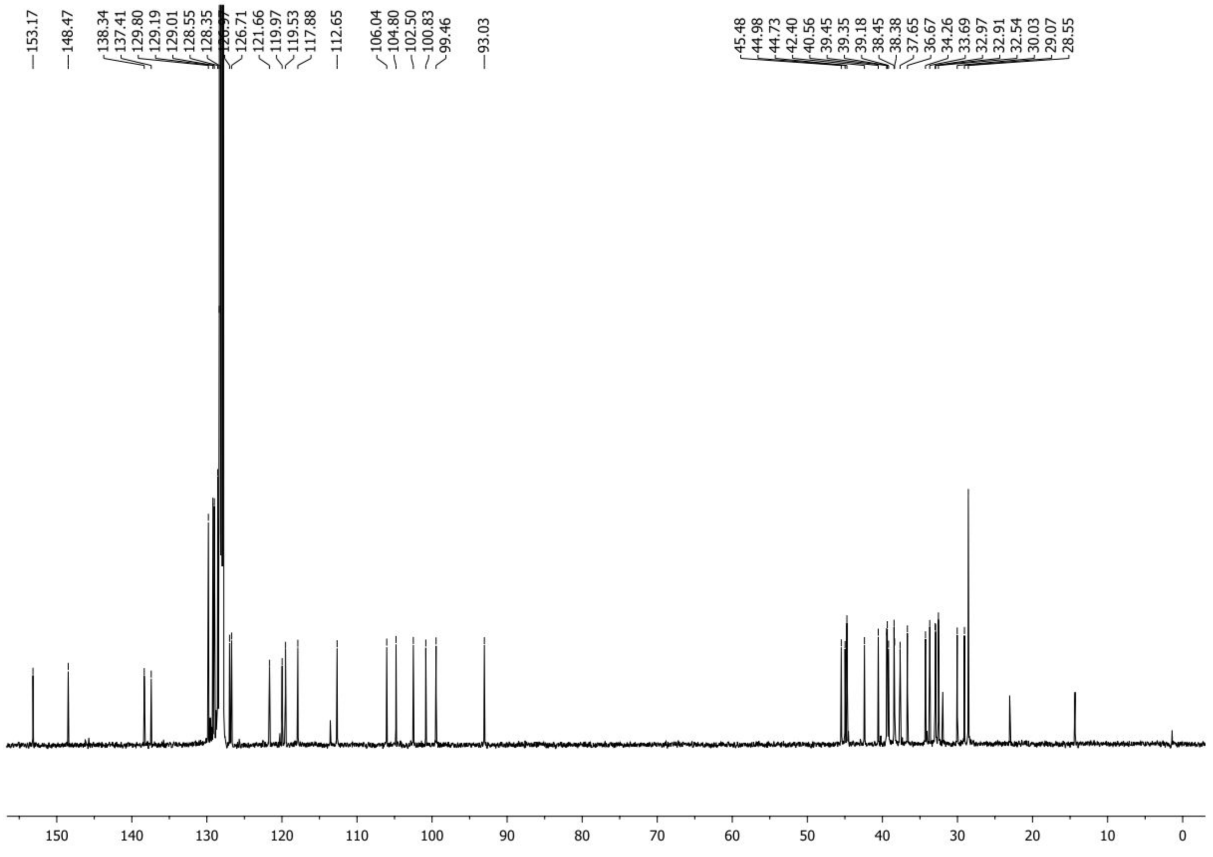

**Figure S12**: $^{13}$C NMR spectrum (125 MHz, $C_6D_6$, 305 K) of **Ti1f**.

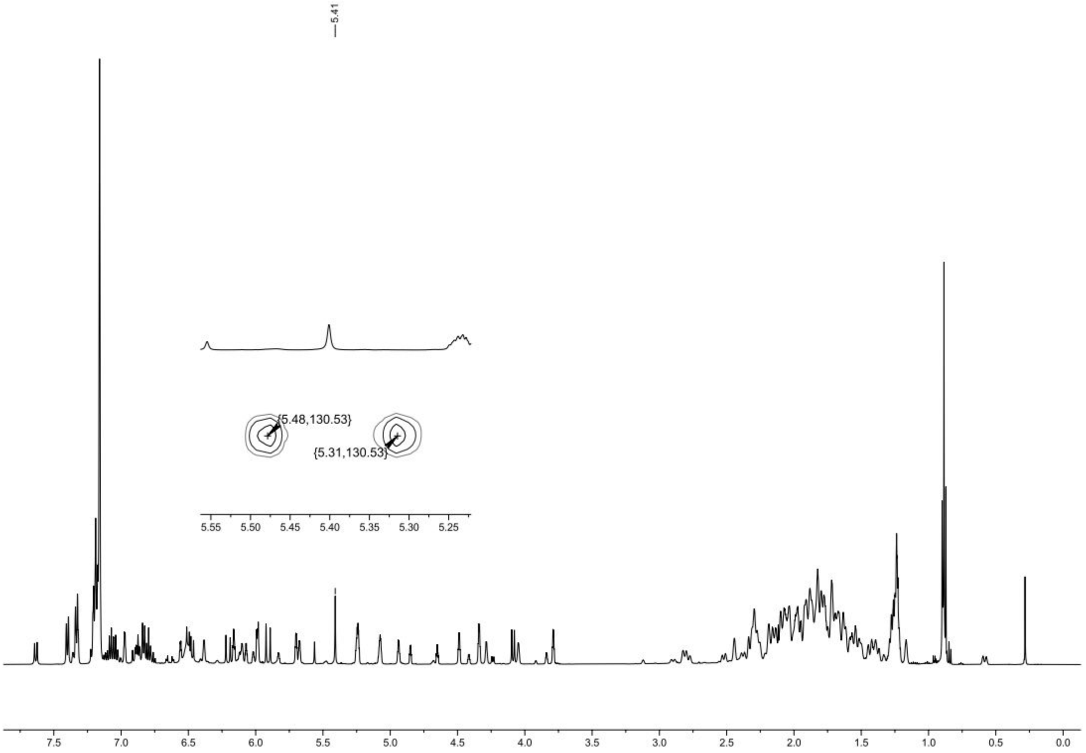


**Figure S13**: $^{1}H$ NMR spectrum (500 MHz, $C_6D_6$, 305 K) of product mixture including **Ti1g**. Small window: excerpt of the corresponding $^{1}H,^{15}N$ HMBC NMR spectrum (51 MHz, $C_6D_6$, 305 K).

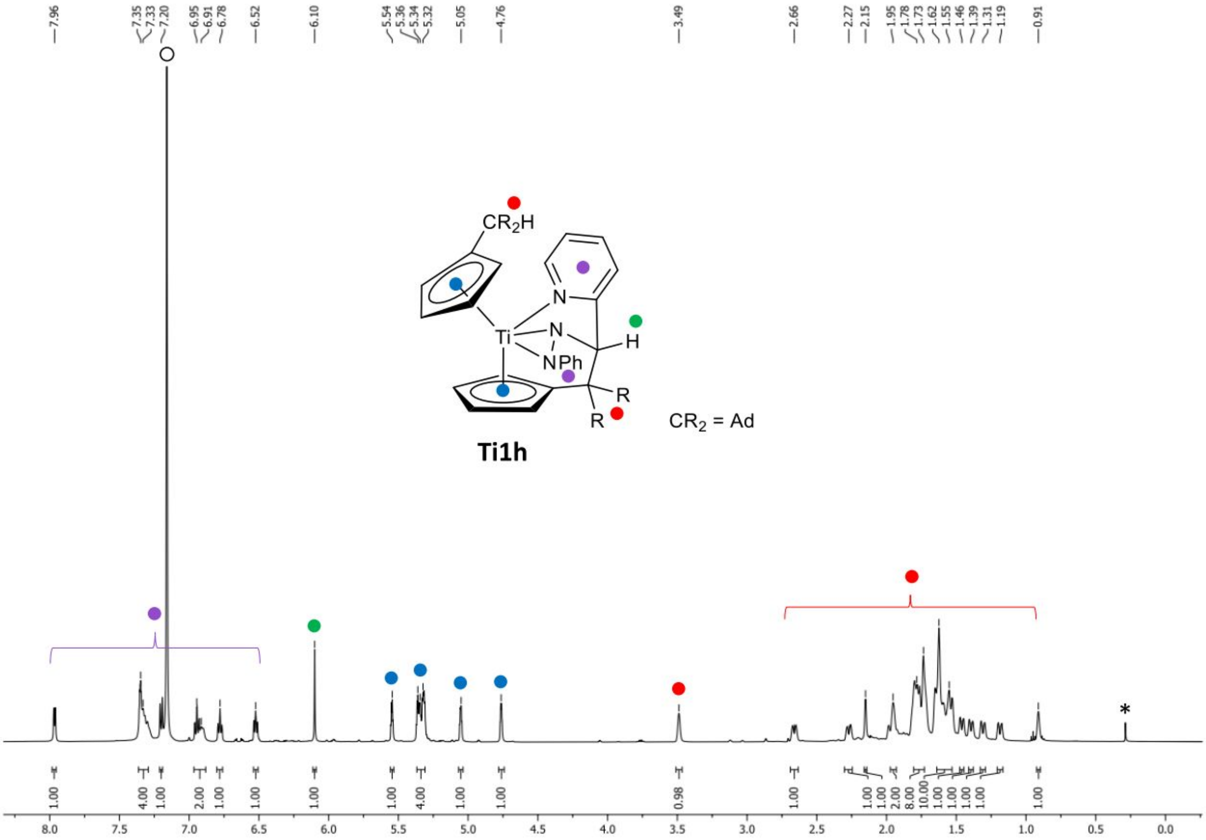


**Figure S14**: $^{1}H$ NMR spectrum (500 MHz, $C_6D_6$, 305 K) of **Ti1h**. Product signals given in colours (° = $C_6H_5D$, * = grease).

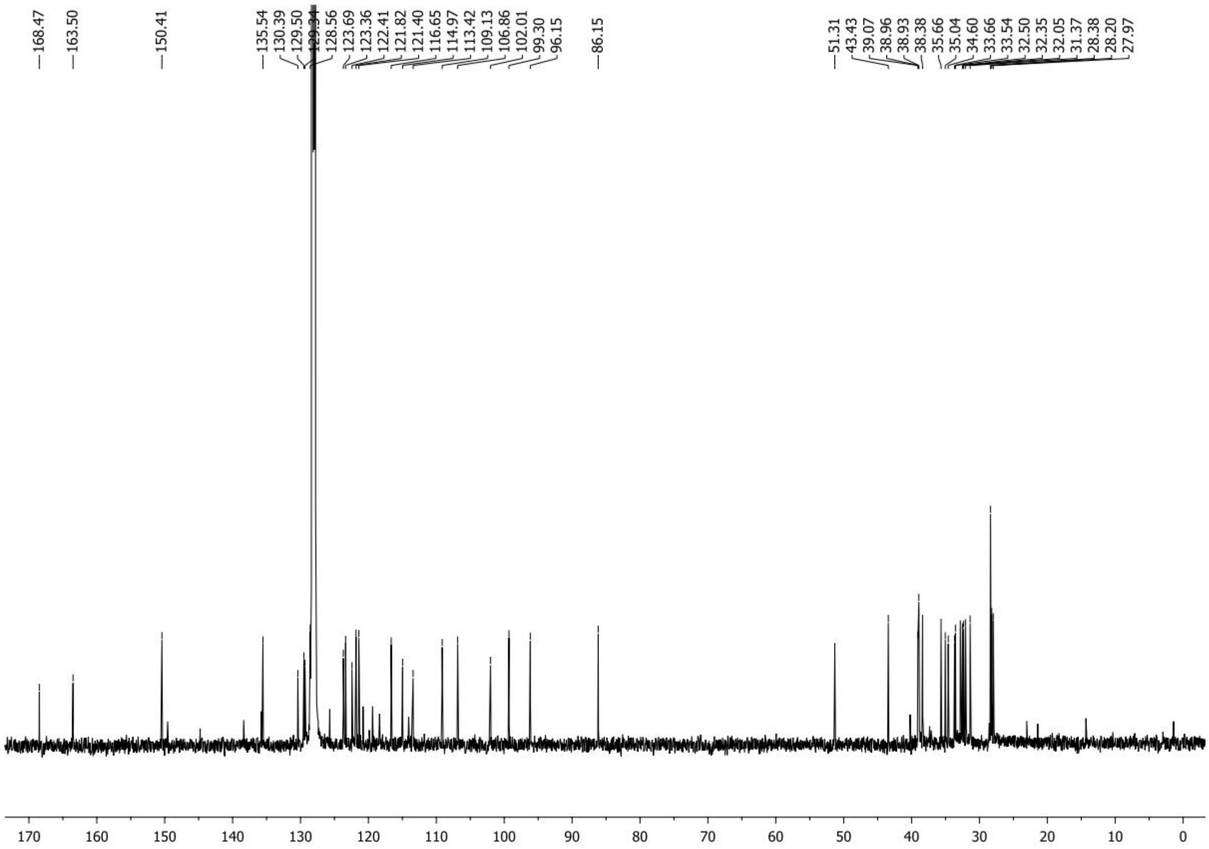


**Figure S15**: $^{13}$C NMR spectrum (125 MHz, $C_6D_6$, 305 K) of **Ti1h**.

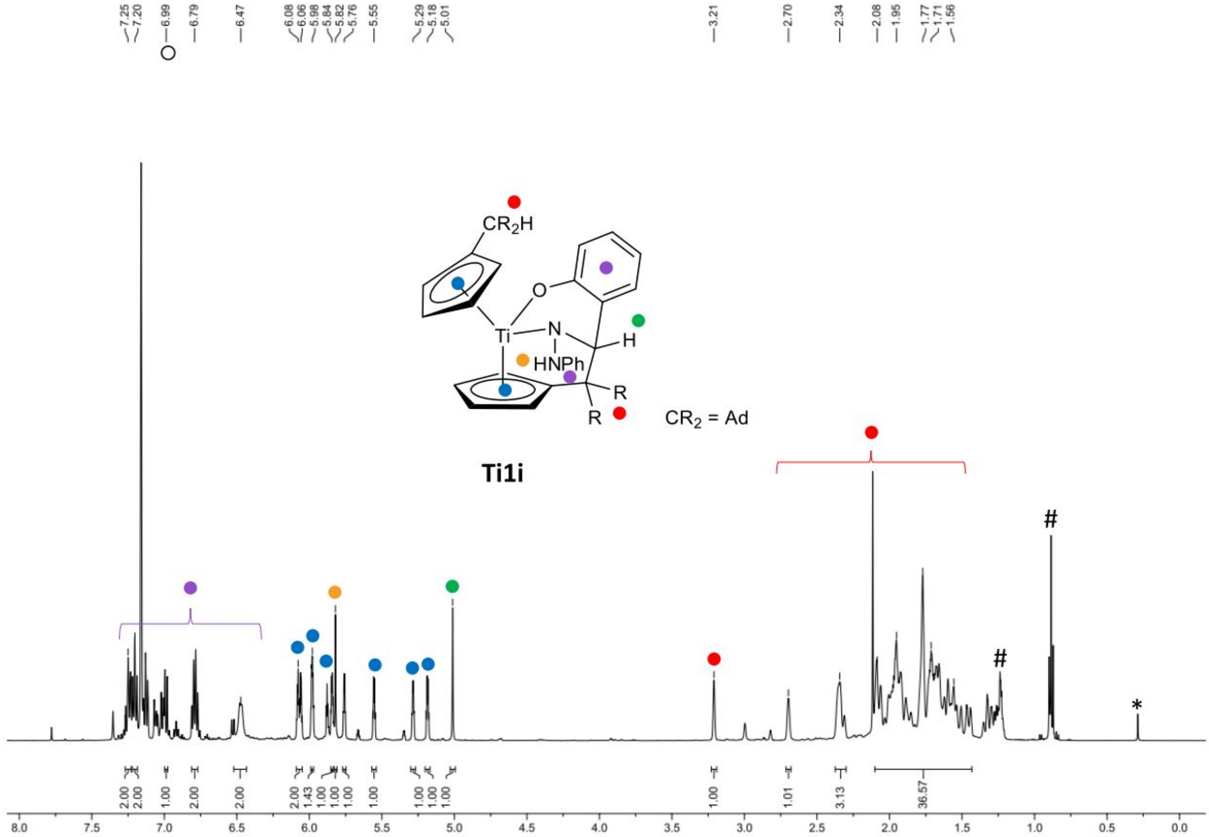


**Figure S16**: $^{1}$H NMR spectrum (500 MHz, $C_6D_6$, 305 K) of **Ti1i**. Product signals given in colours (° = $C_6H_5D$, # = *n*-hexane, * = grease).

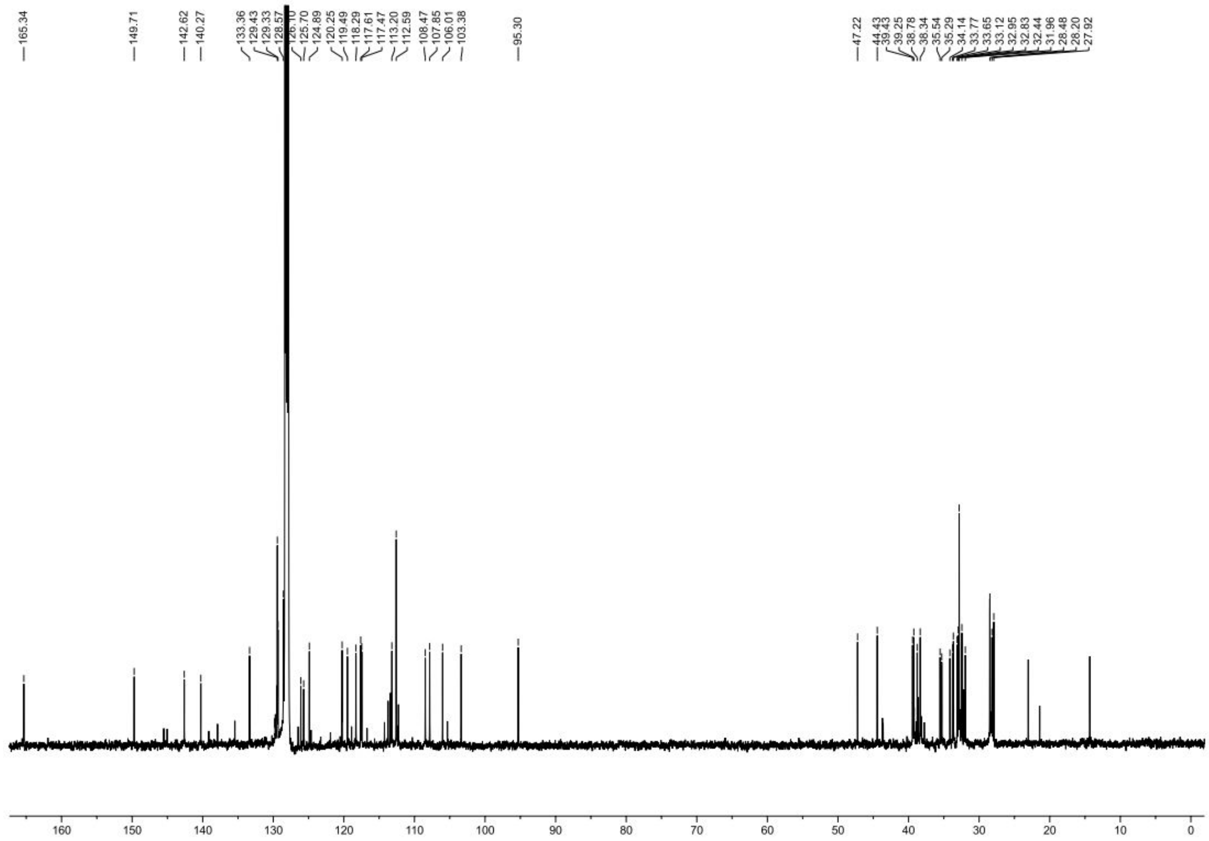

**Figure S17**: $^{13}C$ NMR spectrum (125 MHz, $C_6D_6$, 305 K) of **Ti1i**.

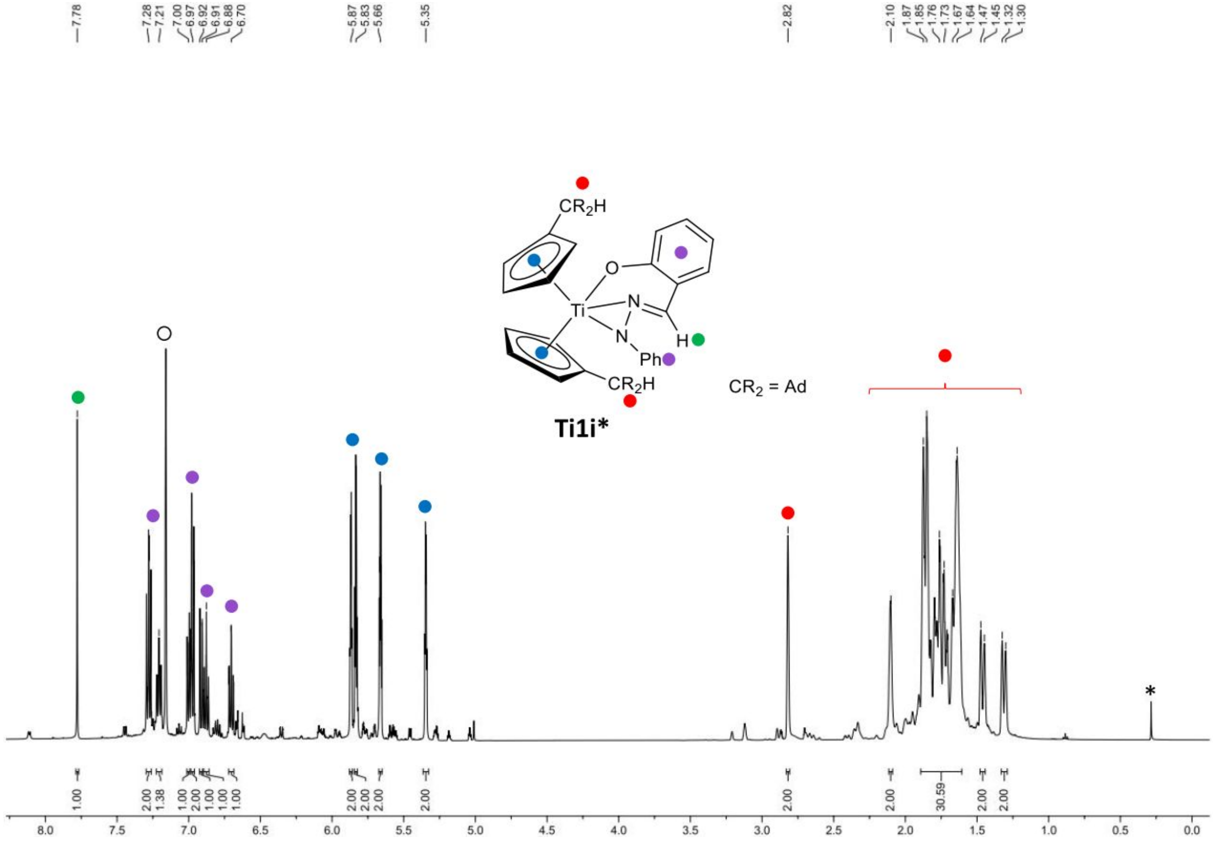


**Figure S18**: $^{1}H$ NMR spectrum (500 MHz, $C_6D_6$, 305 K) of **Ti1i**. Product signals given in colours (° = $C_6H_5D$, * = grease, traces of **Ti1i**).

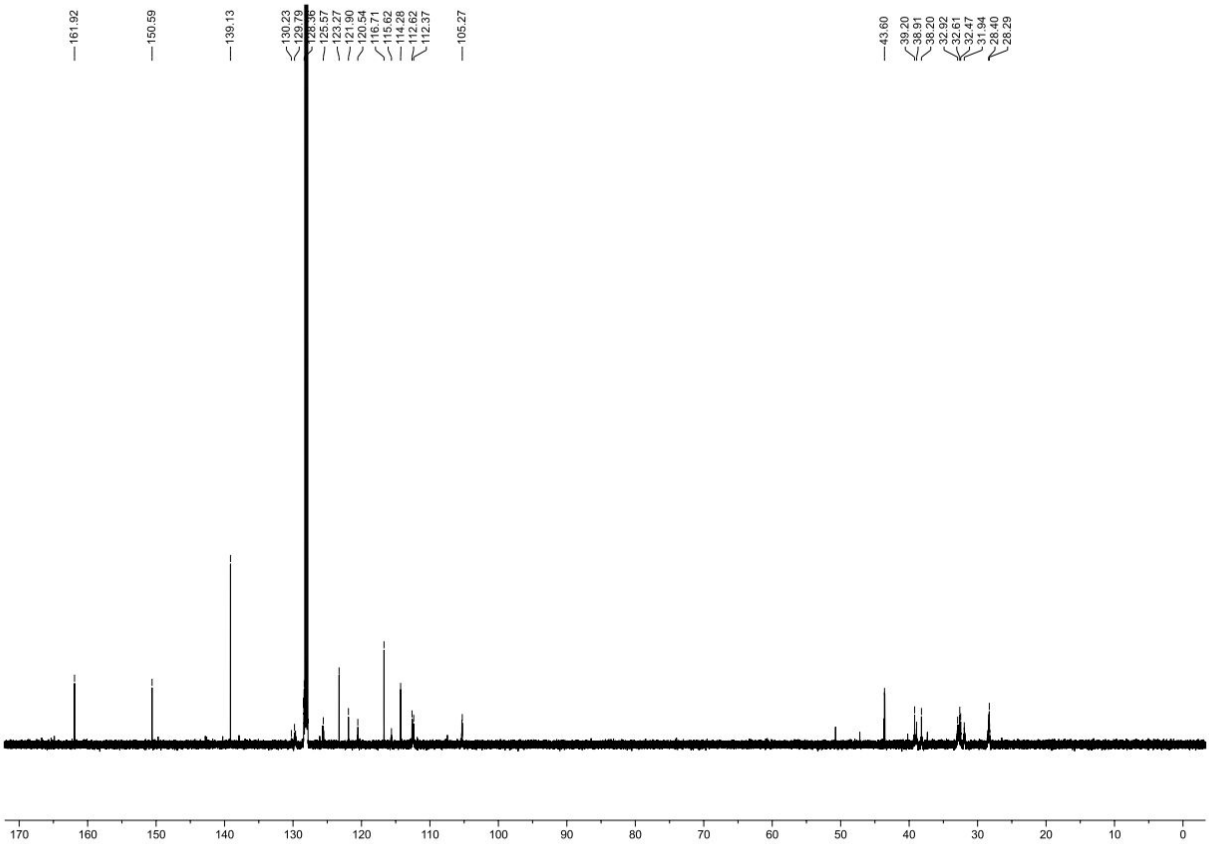

**Figure S19**: $^{13}C$ NMR spectrum (125 MHz, $C_6D_6$, 305 K) of **Ti1i**.

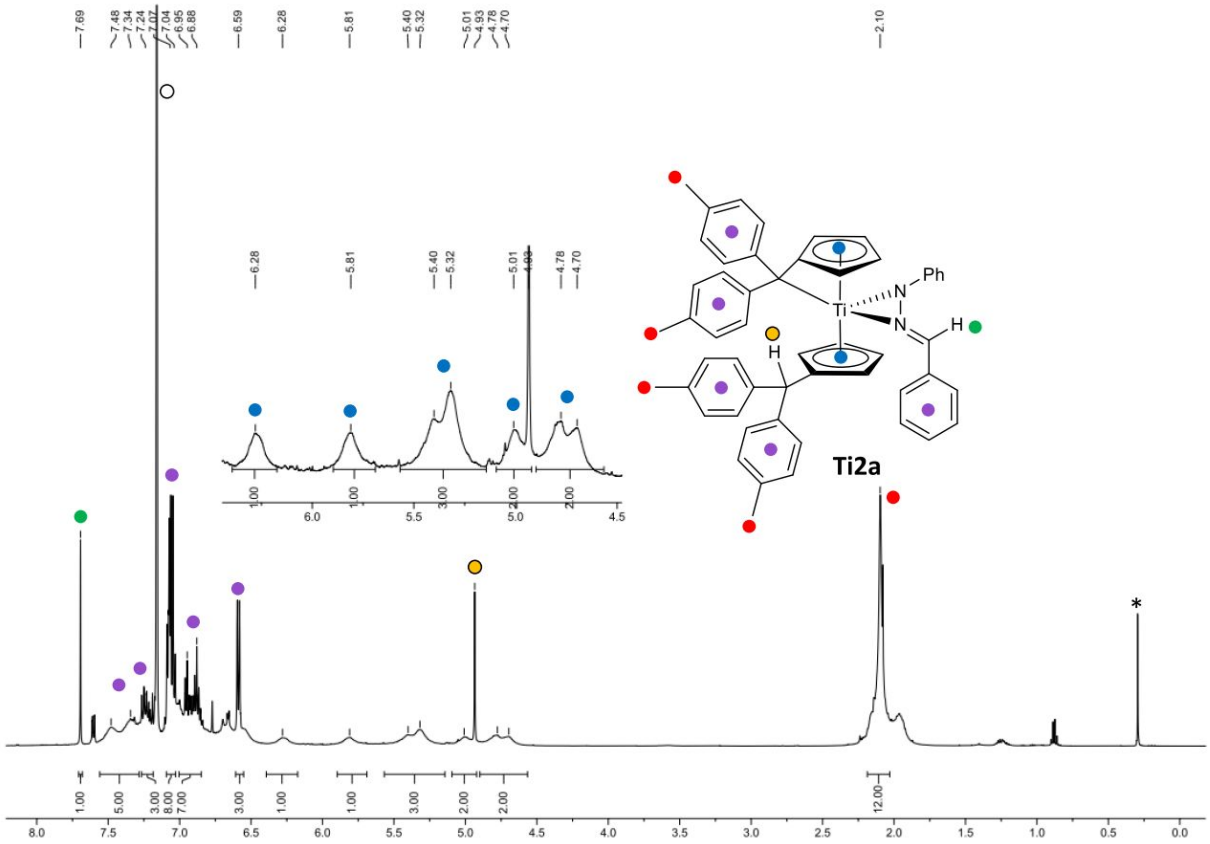

**Figure S20**: $^{1}H$ NMR spectrum (500 MHz, $C_6D_6$, 305 K) of **Ti2a**. Product signals given in colours (° = $C_6H_5D$, # = *n*-hexane, * = grease).

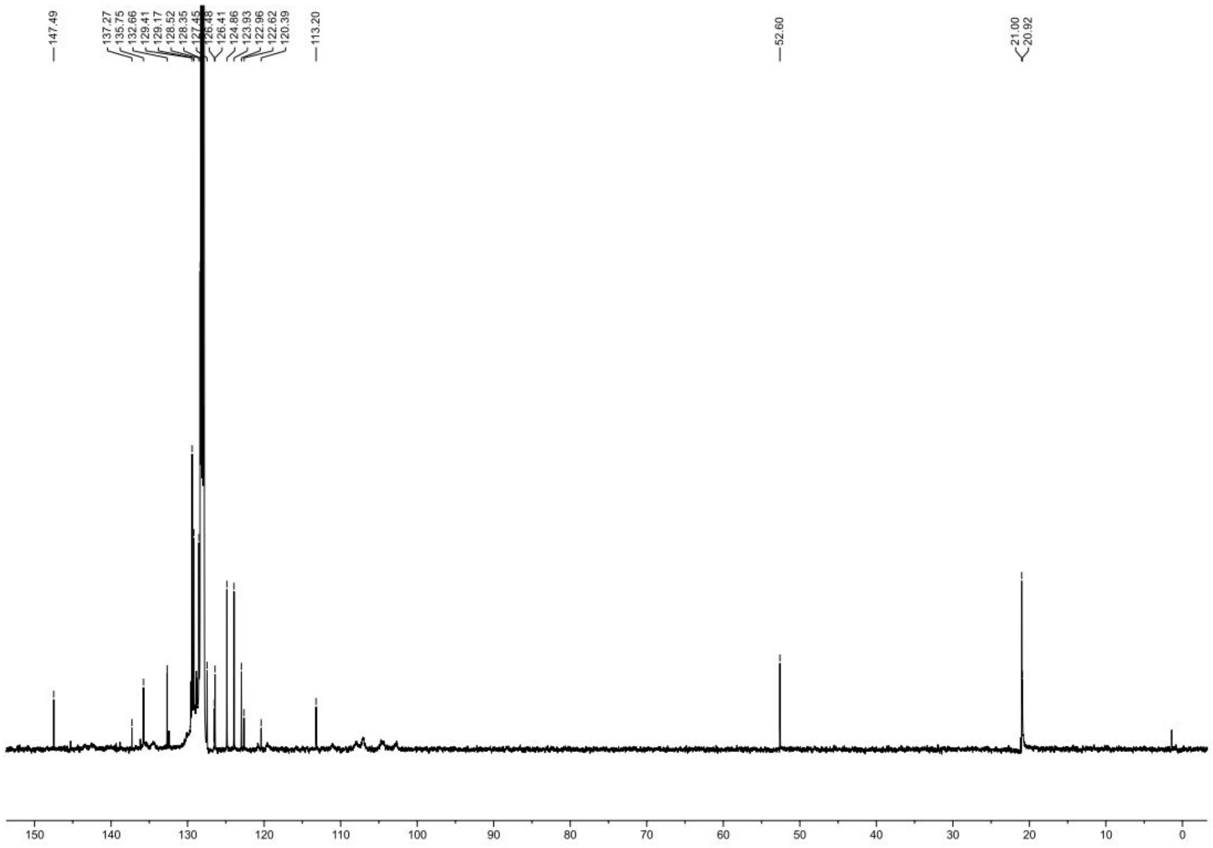


**Figure S21**: $^{13}C$ NMR spectrum (125 MHz, $C_6D_6$, 305 K) of **Ti2a**.

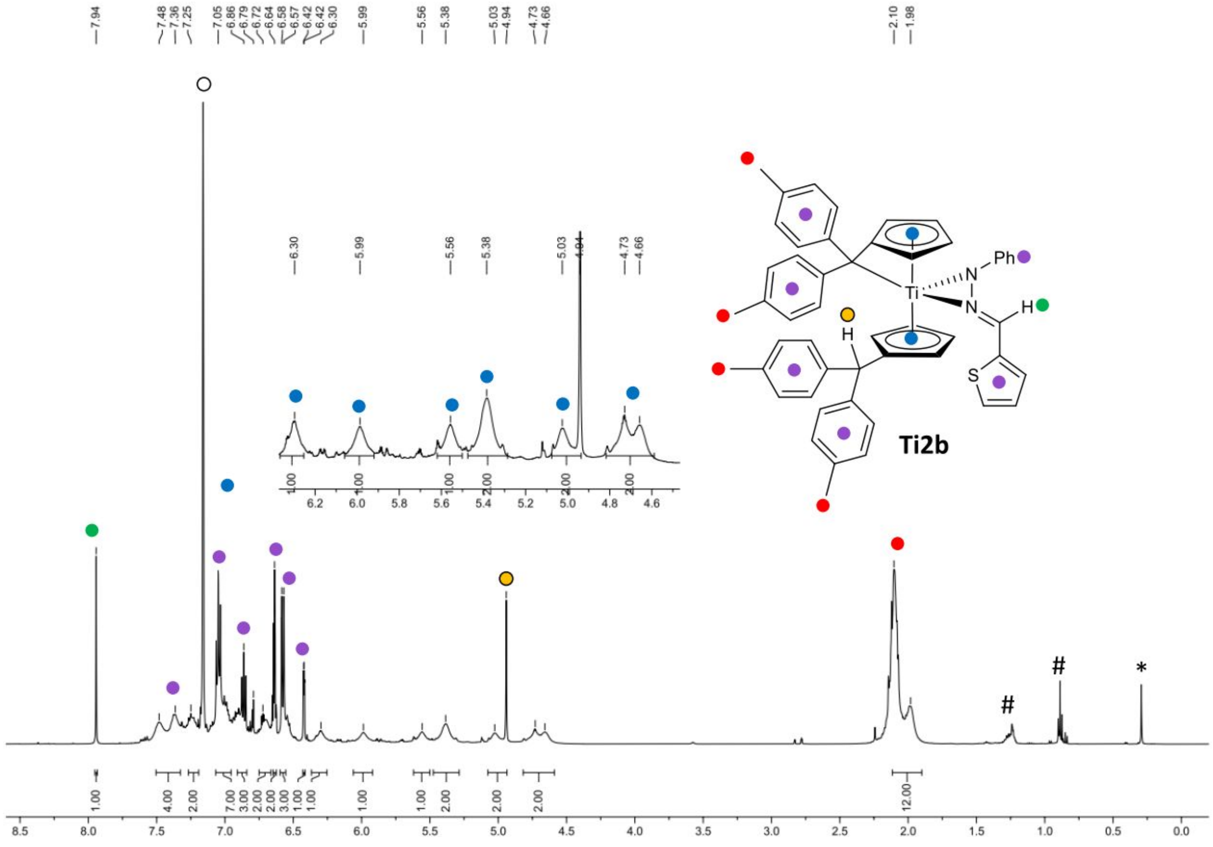


**Figure S22**: $^{1}H$ NMR spectrum (500 MHz, $C_6D_6$, 305 K) of **Ti2b**. Product signals given in colours (° = $C_6H_5D$, # = *n*-hexane, * = grease).

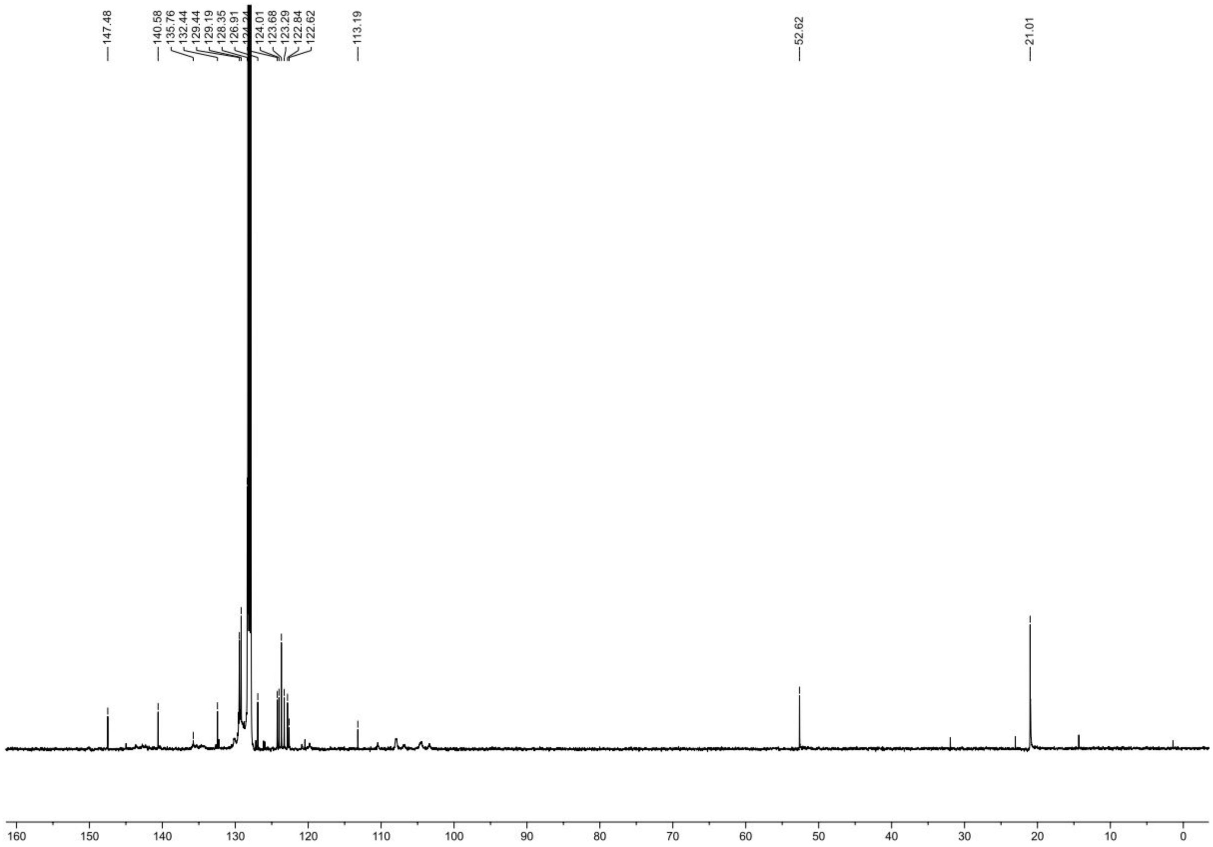


**Figure S23**: $^{13}C$ NMR spectrum (125 MHz, $C_6D_6$, 305 K) of **Ti2b**.

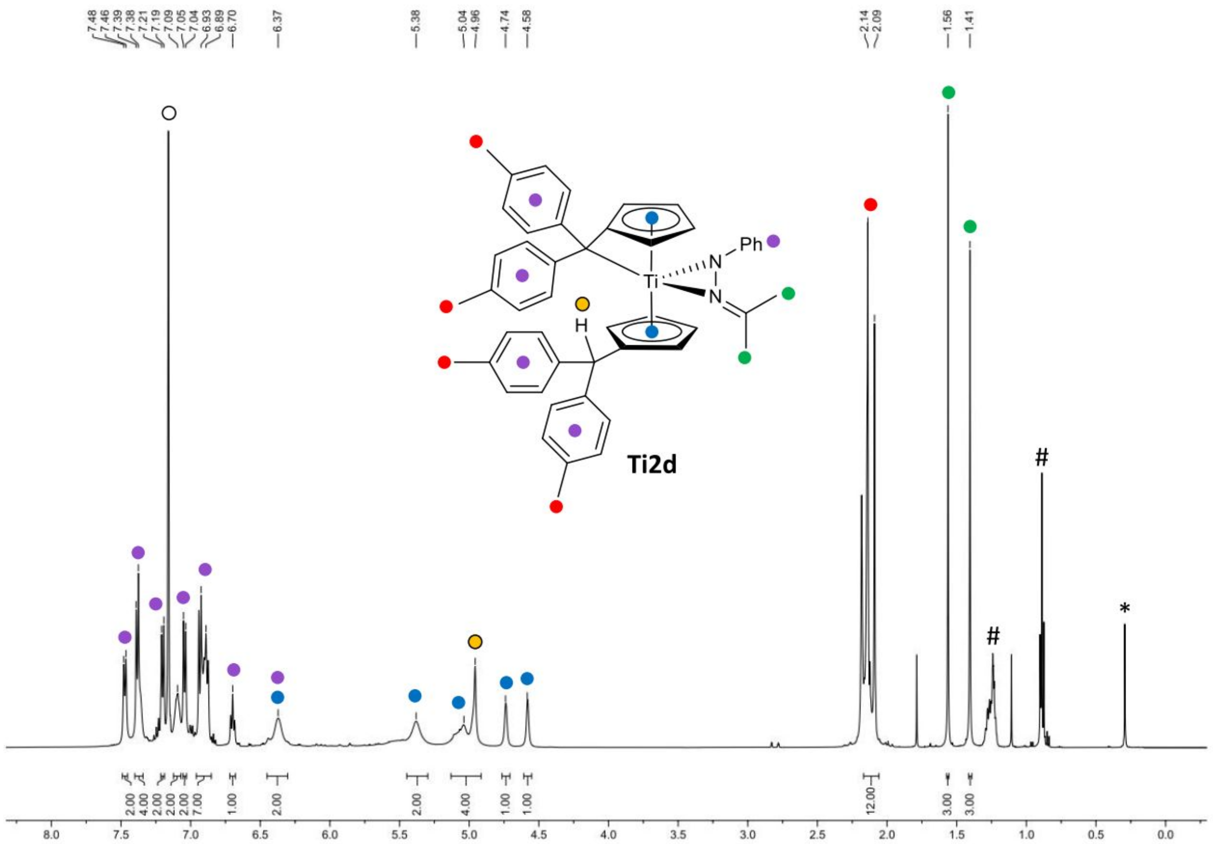


**Figure S24**: $^{1}H$ NMR spectrum (500 MHz, $C_6D_6$, 305 K) of **Ti2d**. Product signals given in colours ($^{o}$ = $C_6H_5D$, # = *n*-hexane, * = grease).

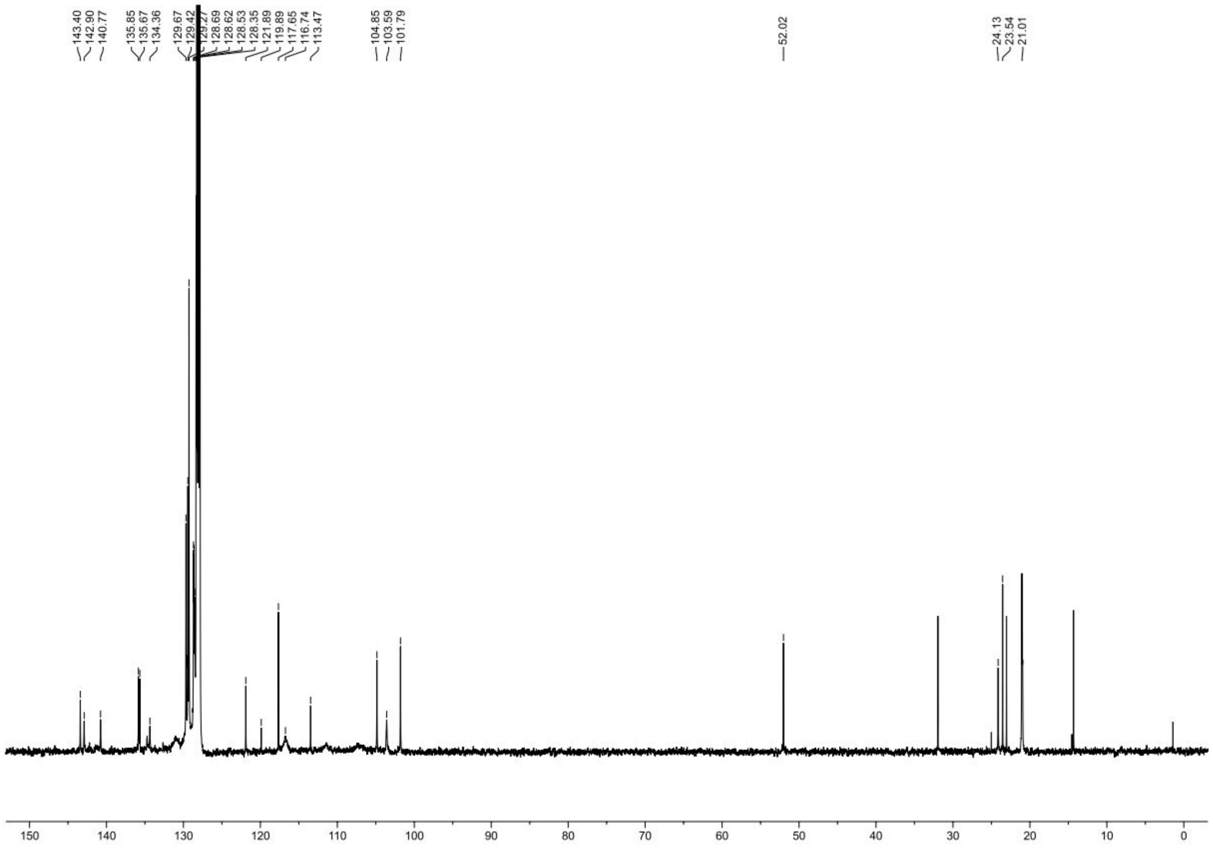

**Figure S25**: $^{13}C$ NMR spectrum (125 MHz, $C_6D_6$, 305 K) of **Ti2d**.

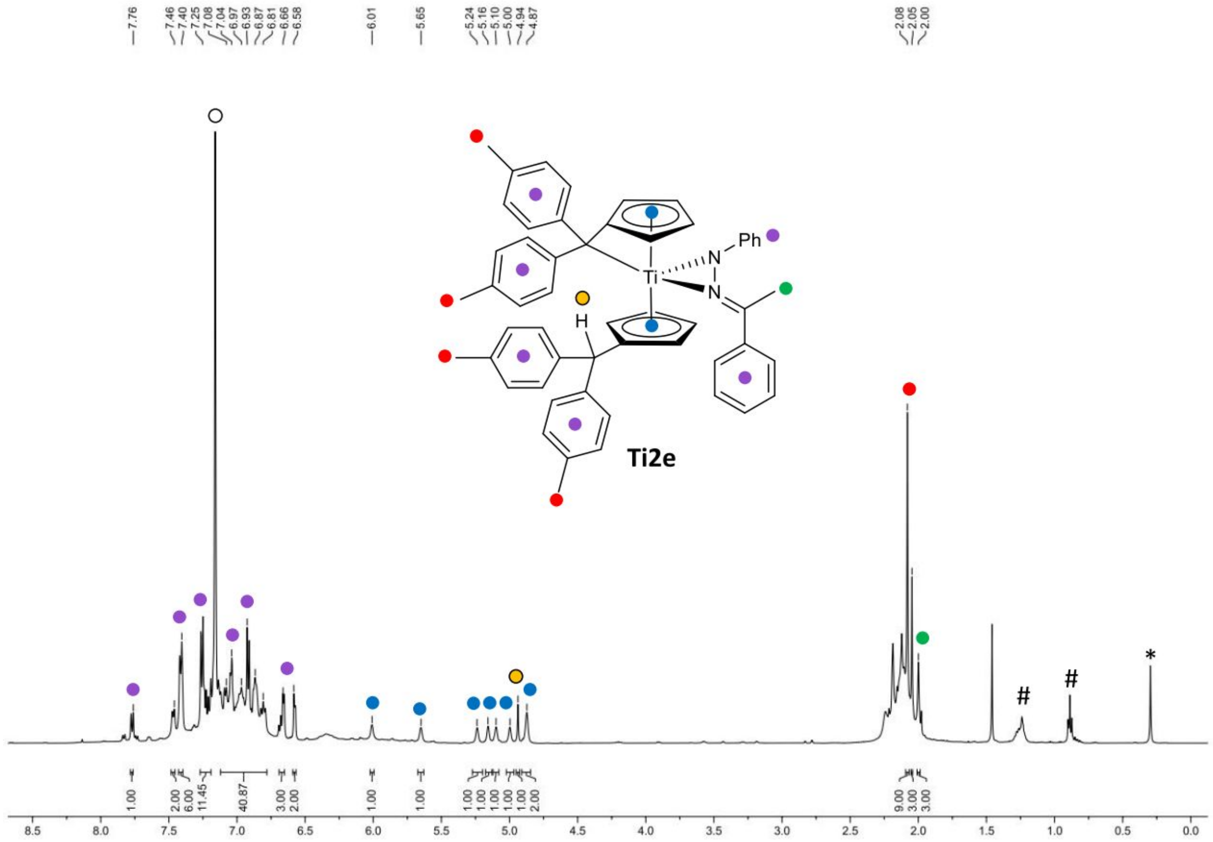


**Figure S26**: $^{1}H$ NMR spectrum (500 MHz, $C_6D_6$, 305 K) of **Ti2e**. Product signals given in colours (° = $C_6H_5D$, # = *n*-hexane, * = grease).

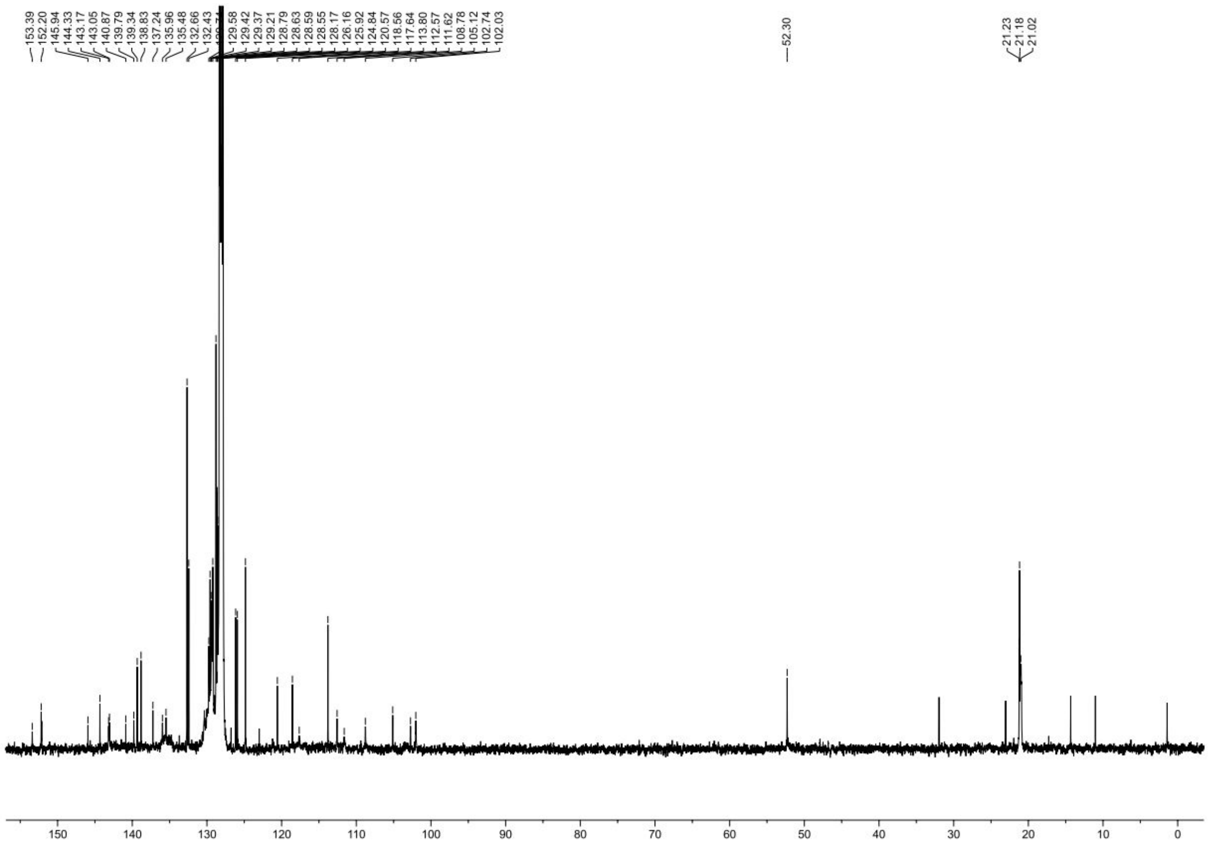

**Figure S27**: $^{13}C$ NMR spectrum (125 MHz, $C_6D_6$, 305 K) of **Ti2e**.

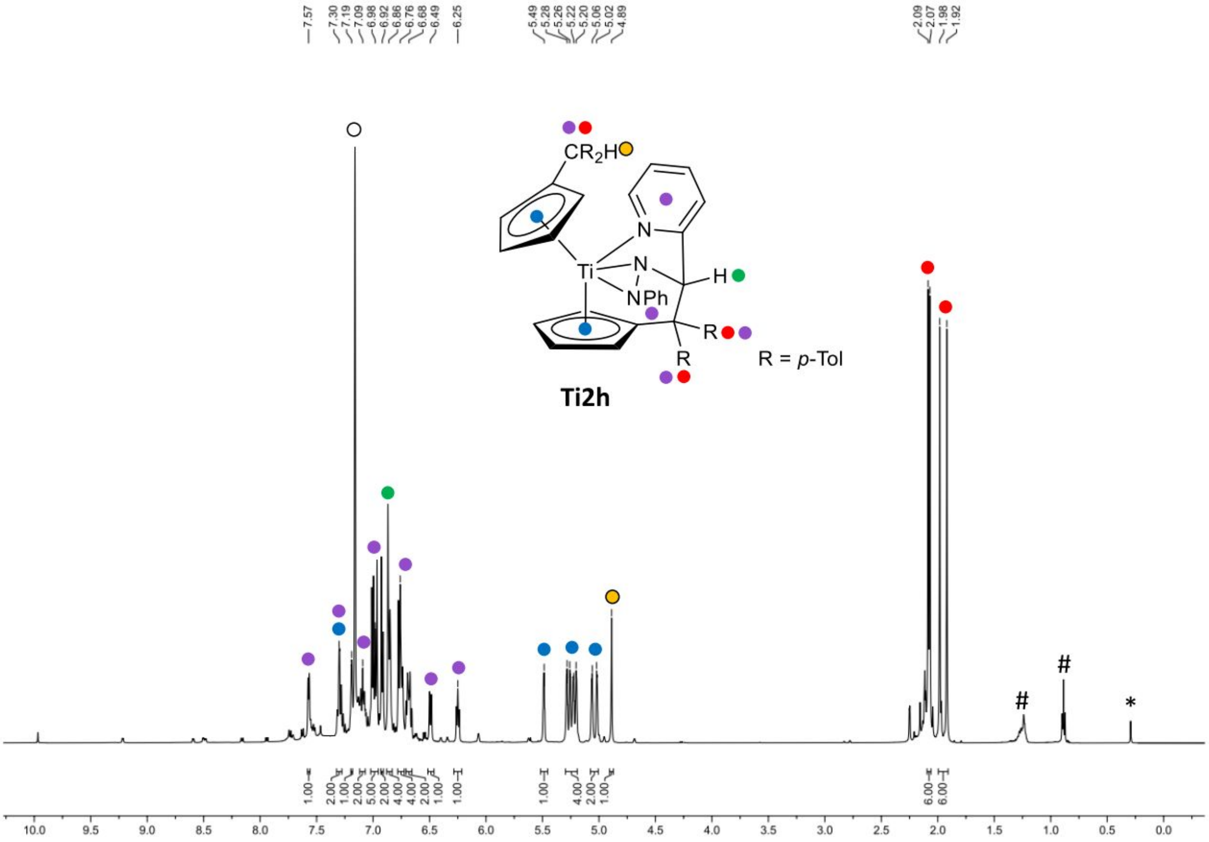

**Figure S28**: $^{1}H$ NMR spectrum (500 MHz, $C_6D_6$, 305 K) of **Ti2h**. Product signals given in colours (° = $C_6H_5D$, # = *n*-hexane, * = grease, traces of byproduct).

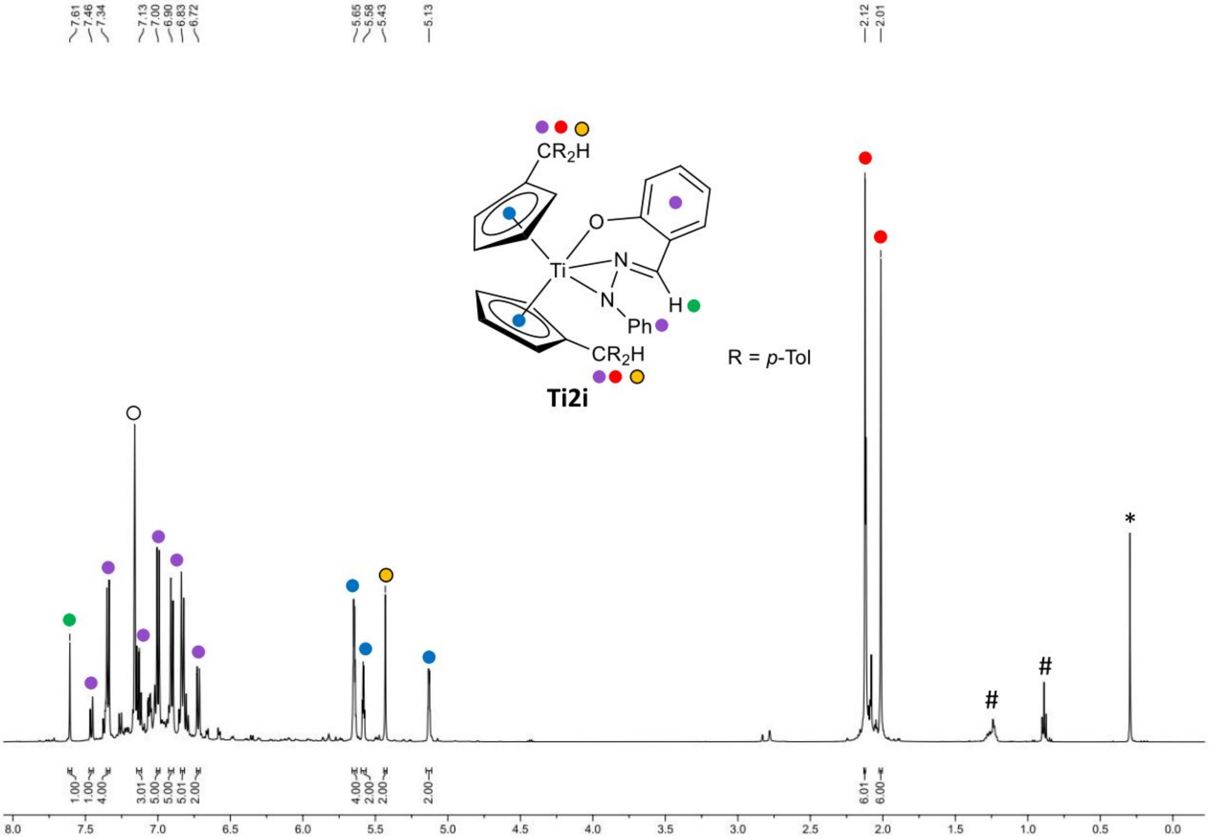


**Figure S29**: $^{1}$H NMR spectrum (500 MHz, $C_6D_6$, 305 K) of **Ti2i**. Product signals given in colours (° = $C_6H_5D$, # = *n*-hexane, * = grease).

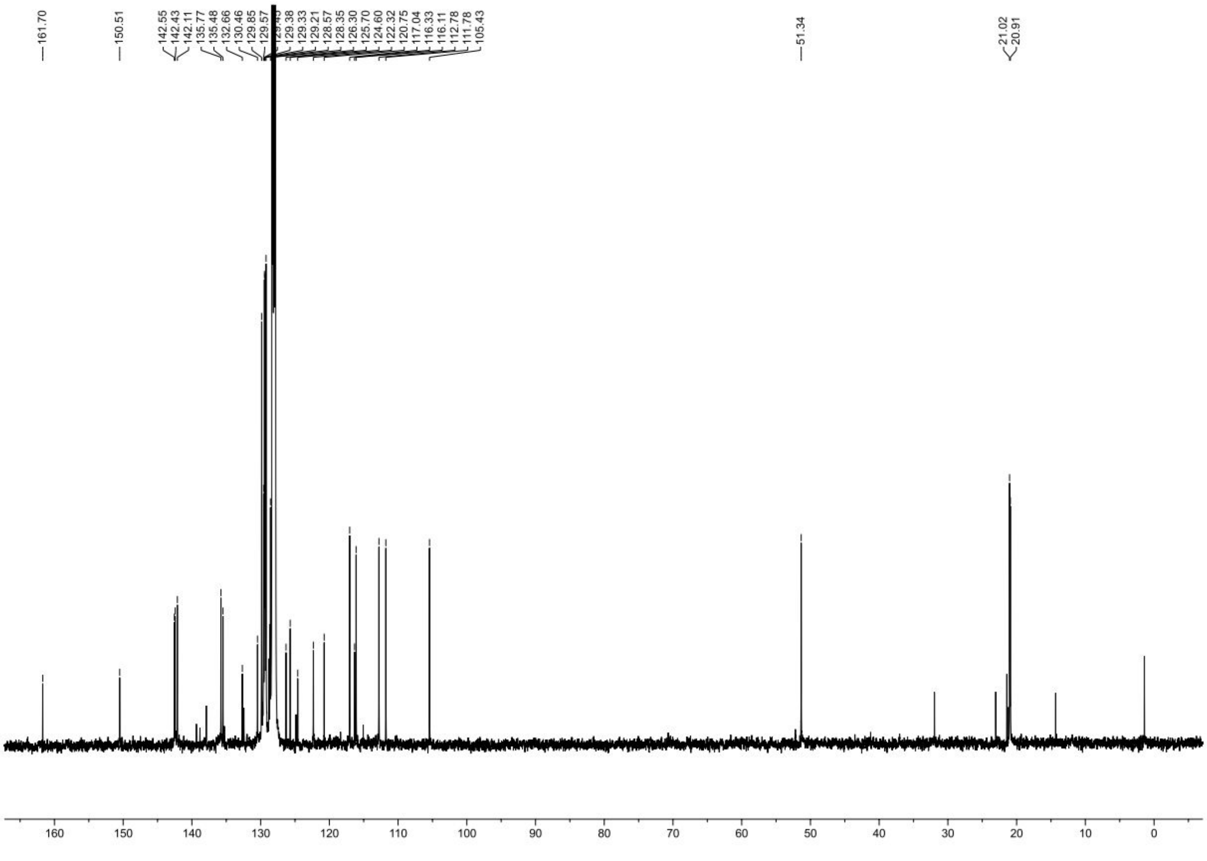


**Figure S30**: $^{13}$C NMR spectrum (125 MHz, $C_6D_6$, 305 K) of **Ti2i**.

## Crystallographic data

Single crystal X-ray data were measured on a Bruker AXS D8 Venture diffractometer (multilayer optics, Mo-Kα and Cu-Kα radiation with λ = 0.71073 Å and 1.54178 Å respectively, Kappa 4-circle goniometer, Photon III C14 CPAD detector). All crystals were measured at a temperature of 100 K except for **Ti1f** which was measured at 140 K because the crystals crack at approximately 130 K. Absorption corrections using equivalent reflections were performed with the program SADABS.[1] All structures were solved with the program SHELXS[2] and refined with SHELXL[3] using the OLEX2[4] GUI.

All non H atoms were refined using anisotropic atomic displacement parameters. H atoms bonded to C were located in the difference Fourier maps and placed on idealized geometric positions with idealized atomic displacement parameters using the riding model. H atoms bonded to N were refined freely. In **Ti1i*** there is a single solvent site shared by toluene and *n*-hexane with an additional disorder by symmetry. It was refined using restraints and constraints on the geometry and atomic displacement parameters.

The crystallographic data can be obtained free of charge from https://www.ccdc.cam.ac.uk/structures/ quoting the CCDC numbers 2300636-2300645.

**Table S1**: Crystallographic data of **Ti1a**, **Ti1b**, **Ti1c**, **Ti1d**, **Ti1f**.

| | **Ti1a** | **Ti1b** | **Ti1c** | **Ti1d** | **Ti1f** |
|---|---|---|---|---|---|
| CCDC | 2300640 | 2300636 | 2300639 | 2300641 | 2300644 |
| Lab-ID | KESC94 | FASA14 | KESC103 | KESC079 | FASA38 |
| empirical formula | $C_{43}H_{48}N_2Ti$ *0.5 $C_6D_6$ | $C_{41}H_{46}N_2STi$ *0.5 $C_6D_6$ | $C_{43}H_{43}F_5N_2Ti$ *0.5 $C_6D_6$ | $C_{39}H_{48}N_2Ti$ | $C_{51}H_{56}N_2Ti$ |
| Fw | 682.80 | 688.83 | 772.76 | 592.69 | 744.87 |
| Colour | red orange | orange | orange | red | red orange |
| Habit | block | plate | rod | block | block |
| cryst. dimens. mm | 0.13 x 0.08 x 0.05 | 0.09 x 0.04 x 0.015 | 0.12 x 0.05 x 0.03 | 0.08 x 0.08 x 0.06 | 0.12 x 0.12 x 0.06 |
| cryst. system | triclinic | triclinic | triclinic | monoclinic | monoclinic |
| space group | P-1 | P-1 | P-1 | $P2_1/c$ | $P2_1/c$ |
| a, Å | 10.4700(4) | 10.0993(5) | 10.9058(6) | 16.6328(5) | 11.4006(3) |
| b, Å | 12.3084(4) | 12.9984(7) | 11.9078(7) | 10.6228(3) | 29.3582(8) |
| c, Å | 14.8396(5) | 13.8875(7) | 15.2808(9) | 18.8473(6) | 12.5142(4) |
| $\alpha$, deg | 97.1787(12) | 98.7796(17) | 96.112(2) | 90 | 90 |
| $\beta$, deg | 103.9287(13) | 101.5427(16) | 104.266(2) | 113.5295(11) | 108.7688(11) |
| $\gamma$, deg | 104.2634(12) | 101.4842(16) | 104.790(2) | 90 | 90 |
| V, $Å^3$ | 1764.06(11) | 1714.92(15) | 1828.88(18) | 3053.19(16) | 3965.8(2) |
| Z | 2 | 2 | 2 | 4 | 4 |
| $D_{calc.}$, g $cm^{-3}$ | 1.285 | 1.334 | 1.403 | 1.289 | 1.248 |
| $\mu$, $mm^{-1}$ | 0.279 | 2.939 | 0.297 | 0.311 | 0.254 |
| T, K | 100(2) | 100(2) | 100(2) | 100(2) | 140(2) |
| $\lambda$, Å | 0.71073 | 1.54178 | 0.71073 | 0.71073 | 0.71073 |
| $\theta$ range, deg | 1.442 – 34.971 | 3.315 – 74.470 | 1.798 – 30.032 | 1.335 – 34.971 | 1.387 – 36.318 |
| reflections collected | 112552 | 48625 | 98394 | 135515 | 206055 |
| Indep. Reflecions R(int) | 15492 0.0314 | 6978 0.0282 | 10710 0.0449 | 13411 0.0609 | 19210 0.0371 |
| Observed reflections (I > 2(I)) | 14284 | 6789 | 9541 | 11495 | 17315 |
| Absorption correction | semi-empirical | semi-empirical | semi-empirical | semi-empirical | semi-empirical |
| max, min transm. | 1.0000, 0.9426 | 1.0000, 0.8855 | 1.0000, 0.9480 | 1.0000, 0.9019 | 1.0000, 0.9513 |
| final R indices [$I>2\sigma(I)$] | R1 = 0.0337, wR2 = 0.0915 | R1 = 0.0291, wR2 = 0.0803 | R1 = 0.0435, wR2 = 0.1006 | R1 = 0.0417, wR2 = 0.0974 | R1 = 0.0390, wR2 = 0.0998 |
| R indices (all data) | R1 = 0.0369, wR2 = 0.0936 | R1 = 0.0298, wR2 = 0.0808 | R1 = 0.0508, wR2 = 0.1041 | R1 = 0.0519, wR2 = 0.1019 | R1 = 0.0443, wR2 = 0.1029 |
| GOF on $F^2$ | 1.067 | 1.069 | 1.094 | 1.095 | 1.080 |
| largest diff peak / hole (e.$Å^{-3}$) | 0.756 / -0.407 | 0.483 / -0.286 | 0.428 / -0.402 | 0.637 / -0.454 | 0.516 / -0.452 |

**Table S2**: Crystallographic data of **Ti1g**, **Ti1h**, **Ti2h**, **Ti1i**, **Ti1i***.

| | **Ti1g** | **Ti1h** | **Ti2h** | **Ti1i** | **Ti1i*** |
|---|---|---|---|---|---|
| CCDC | 2300643 | 2300638 | 2300637 | 2300642 | 2300645 |
| Lab-ID | FASA08 | FASA12 | FASA1310 | KESC149 | KESC149T60 |
| empirical formula | $C_{45}H_{50}N_2Ti$ | $C_{42}H_{47}N_3Ti$ | $C_{52}H_{47}N_3Ti$ | $C_{43}H_{48}N_2OTi$ | $C_{43}H_{48}N_2OTi$, *0.125 $C_7H_8$ *0.125 $C_6H_{14}$ |
| Fw | 666.77 | 641.72 | 761.82 | 656.73 | 679.02 |
| Colour | yellow | red | red | orange red | red |
| Habit | plate | block | rod | block | block |
| cryst. dimens. mm | 0.12 x 0.08 x 0.02 | 0.06 x 0.06 x 0.025 | 0.10 x 0.02 x 0.02 | 0.17 x 0.10 x 0.04 | 0.16 x 0.08 x 0.07 |
| cryst. system | monoclinic | orthorhombic | triclinic | monoclinic | monoclinic |
| space group | $P2_1/n$ | Pbca | P-1 | $P2_1/n$ | C2/c |
| a, Å | 14.1026(12) | 10.7594(3) | 10.9970(5) | 11.9737(4) | 42.882(3) |
| b, Å | 9.7967(8) | 22.7187(6) | 13.8939(6) | 11.9785(4) | 11.1105(6) |
| c, Å | 25.698(2) | 25.7773(7) | 15.5879(7) | 22.5507(8) | 29.3481(17) |
| $\alpha$, deg | 90 | 90 | 102.780(3) | 90 | 90 |
| $\beta$, deg | 105.128(3)) | 90 | 106.441(3) | 102.0935(14) | 99.170(2) |
| $\gamma$, deg | 90 | 90 | 111.124(2) | 90 | 90 |
| V, $Å^3$ | 3427.4(5) | 6301.0(3) | 1986.92(16) | 3162.60(19) | 13804.1(14) |
| Z | 4 | 8 | 2 | 4 | 16 |
| $D_{calc.}$, g $cm^{-3}$ | 1.292 | 1.353 | 1.273 | 1.379 | 1.307 |
| $\mu$, $mm^{-1}$ | 0.286 | 2.568 | 2.126 | 0.311 | 0.287 |
| T, K | 100(2) | 100(2) | 100(2) | 100(2) | 100(2) |
| $\lambda$, Å | 0.71073 | 1.54178 | 1.54178 | 0.71073 | 0.71073 |
| $\theta$ range, deg | 1.507 – 34.971 | 3.429 – 74.494 | 3.170 – 74.482 | 1.790 – 36.318 | 1.406 – 34.970 |
| reflections collected | 193157 | 55893 | 45136 | 196972 | 336226 |
| Indep. Reflecions R(int) | 15056 0.0593 | 6439 0.0592 | 8046 0.0768 | 15327 0.0425 | 30329 0.0474 |
| Observed reflections (I > 2(I)) | 13227 | 5487 | 7069 | 14088 | 26222 |
| Absorption correction | semi-empirical | semi-empirical | semi-empirical | semi-empirical | semi-empirical |
| max, min transm. | 1.0000, 0.9365 | 1.0000, 0.9018 | 1.0000, 0.8054 | 1.0000, 0.9351 | 1.0000, 0.9324 |
| final R indices [$I>2\sigma(I)$] | R1 = 0.0528, wR2 = 0.1228 | R1 = 0.0412, wR2 = 0.1031 | R1 = 0.0561, wR2 = 0.1529 | R1 = 0.0360, wR2 = 0.0910 | R1 = 0.0430, wR2 = 0.1082 |
| R indices (all data) | R1 = 0.0616, wR2 = 0.1263 | R1 = 0.0500, wR2 = 0.1080 | R1 = 0.0629, wR2 = 0.1570 | R1 = 0.0403, wR2 = 0.0932 | R1 = 0.0514, wR2 = 0.1133 |
| GOF on $F^2$ | 1.196 | 1.020 | 1.069 | 1.124 | 1.062 |
| largest diff peak / hole (e.$Å^{-3}$) | 0.637 / -0.491 | 1.017 / -0.560 | 0.412 / -0.356 | 0.698 / -0.606 | 0.751 / -0.559 |

## Molecular structures

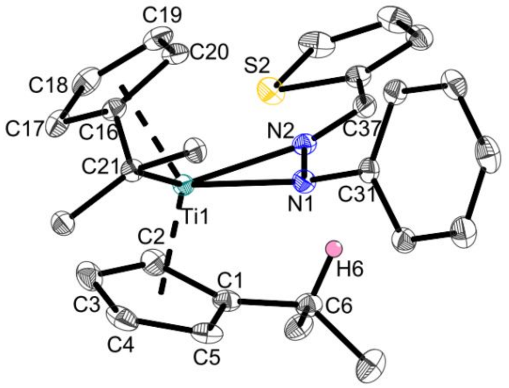


**Figure S31**: Molecular structure of complex **Ti1b**. Displacement ellipsoids are drawn at the 50% probability level. H atoms and solvent molecules have been omitted for clarity. Selected bond lengths (Å) and angles (deg): Ti1–N1 2.0763(10) , Ti1–N2 2.1321(10), Ti1–C21 2.5505, N1–N2 1.3388(14), N1–C37 1.2999(16), N2–C3 11.4108(15) , C21–C16 1.4224(16), N2–H6 2.407, Ti1–N2–N1 69.20(6), Ti1–C21–C16 59.03(6), Ti1–N2–C31 161.60(9), Ct1–Ti1–Ct2 134.5 (Ct1 = centroid of C1–C5; Ct2 = centroid of C16–C20).

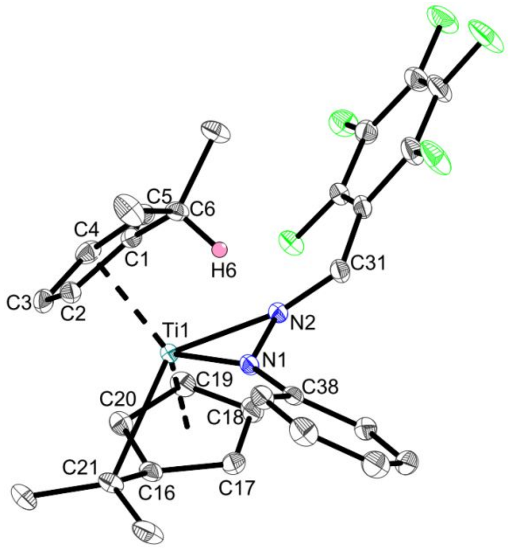


**Figure S32**: Molecular structure of complex **Ti1c**. Displacement ellipsoids are drawn at the 50% probability level. H atoms and solvent molecules have been omitted for clarity. Selected bond lengths (Å) and angles (deg): Ti1–N1 2.0662(11), Ti1–N2 2.1205(11), Ti1–C21 2.5202, N1–N2 1.3285(15), N1–C38 1.4152(17), N2–C31 1.3057(17), C21–C16 1.4253(19), N1–H6 2.358, Ti1–N1–N2 69.29(7), Ti1–C21–C16 59.65(7), Ti1–N2–C31 163.06(10), Ct1–Ti1–Ct2 137.7 (Ct1 = centroid of C1–C5; Ct2 = centroid of C16–C20).

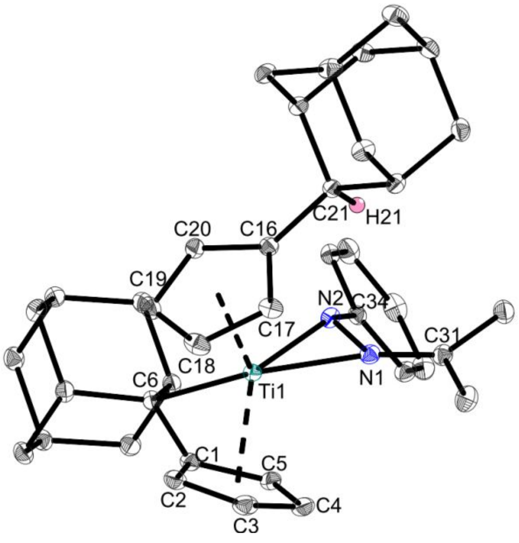


**Figure S33**: Molecular structure of complex **Ti1d**. Displacement ellipsoids are drawn at the 50% probability level. H atoms and solvent molecules have been omitted for clarity. Selected bond lengths (Å) and angles (deg): Ti1–N1 2.0795(8), Ti1–N2 2.1159(8), Ti1–C6 2.5445(9), N1–N2 1.3603(11), N1–C31 1.2904(12), N2–C34 1.4034(12), C1–C6 1.4256(13), N2–H21 2.397, Ti1–N1–N2 72.54(5), Ti1–C6–C1 87.07(6), Ti1–N1–C31 155.52(7), Ct1–Ti1–Ct2 135.6 (Ct1 = centroid of C1–C5; Ct2 = centroid of C16–C20).

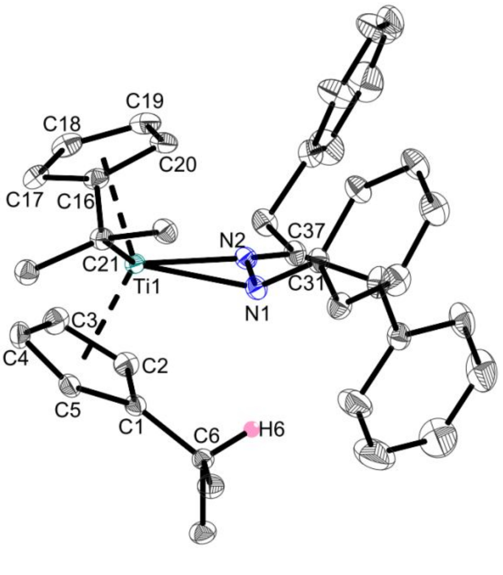


**Figure S34**: Molecular structure of complex **Ti1f**. Displacement ellipsoids are drawn at the 50% probability level. H atoms and solvent molecules have been omitted for clarity. Selected bond lengths (Å) and angles (deg): Ti1–N1 2.1421(6), Ti1–N2 2.0880(6), Ti1–C6 2.593, N1–N2 1.3661(9), N1–C31 1.4028(9), N2–C37 1.2909(9), C1–C6 1.5028(10), N2–H21 2.593, Ti1–N1–N2 69.02(4), Ti1–C6–C1 57.54(4), Ti1–N1–C31 139.88(5), Ct1–Ti1–Ct2 134.9 (Ct1 = centroid of C1–C5; Ct2 = centroid of C16–C20).

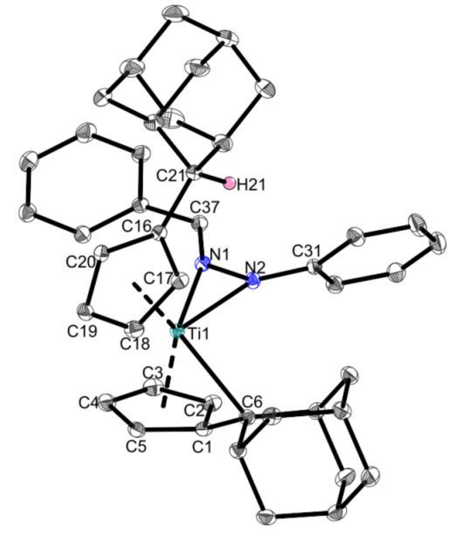


**Figure S35**: Molecular structure of complex **Ti1a**. Displacement ellipsoids are drawn at the 50% probability level. Redundant H atoms and solvent molecules have been omitted for clarity. Selected bond lengths (Å) and angles (deg): Ti1–N1 2.0999(6), Ti1–N2 2.0714(6), Ti1–C6 2.5077(7), N1–N2 1.3409(8), N1–C37 1.2995(9), N2–C31 1.4115(9), C1–C6 1.4263(10), N2–H21 2.408, Ti1–N1–N2 70.10(4), Ti1–C6–C1 85.41(4), Ti1–N1–C31 161.57(5), Ct1–Ti1–Ct2 136.5 (Ct1 = centroid of C1–C5; Ct2 = centroid of C16–C20).

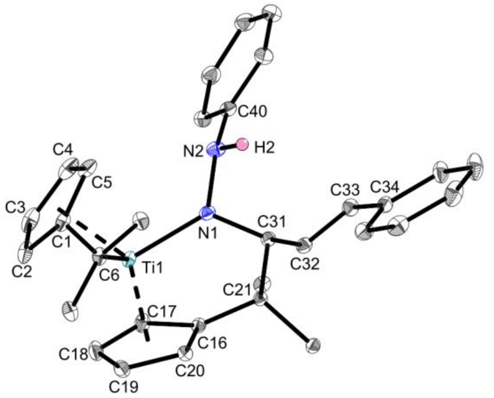


**Figure S36**: Molecular structures of complex **Ti1g**. Displacement ellipsoids are drawn at the 50% probability level. Redundant H atoms and adamantylidene rests have been omitted for clarity. Selected bond lengths (Å) and angles (deg): Ti1–N1 1.9936(11), N1–N2 1.4297(16), Ti1–C6 2.4238(13), N1–C31 1.4967(16), N2–C40 1.4054(18), C1–C6 1.4383(18), C21–C31 1.5655(17), C16–C21 1.5177(17), C31–C32 1.5083(17), C32–C33 1.3393(18), C33–C34 1.4686(18), N2–N1–Ti1 125.02(8), C40–N2–N1 117.49(10), N2–N1–C31 107.66(10), C16–C21–C31 106.00(9), N1–C31–C32 105.96(10), N1–C3–C21 110.82(9), Ct1–Ti–Ct2 137.202 (Ct1 = centroid of C1–C5; Ct2 = centroid of C16–C20).

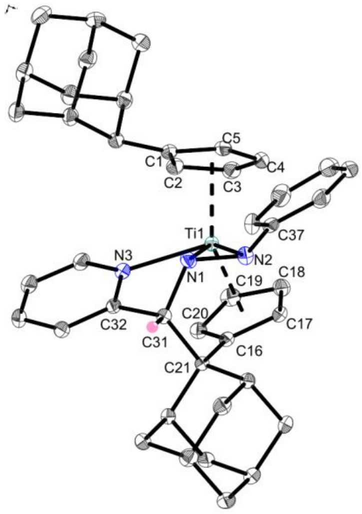


**Figure S37**: Molecular structure of complex **Ti1h**. Displacement ellipsoids are drawn at the 50% probability level. Redundant H atoms have been omitted for clarity. Selected bond lengths (Å) and angles (deg): Ti1–N1 2.0629(16), Ti1–N2 2.0796(16), Ti1–N3 2.2658(15), N1–N2 1.388(2), N1–C31 1.488(2), C31–C21 1.591(2), C21–C16 1.526(2), N(2)–C(37) 1.375(2), N(2)-N(1)-Ti(1) 71.07(9), N2–Ti1–N1 39.15(6), N1–C31–C21 116.22(14), C16–C21–C31 105.41(13), Ct1–Ti–Ct2 134.19.

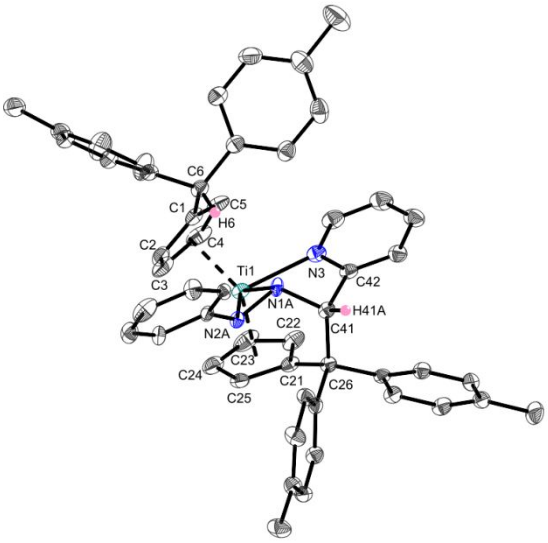


**Figure S38**: Molecular structure of complex **Ti2h**. Displacement ellipsoids are drawn at the 50% probability level. Redundant H atoms have been omitted for clarity. Selected bond lengths (Å) and angles (deg): Ti1–N1A 2.067(6), Ti1–N2A 2.089(3), Ti1–N3 2.275(2), C41–N1A 1.481(6), N3–C42 1.348(3), C41–C42 1.506(3), C26–C41 1.594(3), C21–C26 1.515(3), N1A–Ti1–N3 69.32(13), N1A–Ti1–N2A 38.88(14), N2A–Ti1–N3 107.21(10), Ct1–Ti–Ct2 132.97 (Ct1 = Centroid of C1–C5; Ct2 = Centroid of C16–C20/C21–C25).

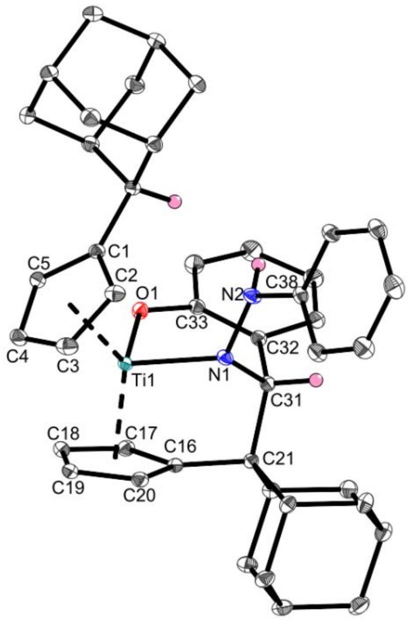


**Figure S39**: Molecular structure of complex **Ti1i**. Displacement ellipsoids are drawn at the 50% probability level. Redundant H atoms have been omitted for clarity. Selected bond lengths (Å) and angles (deg): Ti1–N1 2.0073(6), Ti1–O1 1.9332(6), N1–N2 1.4147(9), N1–C31 1.4894(10), N2–C38 1.3914(10), O1–C33 1.3396(9), C33–C32 1.4204(10), C32–C31 1.5339(10), C31–C21 1.5747(10), O1–H6 2.712, N2–H6 2.588, O1–Ti1–N1 88.33(3), Ct1–Ti1–Ct2 132.1.

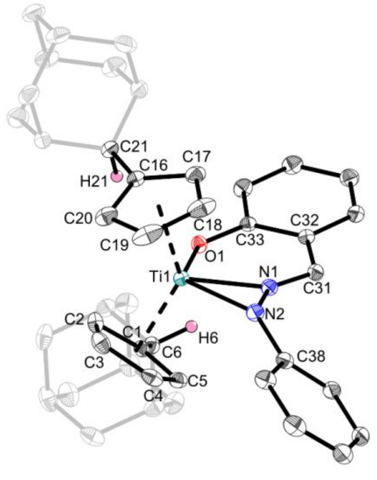


**Figure S40**: Molecular structure of complex **Ti1i***. Displacement ellipsoids are drawn at the 50% probability level. Redundant H atoms have been omitted for clarity. Selected bond lengths (Å) and angles (deg): Ti1–N1 2.0016(8), Ti1–N2 2.1485(8), Ti1–O1 2.0221(7), N1–N2 1.3283(11), O1–C33 1.3197(11), N1–C31 1.2904(12), N2–C38 1.3888(12), C31–C32 1.4471(13), C32–C33 1.4285(13), O1–H6 2.418, O1–H21 2.419, Ti1–N1–N2 77.46(5), Ti1–N1–C31 149.50(7), O1–Ti1–N1 79.38(3), Ct1–Ti1–Ct2 132.0 (Ct1 = centroid of C1–C5; Ct2 = centroid of C16–C20).

## References


[1] L. Krause, R. Herbst-Irmer, G. M. Sheldrick, D. Stalke; Comparison of silver and molybdenum microfocus X-ray sources for single-crystal structure determination. J. Appl. Cryst. **2015**, *48*, 3-10.

[2] G. Sheldrick; A short history of SHELX. Acta Cryst. A **2008**, 64, 112-122.

[3] G. Sheldrick; Crystal structure refinement with SHELXL. Acta Cryst. C **2015**, 71, 3-8.

[4] O. V. Dolomanov, L. J. Bourhis, R. J. Gildea, J. A. K. Howard, H. Puschmann; OLEX2: a complete structure solution, refinement and analysis program. J. Appl. Cryst. **2009**, *42*, 339-341.
